# Enantioselective, Decarboxylative (3+2)-Cycloaddition of Azomethine Ylides and Chromone-3-Carboxylic Acids

**DOI:** 10.3390/molecules27206809

**Published:** 2022-10-11

**Authors:** Ewelina Kowalska, Lesław Sieroń, Anna Albrecht

**Affiliations:** 1 Institute of Organic Chemistry, Faculty of Chemistry, Lodz University of Technology, Żeromskiego 116, 90-924 Łódź, Poland; 2 Institute of General and Ecological Chemistry, Faculty of Chemistry, Lodz University of Technology, Żeromskiego 116, 90-924 Łódź, Poland

**Keywords:** decarboxylation, (3+2)-cycloaddition, chromone-3-carboxylic acid, organocatalysis

## Abstract

Herein, we describe the synthesis of a variety of chiral hybrid pyrrolidine-chromanone polycyclic derivatives. A convenient (3+2)-annulation of azomethine ylides with chromone-3-carboxylic acid realized under Brønsted base catalysis produced highly functionalized products in high yields with good stereoselectivities through asymmetric, intermolecular, and decarboxylative (3+2)-cyclization.

## 1. Introduction

**Scheme 1:**
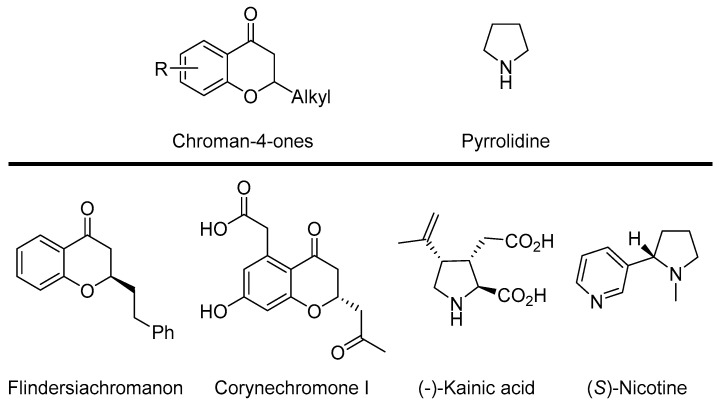
Representative examples of biologically relevant chroman-4-ones or pyrrolidine derivatives.

Chroman-4-ones and related compounds are found in different bioactive molecules relevant to the life-science industry [1,2,3,4,5,6,7]. Although these compounds are abundant in nature, the synthetic methods for their preparation are not very common. The main representatives of this class of compounds are two natural products: Flidersiachromon [4] isolated from the bark of *Flindersia laevicarpa* and Corynechromone I derived from the fungus *Corynespora cassiicola* [5] (Scheme 1).

On the other hand, pyrrolidine derivatives have been extensively exploited because of their applications as bioactive natural products, pharmaceuticals, and potential drug candidates, [8,9,10,11,12,13,14,15,16,17] their wide applications as chiral ligands, [18,19] and their use as organocatalysts [20,21,22]. A pyrrolidine ring is the main constituent of Kainic acid, a natural product that can be found in marine algae, well recognized for its anthelmintic activity [14,15]. Another type of natural product with a pyrrolidine moiety is nicotine. It possesses antioxidant, anti-inflammatory, and antihyperglycemic properties [17,23]. 

**Scheme 2:**
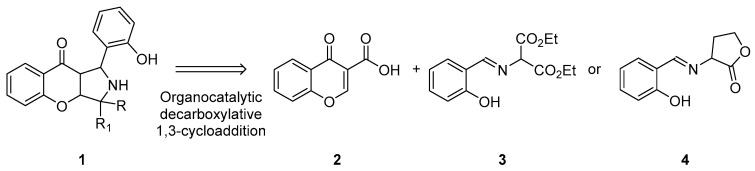
Synthetic objective of the present work.

Due to the high significance of these two classes of compounds in drug discovery, the synthesis of compounds containing both of these two moieties is highly desirable [24,25,26,27,28,29]. To achieve this goal, decarboxylative 1,3-dipolar cycloaddition (1,3-DC) between carboxylic-acid-group-activated olefins and azomethine ylides was devised [23,30,31,32,33,34,35,36,37,38] (Scheme 2). Decarboxylative reactions constitute a very useful strategy in organic synthesis including stereoselective approaches [39,40,41,42]. They rely on the application of carboxylic-acid-activated Michael acceptors or donors. In such a setup, a carboxylate moiety serves a double purpose. It enhances the electrophilic or nucleophilic properties of the starting material and creates the opportunity for its facile removal via decarboxylation.

Herein, we present our studies on decarboxylative (3+2)-cycloaddition between chromone-3-carboxylic acids **2** (acting as electron-poor dipolarophiles) and azomethine ylides **3** or **4** derived from salicylaldehydes and appropriate amines (acting as a dipol) proceeding under mild, basic conditions [43,44]. Our studies demonstrate that the presence of a carboxylic acid group is beneficial for the process, providing an alternative method for the preparation of hybrid molecules **1** containing chromenopyrrolidine units with a quaternary stereogenic center, in some cases. 

## 2. Results

Initially, the quinine-catalyzed cycloaddition between chromen-4-one **5** and imine **3a** was attempted (Table 1, entry 1). Disappointingly, no reaction was observed. Therefore, the activation of **2a** through the introduction of the carboxylic acid moiety was attempted. We were pleased to observe that the devised decarboxylative cycloaddition with chromone-3-carboxylic acid **2a** proceeded efficiently and with concomitant decarboxylation (Table 1, entry 2). Importantly, both the efficiency and the diastereoselectivity of the process were excellent. However, the enantioselectivity required further optimization. Consequently, in the first step, five different catalysts were tested (Table 1, entries 2–6). Optimization studies indicated that bifunctional cinchona alkaloid derivatives **6b-e** (Table 1, entries 3–6) were better when compared to simple alkaloids such as quinine **6a** (Table 1, entry 2). It was found that the presence of a strong H-bonding unit in the catalyst structure was beneficial for the enantiomeric ratio. Unfortunately, a significant decrease in the diastereoselectivity of the cycloaddition was noted. Subsequently, solvent screening using **6c** as the catalyst was performed (Table 1, entries 7–10). As a consequence, CHCl_3_ was identified as the best solvent for the optimized cascade (Table 1, entry 8). The temperature screening (Table 1, entries 8, 11, 12) indicated that the enantioselectivity of the process can be slightly enhanced by lowering the reaction temperature to 0 °C (Table 1, entry 11). Further reduction in the temperature to −20 °C did not affect the enantiomeric excess (Table 1, entry 12). Importantly, both the amount of solvent used and the catalyst loading can be reduced without any effect on the reaction outcome (Table 1, entries 12–16).

**Scheme 3:**
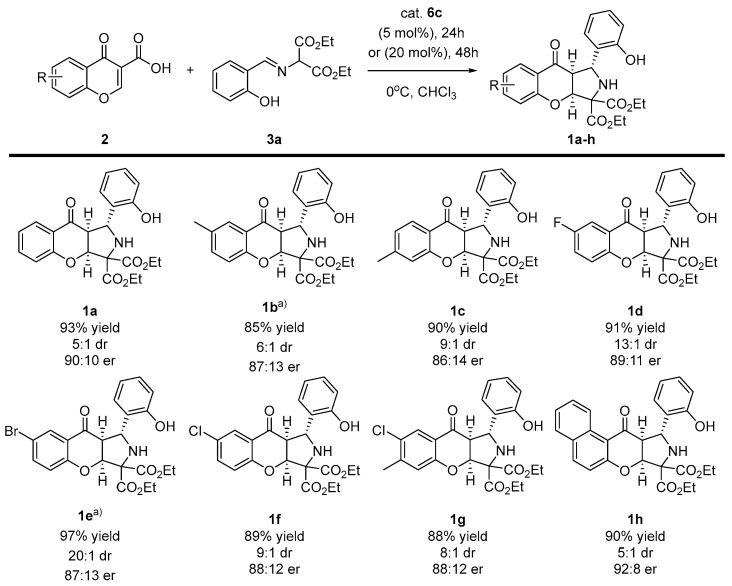
Enantioselective, decarboxylative (3+2)-cycloaddition of chromone-3-carboxylic acids **2** and diethyl iminomalonates **3a**—scope of chromone-3-carboxylic acid **2a-h**. ^a)^ Reactions performed for 48 h using 20 mol% of catalyst **6c**.

**Scheme 4:**
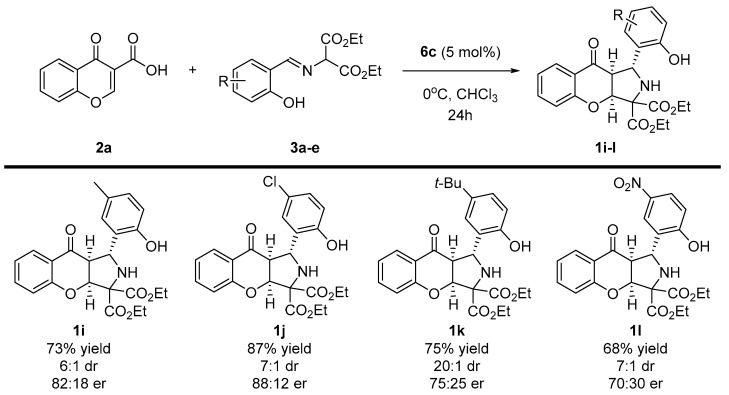
Enantioselective, decarboxylative (3+2)-cycloaddition of chromone-3-carboxylic acids **2a** and diethyl iminomalonates **3** to **1i-l**—scope diethyl iminomalonates **3a-e**.

Having accomplished the optimization studies, the scope of the methodology with regard to both reaction partners was studied (Scheme 3 and Scheme 4). Initially, various chromone-3-carboxylic acids **2** were reacted with the imine **3a** under optimized reaction conditions (Scheme 3). In some of the cases (Scheme 3, products **1b**,**f**) a longer reaction time and higher amounts of catalyst were required in order to achieve full conversion. To our delight, the decarboxylative (3+2)-cycloaddition proceeded efficiently, providing chromenopyrroles **1a-h** in good to high yields. Moreover, the diastereoselectivity of the developed reaction increased in all of the cases. In terms of enantioselectivity, the cycloaddition was found to be unbiased towards the electronic properties of substituents on the aromatic ring in acids **2a-h,** and it remained at a similar level to the model reaction. 

In the second part of the scope studies, the possibility of employing various diethyl iminomalonates **3a-e** in the devised strategy was tested (Scheme 4). It turned out that the application of imines **3a-e** had significant influences on both the yield and the enantioselectivity of the methodology. The reaction efficiency decreased compared to that shown in the first part of the optimization studies, and instead of obtaining the product quantitatively, yields in the order of 70–80% were obtained. Moreover, when the branching of the alkyl chain in **3d** was introduced, enantioselectivity lowered to 75:25 er. Gratifyingly, strongly electron-withdrawing substituent (NO_2_) **3e** was tolerated in this reaction, and the desired chromenopyrrole **1l** was afforded with good yield. The diastereoselectivity of the methodology remained at a good level; in addition for the reaction with the *tert*-butyl substituent, it was as high as 20:1 dr.

The possibility to replace the diethyl iminomalonates **3** scaffold with imines **4** bearing γ-lactone rings was also evaluated, and studies were initiated with the goal of finding optimal reaction conditions. In the first step, catalyst screening was performed, and gratifyingly, it was found that cinchona alkaloids **6a** promote cycloaddition (Table 2, entries 1–5). The reaction was terminated within 24 h, and chromenopyrrole **1** was obtained as a mixture of two diastereoisomers which differed in configuration on C-3 stereogenic centers with yields within the range of 70–90%. In particular, the application of bifunctional catalysts **6b-e**, bearing either a thiourea or squaramide moiety, led to a significant increase in the reaction enantioselectivity; however, its diastereoselectivity remained low. Among all catalysts tested, derivative **6c** proved optimal. With the best catalyst identified, solvent screening was initiated (Table 2, entries 7–10); however, none of the tested solvents were found to improve the reaction outcome, and therefore, further optimization was carried out using CH_2_Cl_2_ (Table 2, entry 5). In order to obtain better diastereoselectivity, the temperature was lowered to 0 °C (Table 2, entry 11). Carrying out the reaction at −20 °C did not provide any product. In the next part of the optimization studies, the influence of the catalyst amount and the concentration of the reaction were evaluated (Table 2, entries 13–16), leading to the identification of the optimal reaction parameters (Table 2, entry 13). Finally, the reaction time was extended from 24 to 48 h, which increased the reaction yield from 71 to 87%.

**Scheme 5:**
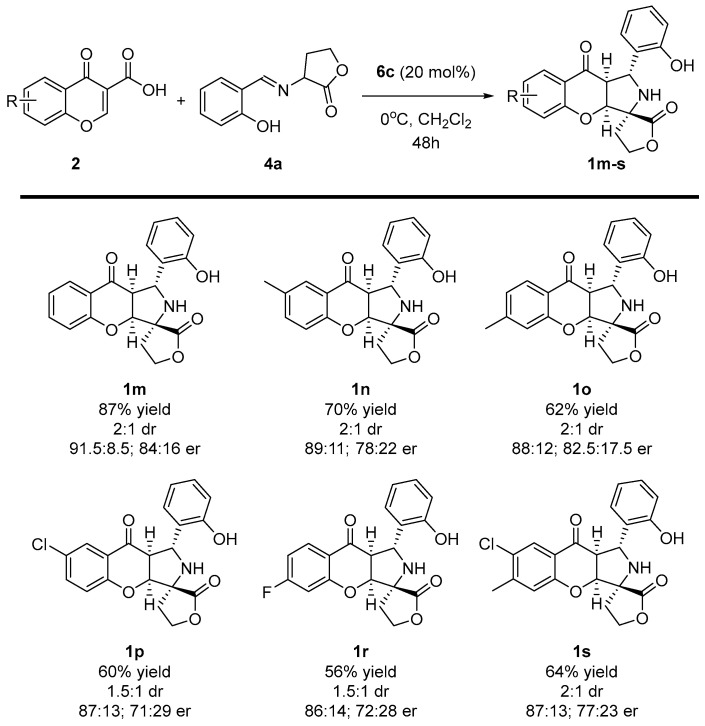
Enantioselective cycloaddition of chromone-3-carboxylic acids **2** and iminodihydrofuran-2-one **4a**—scope of chromone-3-carboxylic acids **2** to **1m-s**.

**Scheme 6:**
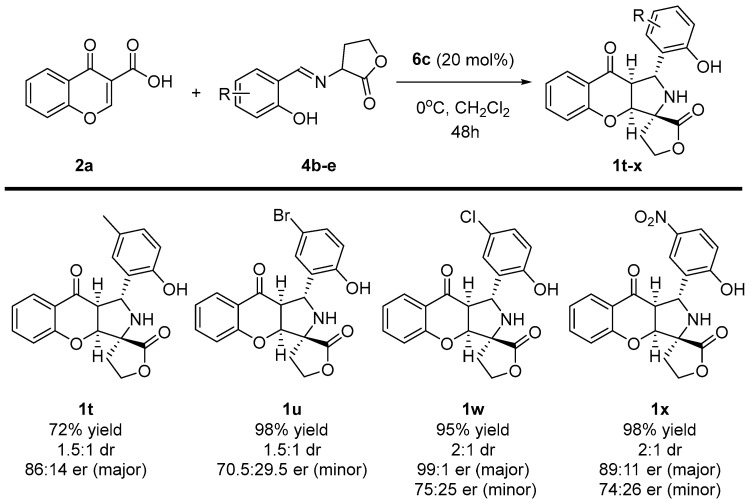
Enantioselective cycloaddition of chromone-3-carboxylic acids **2a** and iminodihydrofuran-2-one **4b-e**—scope of iminodihydrofuran-2-one **4** to **1t-x**.

Having established the best reaction conditions, the scope of the methodology was studied (Scheme 5 and Scheme 6). At the beginning, various chromone-3-carboxylic acids **2** containing either electron-withdrawing or donating substituents on the aromatic ring were tested in the reaction (Scheme 5). It was found that the enantioselectivity of the cascade remained at a similar level as for the model reaction; however, its efficiency decreased. Target products **1m-s** were obtained in yields within the range of 56–70% as a mixture of two diastereoisomers which differed in their configuration on the C-3 stereogenic center (only the main isomer was presented on Scheme 5 and Scheme 6). In this section, the studies also indicated that the position of the substituent in chromone-3-carboxylic acids **2** had no pronounced influence on the stereochemical reaction outcome, and the introduction of two substituents on the aromatic ring was also possible (Scheme 5, product **1s**). 

Importantly, the incorporation of different functional groups on the aromatic ring of iminodihydrofuran-2-ones **3** was also performed (Scheme 6). To our delight, apart from the example with methyl group **4b**, the annulative strategy took place with excellent yields. Moreover, target products **1t-x** were afforded with no significant influences either on the diastereoselectivity or the enantioselectivity of the reaction. In all cases, the desired chromenopyrroles **1t-x** were obtained as two diastereoisomers at a ratio of around 2:1. For the major diastereoisomer, the enantiomeric ratio was kept around 90:10 er, yet for the minor diastereoisomer, the enantiomeric excess remained at an average level.

**Scheme 7:**
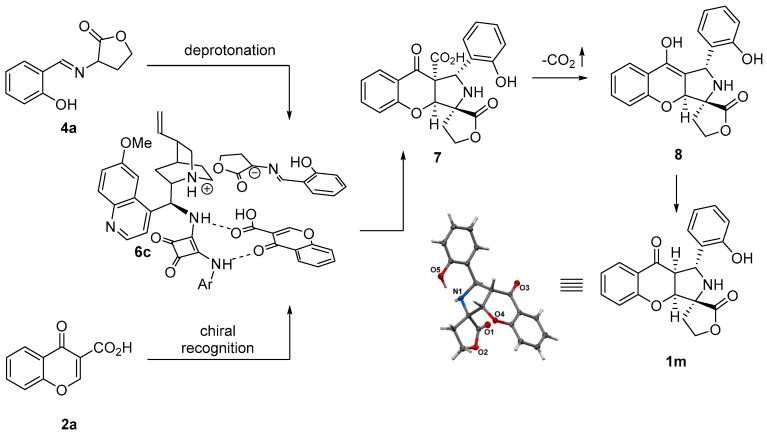
Mechanism of decarboxylative (3+2)-cycloaddition.

The absolute configurations of the major stereoisomer of chromone **1m** were unambiguously assigned by single-crystal X-ray analysis (Scheme 7) [45]. The absolute configurations of the remaining polycyclic products **1a-x** were assigned by analogy. Given these configurational assignments, the reaction mechanism explaining the observed stereochemistry of the products was proposed (Scheme 7). The reaction was initiated through the deprotonation of **4a** by the Brønsted base catalyst **6c** to give the corresponding azomethine ylide that participated in a (3+2)-cycloaddition. Importantly, it was postulated that, in this reaction, **6c** acted as a bifunctional catalyst. Firstly, a Brønsted base moiety in **6c** deprotonated the starting imine **4a** to form the corresponding ion pair. Secondly, the H-bonding unit of **6c** recognized the chromone-3-carboxylic acid **2a**. The subsequent cycloaddition of ylide with chromone-3-carboxylic acid yielded **1m**. The decarboxylation of **7** is the key step of the reaction, allowing for the removal of the activating group. The protonation of the enolate **8** thus obtained yielded the desired chromanones **1m**.

**Scheme 8:**
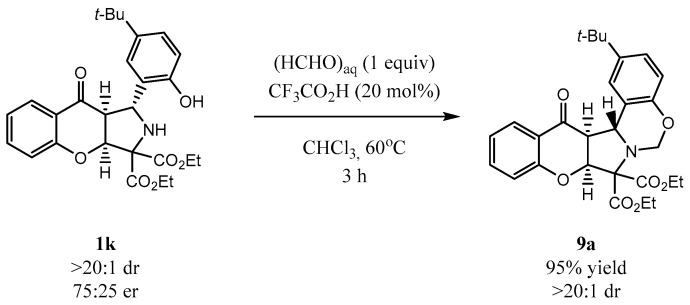
Synthesis of oxazine **9a** from **1k**.

To demonstrate the usefulness of cycloadducts **1** the annulation of **1k** with formaldehyde was attempted (Scheme 8). It was found that under acidic conditions in the presence of an aq. solution of formaldehyde, product **9a** bearing an oxazine moiety was obtained in 95% yield. Notably, the reaction proceeded with full preservation of the stereochemical information introduced at the decarboxylative cycloaddition step, as **9a** was obtained as a single diastereoisomer. 

## 3. Materials and Methods 

### 3.1. General Methods 

NMR spectra were acquired on a Bruker Ultra Shield 700 instrument (Bruker Corporation, Billerica, MA, USA), running at 700 MHz for ^1^H and 176 MHz for ^13^C, respectively. Chemical shifts (δ) were reported in ppm relative to residual solvent signals (CDCl_3_: 7.26 ppm for ^1^H NMR and 77.16 ppm for ^13^C NMR). Mass spectra were recorded on a Bruker Maxis Impact spectrometer using electrospray (ES+) ionization (referenced to the mass of the charged species). Analytical thin layer chromatography (TLC) was performed using pre-coated aluminium-backed plates (Merck Kieselgel 60 F254) and visualized by ultraviolet irradiation. Unless otherwise noted, analytical-grade solvents and commercially available reagents were used without further purification. For flash chromatography (FC), silica gel (Silica gel 60, 230–400 mesh, Merck, Darmstadt, Germany) was used. The enantiomeric ratio (er) of the products were determined by chiral stationary phase HPLC by Ultra Performance Convergence Chromatography (UPCC), using Daicel Chiralpak IA, IB, IC, and IG columns as chiral stationary phases. Diethyl iminomalonates **3** and iminodihydrofuran-2-one **4** were prepared from the corresponding 2-hydroxyaldehyde following the literature procedure [46]. Chromone-3-carboxylic acids **2** were prepared from the corresponding 2-hydroxyacetophenones following the literature procedure [47]. 

### 3.2. General Procedure 

#### 3.2.1. General Procedure for the Synthesis of Substituted (1R,3aS,9aS)-Diethyl 1-(2-Hydroxyphenyl)-9-oxo-1,2,9,9a-tetrahydrochromeno[2,3-c]pyrrole-3,3(3aH)-dicarboxylate **1a-l**

An ordinary screw-cap vial was charged with a magnetic stirring bar, the corresponding chromone-3-carboxylic acid **2** (0.1 mmol, 1 equiv), CHCl_3_ (0.2 mL), catalyst **6c** (0.005 mmol, 0.05 equiv), and the corresponding 2-hydroxyarylideneaminomalonates **3** (0.1 mmol, 1 equiv). The reaction mixture was stirred at 0 °C and monitored by ^1^H NMR spectroscopy. After the complete consumption of the chromone-3-carboxylic acid **2,** the mixture was directly subjected to FC on silica gel (hexane:ethyl acetate 5:1) to provide the desired products **1**.

(*1R,3aS,9aS*)-Diethyl 1-(2-hydroxyphenyl)-9-oxo-1,2,9,9a-tetrahydrochromeno[2,3-c]pyrrole-3,3(3aH)-dicarboxylate **1a**

Pure product was isolated via flash chromatography on silica gel (hexane/ethyl acetate 5:1) as yellow oil in 93% yield, 5:1 dr. ^1^H NMR (700 MHz, Chloroform-d) δ 10.20 (s, 1H), 7.91 (ddd, J = 7.9, 1.8, 0.4 Hz, 1H), 7.55–7.50 (m, 1H), 7.17 (ddd, J = 8.1, 7.3, 1.8 Hz, 1H), 7.10 (ddd, J = 7.9, 7.3, 1.0 Hz, 1H), 6.93 (ddd, J = 8.4, 1.0, 0.5 Hz, 1H), 6.85 (dd, J = 8.2, 1.2 Hz, 1H), 6.74 (td, J = 7.4, 1.2 Hz, 1H), 6.59 (ddt, J = 7.6, 1.8, 0.5 Hz, 1H), 5.67 (dd, J = 3.5, 0.8 Hz, 1H), 4.91 (d, J = 11.3 Hz, 1H), 4.50 (dqd, J = 10.7, 7.1, 1.4 Hz, 1H), 4.43–4.38 (m, 1H), 4.33 (qd, J = 7.1, 3.0 Hz, 2H), 3.34 (dd, J = 11.3, 3.5 Hz, 1H), 1.42 (t, J = 7.1 Hz, 3H), 1.34 (t, J = 7.1 Hz, 3H). ^13^C NMR (176 MHz, Chloroform-d) δ 188.04, 167.90, 166.75, 158.68, 157.29, 136.73, 129.72, 129.09, 127.31, 122.78, 120.44, 119.71, 119.37, 118.09, 117.84, 83.75, 76.16, 64.23, 63.42, 63.17, 55.29, 14.34, 14.07. HRMS calculated for [C_23_H_24_NO_7_^+^]: 426.1553, found: 426.1550. The er was determined by UPC2 using a chiral Chiralpack IB column gradient from 100% CO_2_ up to 40%; MeCN, 2.5 mL/min; detection wavelength = 245 nm; τmajor = 3.13 min, τminor = 2.96 min, (90:10 er). 〖[α]〗_D^20 = −44.9 (c = 1.0, CH_2_Cl_2_).

(*1R,3aS,9aS*)-Diethyl 1-(2-hydroxyphenyl)-7-methyl-9-oxo-1,2,9,9a-tetrahydrochromeno[2,3-c]pyrrole-3,3(3aH)-dicarboxylate **1b**

Pure product was isolated by flash chromatography on silica gel (hexane/ethyl acetate 5:1) as yellow oil solid in 85% yield, 6:1 dr. ^1^H NMR (700 MHz, Chloroform-d) δ 10.23 (s, 1H), 7.69 (dd, J = 2.3, 1.1 Hz, 1H), 7.33 (ddd, J = 8.4, 2.3, 0.8 Hz, 1H), 7.17 (ddd, J = 8.1, 7.4, 1.7 Hz, 1H), 6.84 (dd, J = 8.1, 1.2 Hz, 1H), 6.82 (d, J = 8.4 Hz, 1H), 6.73 (td, J = 7.4, 1.2 Hz, 1H), 6.59 (dd, J = 7.6, 1.6 Hz, 1H), 5.63 (d, J = 3.5 Hz, 1H), 4.89 (d, J = 11.3 Hz, 1H), 4.49 (dq, J = 10.7, 7.1 Hz, 1H), 4.44–4.37 (m, 1H), 4.33 (qd, J = 7.1, 2.7 Hz, 6H), 4.29 (s, 1H), 3.31 (dd, J = 11.3, 3.5 Hz, 1H), 2.33 (s, 3H), 1.42 (t, J = 7.1 Hz, 3H), 1.34 (t, J = 7.1 Hz, 3H). ^13^C NMR (176 MHz, Chloroform-d) δ 188.27, 167.94, 166.80, 157.30, 156.71, 137.76, 132.35, 129.68, 129.11, 126.86, 120.47, 119.33, 119.30, 117.87, 117.82, 83.69, 76.14, 64.25, 63.39, 63.13, 55.29, 20.58, 14.34, 14.06. HRMS calculated for [C_24_H_26_NO_7_^+^]: 440.1709, found: 440.1711. The er was determined by UPC2 using a chiral Chiralpack IA column gradient from 100% CO_2_ up to 40%; i-PrOH, 2.5 mL/min; detection wavelength = 245 nm; τmajor = 3.54 min, τminor = 3.65 min, (87:13 er). 〖[α]〗_D^20 = −49.9 (c = 1.0, CH_2_Cl_2_).

(*1R,3aS,9aS*)-Diethyl 1-(2-hydroxyphenyl)-6-methyl-9-oxo-1,2,9,9a-tetrahydrochromeno[2,3-c]pyrrole-3,3(3aH)-dicarboxylate **1c**

Pure product was isolated by flash chromatography on silica gel (hexane/ethyl acetate 5:1) as yellow solid m.p.: 201–203 °C in 90% yield, 9:1 dr. ^1^H NMR (700 MHz, Chloroform-d) δ 10.21 (s, 1H), 7.79 (d, J = 8.0 Hz, 1H), 7.17 (ddd, J = 8.4, 7.5, 1.7 Hz, 1H), 6.93–6.88 (m, 1H), 6.84 (dd, J = 8.4, 1.1 Hz, 1H), 6.76–6.70 (m, 2H), 6.59 (dd, J = 7.6, 1.7 Hz, 1H), 5.63 (d, J = 3.5 Hz, 1H), 4.88 (dd, J = 11.2, 6.6 Hz, 1H), 4.49 (dq, J = 10.6, 7.1 Hz, 1H), 4.42 (dq, J = 10.6, 7.1 Hz, 1H), 4.33 (qd, J = 7.1, 2.1 Hz, 2H), 4.27 (d, J = 6.9 Hz, 1H), 3.31 (dd, J = 11.2, 3.5 Hz, 1H), 2.38 (s, 3H), 1.43 (t, J = 7.1 Hz, 3H), 1.34 (t, J = 7.1 Hz, 3H). ^13^C NMR (176 MHz, Chloroform-d) δ 187.71, 167.93, 166.78, 158.69, 157.27, 148.46, 129.65, 129.07, 127.18, 124.09, 120.54, 119.34, 118.06, 117.80, 117.41, 83.71, 76.13, 64.30, 63.38, 63.12, 55.21, 22.10, 14.35, 14.06. HRMS calculated for [C_24_H_26_NO_7_^+^]: 440.1709, found: 440.1710. The er was determined by UPC2 using a chiral Chiralpack IA column gradient from 100% CO_2_ up to 40%; i-PrOH, 2.5 mL/min; detection wavelength = 245 nm; τmajor = 3.54 min, τminor = 3.41 min, (86:14 er). 〖[α]〗_D^20 = −38.6 (c = 1.0, CH_2_Cl_2_).

(*1R,3aS,9aS*)-Diethyl 7-fluoro-1-(2-hydroxyphenyl)-9-oxo-1,2,9,9a-tetrahydrochromeno[2,3-c]pyrrole-3,3(3aH)-dicarboxylate **1d**

Pure product was isolated by flash chromatography on silica gel (hexane/ethyl acetate 5:1) as yellow solid m.p.: 84–86 °C in 91% yield, 13:1 dr. ^1^H NMR (700 MHz, Chloroform-d) δ 10.11 (s, 1H), 7.55 (dd, J = 7.9, 3.2 Hz, 1H), 7.26–7.22 (m, 1H), 7.18 (td, J = 7.7, 1.6 Hz, 1H), 6.92 (dd, J = 9.0, 4.0 Hz, 1H), 6.84 (dd, J = 8.2, 1.1 Hz, 1H), 6.74 (td, J = 7.5, 1.1 Hz, 1H), 6.58 (dd, J = 7.5, 1.6 Hz, 1H), 5.65 (d, J = 3.5 Hz, 1H), 4.88 (dd, J = 11.3, 5.1 Hz, 1H), 4.52–4.44 (m, 1H), 4.44–4.36 (m, 1H), 4.32 (dtt, J = 9.6, 5.7, 3.0 Hz, 3H), 3.34 (dd, J = 11.3, 3.5 Hz, 1H), 1.41 (t, J = 7.1 Hz, 3H), 1.34 (t, J = 7.1 Hz, 3H). ^13^C NMR (176 MHz, Chloroform-d) δ 187.33, 167.22 (d, J= 211.77 Hz), 158.70, 157.29, 157.26, 154.81, 129.82, 129.06, 124.24 (d, J = 24.4 Hz), 120.31 (d, J = 6.6 Hz), 120.21, 119.89 (d, J = 7.4 Hz), 119.42, 117.88, 112.40 (d, J = 23.7 Hz), 84.00, 76.06, 64.08, 63.46, 63.22, 54.97, 14.33, 14.05. HRMS calculated for [C_23_H_23_FNO_7_^+^]: 444.1459, found: 444.1458. The er was determined by UPC2 using a chiral Chiralpack IA column gradient from 100% CO_2_ up to 40%; i-PrOH, 2.5 mL/min; detection wavelength = 245 nm; τmajor = 3.21 min, τminor = 3.34 min, (89:11 er). 〖[α]〗_D^20 = −46.0 (c = 1.0, CH_2_Cl_2_).

(*1R,3aS,9aS*)-Diethyl 7-bromo-1-(2-hydroxyphenyl)-9-oxo-1,2,9,9a-tetrahydrochromeno[2,3-c]pyrrole-3,3(3aH)-dicarboxylate **1e**

Pure product was isolated by flash chromatography on silica gel (hexane/ethyl acetate 5:1) as pale yellow solid m.p.: 98–101 °C in 97% yield, 20:1 dr. ^1^H NMR (700 MHz, Chloroform-d) δ 10.06 (s, 1H), 8.01 (d, J = 2.5 Hz, 1H), 7.61 (dd, J = 8.8, 2.5 Hz, 1H), 7.18 (ddd, J = 8.3, 7.5, 1.6 Hz, 1H), 6.86–6.81 (m, 2H), 6.74 (td, J = 7.5, 1.2 Hz, 1H), 6.58 (dd, J = 7.5, 1.6 Hz, 1H), 5.66 (d, J = 3.5 Hz, 1H), 4.86 (d, J = 11.3 Hz, 1H), 4.52–4.45 (m, 1H), 4.44–4.38 (m, 1H), 4.33 (qd, J = 7.1, 3.4 Hz, 2H), 4.30 (s, 1H), 3.35 (dd, J = 11.3, 3.5 Hz, 1H), 1.41 (t, J = 7.1 Hz, 3H), 1.34 (t, J = 7.1 Hz, 3H). ^13^C NMR (176 MHz, Chloroform-d) δ 186.86, 167.78, 166.57, 157.52, 157.23, 139.36, 129.87, 129.73, 129.05, 120.88, 120.16, 120.12, 119.46, 117.91, 115.54, 83.93, 76.10, 64.19, 63.51, 63.27, 54.95, 14.34, 14.06. HRMS calculated for [C_23_H_23_BrNO_7_^+^]: 504.0658, found: 504.0661. The er was determined by UPC2 using a chiral Chiralpack IA column gradient from 100% CO_2_ up to 40%; i-PrOH, 2.5 mL/min; detection wavelength = 245 nm; τmajor = 3.76 min, τminor = 3.92 min, (87:13 er). 〖[α]〗_D^20 = −40.7 (c = 1.0, CH_2_Cl_2_).

(*1R,3aS,9aS*)-Diethyl 7-chloro-1-(2-hydroxyphenyl)-9-oxo-1,2,9,9a-tetrahydrochromeno[2,3-c]pyrrole-3,3(3aH)-dicarboxylate **1f**

Pure product was isolated by flash chromatography on silica gel (hexane/ethyl acetate 5:1) as pale yellow solid m.p.: 118–121 °C in 89% yield 9:1 dr. ^1^H NMR (700 MHz, Chloroform-d) δ 10.07 (s, 1H), 7.86 (d, J = 2.7 Hz, 1H), 7.47 (dd, J = 8.8, 2.7 Hz, 1H), 7.21–7.16 (m, 1H), 6.90 (d, J = 8.8 Hz, 1H), 6.85 (d, J = 8.1 Hz, 1H), 6.74 (td, J = 7.6, 1.2 Hz, 1H), 6.58 (dd, J = 7.6, 1.6 Hz, 1H), 5.66 (d, J = 3.5 Hz, 1H), 4.87 (d, J = 10.5 Hz, 1H), 4.48 (dd, J = 10.7, 7.1 Hz, 1H), 4.41 (dd, J = 10.7, 7.1 Hz, 1H), 4.33 (qd, J = 7.1, 3.4 Hz, 2H), 4.29 (s, 1H), 3.35 (dd, J = 11.3, 3.5 Hz, 1H), 1.41 (t, J = 7.1 Hz, 3H), 1.34 (t, J = 7.1 Hz, 3H). ^13^C NMR (176 MHz, Chloroform-d) δ 186.99, 167.79, 166.59, 157.24, 157.05, 136.57, 129.88, 129.05, 128.43, 126.64, 120.45, 120.12, 119.85, 119.46, 117.91, 83.97, 76.10, 64.19, 63.51, 63.27, 54.99, 14.35, 14.07. HRMS calculated for [C_23_H_23_ClNO_7_^+^]: 474.1163, found: 474.1164. The er was determined by UPC2 using a chiral Chiralpack IA column gradient from 100% CO_2_ up to 40%; i-PrOH, 2.5 mL/min; detection wavelength = 245 nm; τmajor = 3.41 min, τminor = 3.58 min, (88:12 er). 〖[α]〗_D^20 = −54.0 (c = 1.0, CH_2_Cl_2_).

(*1R,3aS,9aS*)-Diethyl 7-chloro-1-(2-hydroxyphenyl)-6-methyl-9-oxo-1,2,9,9a-tetrahydrochromeno[2,3-c]pyrrole-3,3(3aH)-dicarboxylate **1g**

Pure product was isolated by flash chromatography on silica gel (hexane/ethyl acetate 5:1) as pale yellow oil in 88% yield, 8:1 dr. ^1^H NMR (700 MHz, Chloroform-d) δ 10.11 (s, 1H), 7.85 (s, 1H), 7.18 (ddd, J = 8.2, 7.3, 1.7 Hz, 1H), 6.84 (dd, J = 8.2, 1.2 Hz, 1H), 6.74 (td, J = 7.4, 1.2 Hz, 1H), 6.57 (dd, J = 7.7, 1.7 Hz, 1H), 5.63 (d, J = 3.5 Hz, 1H), 4.85 (d, J = 11.3 Hz, 1H), 4.48 (dq, J = 10.7, 7.1 Hz, 1H), 4.42 (dq, J = 10.7, 7.1 Hz, 1H), 4.33 (qd, J = 7.1, 2.4 Hz, 2H), 4.29 (s, 1H), 3.31 (dd, J = 11.3, 3.5 Hz, 1H), 2.40 (s, 3H), 1.42 (t, J = 7.1 Hz, 3H), 1.34 (t, J = 7.1 Hz, 3H). ^13^C NMR (176 MHz, Chloroform-d) δ 186.82, 167.83, 166.63, 157.23, 156.85, 146.03, 129.79, 129.05, 129.02, 126.94, 120.25, 120.15, 119.41, 118.60, 117.86, 83.90, 76.09, 64.26, 63.46, 63.21, 54.96, 21.01, 14.35, 14.05. HRMS calculated for [C_24_H_25_ClNO_7_^+^]: 474.1320, found: 474.1319. The er was determined by UPC2 using a chiral Chiralpack IG column gradient from 100% CO2 up to 40%; i-PrOH, 2.5 mL/min; detection wavelength = 245 nm; τmajor = 4.94 min, τminor = 4.54 min, (88:12 er). 〖[α]〗_D^20 = −37.0 (c = 1.0, CH_2_Cl_2_).

(*7aS,10R,10aS*)-Diethyl 10-(2-hydroxyphenyl)-11-oxo-9,10,10a,11-tetrahydrobenzo[5,6]chromeno[2,3-c]pyrrole-8,8(7aH)-dicarboxylate **1h**

Pure product was isolated by flash chromatography on silica gel (hexane/ethyl acetate 5:1) as pale yellow oil in 90% yield 5:1 dr. ^1^H NMR (700 MHz, Chloroform-d) δ 9.35 (ddd, J = 8.6, 1.2, 0.6 Hz, 1H), 7.98 (dt, J = 9.0, 0.6 Hz, 1H), 7.78 (ddt, J = 8.1, 1.3, 0.6 Hz, 1H), 7.64 (ddd, J = 8.6, 6.9, 1.4 Hz, 1H), 7.47 (ddd, J = 8.1, 6.9, 1.2 Hz, 1H), 7.18 (ddd, J = 8.2, 7.4, 1.7 Hz, 1H), 7.03 (d, J = 8.9 Hz, 1H), 6.87 (dd, J = 8.2, 1.2 Hz, 1H), 6.72 (td, J = 7.4, 1.2 Hz, 1H), 6.60–6.58 (m, 1H), 5.78 (dd, J = 3.9, 0.8 Hz, 1H), 4.97 (d, J = 11.1 Hz, 1H), 4.52 (dq, J = 10.6, 7.1 Hz, 1H), 4.44 (dq, J = 10.6, 7.1 Hz, 1H), 4.35 (qq, J = 7.1, 3.6 Hz, 2H), 3.43 (dd, J = 11.1, 3.6 Hz, 1H), 1.45 (t, J = 7.1 Hz, 3H), 1.36 (t, J = 7.1 Hz, 3H). ^13^C NMR (176 MHz, Chloroform-d) δ 189.78, 167.97, 165.87, 161.85, 157.22, 137.74, 130.44, 129.43, 129.30, 129.22, 128.96, 128.14, 125.53, 124.90, 119.20, 118.91, 118.39, 117.27, 111.79, 81.50, 78.05, 63.37, 63.30, 63.03, 53.40, 14.37, 14.21. HRMS calculated for [C_27_H_26_NO_7_^+^]: 475.1631, found: 475.1634. The er was determined by UPC2 using a chiral Chiralpack IA column gradient from 100% CO_2_ up to 40%; i-PrOH, 2.5 mL/min; detection wavelength = 245 nm; τmajor = 4.61 min, τminor = 4.44 min, (92:8 er). 〖[α]〗_D^20 = −48.6 (c = 1.0, CH_2_Cl_2_).

(*1R,3aS,9aS*)-Diethyl 1-(2-hydroxy-5-methylphenyl)-9-oxo-1,2,9,9a-tetrahydrochromeno[2,3-c]pyrrole-3,3(3aH)-dicarboxylate **1i**

Pure product was isolated by flash chromatography on silica gel (hexane/ethyl acetate 5:1) as pale yellow solid m.p.: 144–146 °C in 73% yield, 6:1 dr. ^1^H NMR (700 MHz, Chloroform-d) δ 9.89 (s, 1H), 7.92 (dd, J = 7.8, 1.8 Hz, 1H), 7.54 (ddd, J = 8.5, 7.2, 1.8 Hz, 1H), 7.13–7.09 (m, 1H), 6.97 (dd, J = 8.2, 2.1 Hz, 1H), 6.93 (dd, J = 8.5, 0.9 Hz, 1H), 6.75 (d, J = 8.2 Hz, 1H), 6.39 (d, J = 2.1 Hz, 1H), 5.66 (d, J = 3.5 Hz, 1H), 4.85 (d, J = 10.5 Hz, 1H), 4.52–4.47 (m, 1H), 4.43–4.37 (m, 1H), 4.33 (q, J = 7.1 Hz, 2H), 4.24 (s, 1H), 3.35 (dd, J = 10.5, 3.5 Hz, 1H), 2.17 (s, 3H), 1.42 (t, J = 7.1 Hz, 3H), 1.34 (t, J = 7.1 Hz, 3H). ^13^C NMR (176 MHz, Chloroform-d) δ 188.05, 167.97, 166.84, 158.71, 154.88, 136.68, 130.32, 129.49, 128.43, 127.33, 122.75, 120.18, 119.76, 118.09, 117.64, 83.84, 76.09, 64.20, 63.39, 63.14, 55.22, 20.59, 14.35, 14.07. HRMS calculated for [C_24_H_26_NO_7_^+^]: 440.1709, found: 440.1712. The er was determined by UPC2 using a chiral Chiralpack IB column gradient from 100% CO_2_ up to 40%; i-PrOH, 2.5 mL/min; detection wavelength = 245 nm; τmajor = 2.96 min, τminor = 2.70 min, (82:18 er). 〖[α]〗_D^20 = −44.4 (c = 1.0, CH_2_Cl_2_).

(*1R,3aS,9aS*)-Diethyl 1-(5-chloro-2-hydroxyphenyl)-9-oxo-1,2,9,9a-tetrahydrochromeno[2,3-c]pyrrole-3,3(3aH)-dicarboxylate **1j**

Pure product was isolated by flash chromatography on silica gel (hexane/ethyl acetate 5:1) as white solid m.p.: 167–169 °C in 87% yield, 7:1 dr. ^1^H NMR (700 MHz, Chloroform-d) δ 10.19 (s, 1H), 7.92 (dd, J = 7.8, 1.6 Hz, 1H), 7.56–7.51 (m, 1H), 7.14–7.09 (m, 2H), 6.92 (d, J = 8.3 Hz, 1H), 6.77 (d, J = 8.9 Hz, 1H), 6.60 (d, J = 2.5 Hz, 1H), 5.65 (d, J = 3.6 Hz, 1H), 4.84 (dd, J = 11.3, 4.7 Hz, 1H), 4.48 (dq, J = 10.4, 7.1 Hz, 1H), 4.39 (dq, J = 10.4 7.1 Hz, 1H), 4.32 (qd, J = 7.1, 3.9 Hz, 2H), 4.29 (s, 1H), 3.31 (dd, J = 11.3, 3.6 Hz, 1H), 1.41 (t, J = 7.1 Hz, 3H), 1.33 (t, J = 7.1 Hz, 3H). ^13^C NMR (176 MHz, Chloroform-d) δ 187.77, 167.75, 166.58, 158.66, 155.91, 136.88, 129.53, 128.62, 127.43, 123.99, 122.92, 122.19, 119.60, 119.17, 118.08, 83.62, 76.15, 63.51, 63.48, 63.24, 55.11, 14.32, 14.04. HRMS calculated for [C_23_H_23_ClNO_7_^+^]: 460.1163, found: 460.1162. The er was determined by UPC2 using a chiral Chiralpack IB column gradient from 100% CO_2_ up to 40%; i-PrOH, 2.5 mL/min; detection wavelength = 245 nm; τmajor = 2.94 min, τminor = 2.74 min, (88:12 er). 〖[α]〗_D^20 = −59.6 (c = 1.0, CH_2_Cl_2_).

(*1R,3aS,9aS*)-Diethyl 1-(5-(tert-butyl)-2-hydroxyphenyl)-9-oxo-1,2,9,9a-tetrahydrochromeno[2,3-c]pyrrole-3,3(3aH)-dicarboxylate **1k**

Pure product was isolated by flash chromatography on silica gel (hexane/ethyl acetate 5:1) as pale yellow oil in 75% yield, 20:1 dr. ^1^H NMR (700 MHz, Chloroform-d) δ 10.04 (s, 1H), 7.92 (dd, J = 7.8, 1.7 Hz, 1H), 7.54 (ddd, J = 8.7, 7.2, 1.7 Hz, 1H), 7.18 (dd, J = 8.5, 2.5 Hz, 1H), 7.14–7.08 (m, 1H), 6.93 (dd, J = 8.3, 0.9 Hz, 1H), 6.77 (d, J = 8.5 Hz, 1H), 6.54 (d, J = 2.5 Hz, 1H), 5.67 (d, J = 3.6 Hz, 1H), 4.92 (d, J = 11.3 Hz, 1H), 4.50 (dq, J = 10.6, 7.1 Hz, 1H), 4.41 (dd, J = 10.6, 7.1 Hz, 1H), 4.32 (q, J = 7.1 Hz, 2H), 4.28 (s, 1H), 3.32 (dd, J = 11.3, 3.6 Hz, 1H), 1.42 (t, J = 7.1 Hz, 3H), 1.34 (t, J = 7.1 Hz, 3H), 1.21 (s, 9H). ^13^C NMR (176 MHz, Chloroform-d) δ 187.92, 167.93, 166.79, 158.64, 154.75, 141.73, 136.60, 127.09, 126.32, 126.07, 122.78, 119.79, 119.58, 118.10, 117.20, 83.81, 76.30, 64.68, 63.39, 63.12, 55.53, 33.98, 31.50, 14.34, 14.07. HRMS calculated for [C_27_H_32_NO_7_^+^]: 482.2179, found: 482.2181. The er was determined by UPC2 using a chiral Chiralpack IG column gradient from 100% CO_2_ up to 40%; i-PrOH, 2.5 mL/min; detection wavelength = 245 nm; τmajor = 3.94 min, τminor = 4.14 min, (75:25 er). [α]^20^ = −43.4 (c = 1.0, CH_2_Cl_2_).

(*1R,3aS,9aS*)-Diethyl 1-(2-hydroxy-5-nitrophenyl)-9-oxo-1,2,9,9a-tetrahydrochromeno[2,3-c]pyrrole-3,3(3aH)-dicarboxylate **1l**

Pure product was isolated by flash chromatography on silica gel (hexane/ethyl acetate 5:1) as pale yellow solid m.p.: 152–154 °C in 68% yield, 7:1 dr. ^1^H NMR (700 MHz, Chloroform-d) δ 11.44 (s, 1H), 8.09 (dd, J = 9.0, 2.7 Hz, 1H), 7.93 (dd, J = 7.8, 1.7 Hz, 1H), 7.60 (d, J = 2.7 Hz, 1H), 7.57 (ddd, J = 8.6, 7.2, 1.7 Hz, 1H), 7.17–7.14 (m, 1H), 6.95 (dd, J = 8.6, 0.9 Hz, 1H), 6.90 (d, J = 9.0 Hz, 1H), 5.67 (d, J = 3.6 Hz, 1H), 4.98 (dd, J = 11.2, 6.1 Hz, 1H), 4.51 (dq, J = 10.6, 7.1 Hz, 1H), 4.46–4.39 (m, 2H), 4.38–4.30 (m, 2H), 3.29 (dd, J = 11.2, 3.6 Hz, 1H), 1.43 (t, J = 7.1 Hz, 3H), 1.35 (t, J = 7.1 Hz, 3H). ^13^C NMR (176 MHz, Chloroform-d) δ 187.50, 167.48, 166.23, 163.51, 158.65, 140.32, 137.18, 127.59, 125.84, 125.36, 123.24, 120.94, 119.51, 118.28, 118.12, 83.41, 76.30, 63.75, 63.47, 63.26, 55.25, 14.32, 14.03. HRMS calculated for [C_23_H_23_N_2_O_9_^+^]: 471.1402, found: 471.1403. The er was determined by UPC2 using a chiral Chiralpack IA column gradient from 100% CO_2_ up to 40%; i-PrOH, 2.5 mL/min; detection wavelength = 245 nm; τmajor = 3.53 min, τminor = 3.64 min, (70:30 er). 〖[α]〗_D^20 = −68.2 (c = 1.0, CH_2_Cl_2_).

#### 3.2.2. General Procedure for the Synthesis of Substituted 1-(2-Hydroxyphenyl)-3a,4′,5′,9a-tetrahydro-1H,2′H-spiro[chromeno[2,3-c]pyrrole-3,3′-furan]-2′,9(2H)-dione **1m-x**

An ordinary screw-cap vial was charged with a magnetic stirring bar, the corresponding chromone-3-carboxylic acid **2** (0.1 mmol, 1 equiv), CH_2_Cl_2_ (0.4 mL), catalyst **6c** (0.02 mmol, 0.02 equiv), and the corresponding 2-hydroxyarylideneaminolactones **4** (0.1 mmol, 1 equiv). The reaction mixture was stirred at 0 °C and monitored by ^1^H NMR spectroscopy. After the complete consumption of the chromone-3-carboxylic acid **2,** the mixture was directly subjected to FC on silica gel (CH_2_Cl_2_:acetone 100:1) to provide the desired products **1m-x**.

(*1R,3R,3aS,9aS*)-1-(2-Hydroxyphenyl)-3a,4′,5′,9a-tetrahydro-1H,2′H-spiro[chromeno[2,3-c]pyrrole-3,3′-furan]-2′,9(2H)-dione **1m**

Pure product was isolated by flash chromatography on silica gel (CH_2_Cl_2_/acetone 100:1) as white solid m.p.: 201–203 °C in 58% yield, 2:1 dr. ^1^H NMR (700 MHz, Chloroform-d) δ 7.89 (dd, J = 7.8, 1.7 Hz, 1H), 7.54 (ddd, J = 8.4, 7.2, 1.7 Hz, 1H), 7.21 (ddd, J = 8.1, 7.4, 1.7 Hz, 1H), 7.09 (ddd, J = 8.1, 7.2, 1.0 Hz, 1H), 7.01 (dd, J = 8.4, 1.0 Hz, 1H), 6.87 (ddd, J = 8.1, 4.3, 1.5 Hz, 2H), 6.81 (td, J = 7.4, 1.2 Hz, 1H), 5.15 (d, J = 9.8 Hz, 1H), 5.10 (dd, J = 5.7, 0.7 Hz, 1H), 4.53 (ddd, J = 9.6, 8.5, 1.3 Hz, 1H), 4.44 (ddd, J = 11.1, 9.6, 5.7 Hz, 1H), 3.34 (dd, J = 9.8, 5.6 Hz, 1H), 2.60 (ddd, J = 13.0, 11.1, 8.5 Hz, 1H), 2.53 (ddd, J = 13.0, 5.7, 1.3 Hz, 1H). ^13^C NMR (176 MHz, Chloroform-d) δ 188.37, 174.69, 158.58, 157.05, 137.05, 129.80, 128.98, 127.15, 122.80, 121.80, 119.83, 119.40, 118.24, 117.77, 83.64, 69.40, 65.19, 63.91, 53.48, 36.37. HRMS calculated for [C_20_H_18_NO_5_^+^]: 352.1185, found: 352.1185. The er was determined by UPC2 using a chiral Chiralpack IB column gradient from 100% CO_2_ up to 40%; MeCN, 2.5 mL/min; detection wavelength = 245 nm; τmajor = 3.20 min, τminor = 3.05 min, (91.5:8.5 er).

(*1R,3S,3aS,9aS*)-1-(2-Hydroxyphenyl)-3a,4′,5′,9a-tetrahydro-1H,2′H-spiro[chromeno[2,3-c]pyrrole-3,3′-furan]-2′,9(2H)-dione **1m′**

Pure product was isolated by flash chromatography on silica gel (CH_2_Cl_2_/acetone 100:1) as white solid m.p.: 188–190 °C in 29% yield, 2:1 dr. ^1^H NMR (700 MHz, Chloroform-d) δ 7.90 (ddd, J = 7.8, 1.8, 0.4 Hz, 1H), 7.57 (ddd, J = 8.4, 7.2, 1.8 Hz, 1H), 7.20 (ddd, J = 8.2, 7.2, 1.8 Hz, 1H), 7.12 (ddd, J = 7.8, 7.2, 1.0 Hz, 1H), 7.05 (ddd, J = 8.2, 1.0, 0.4 Hz, 1H), 6.91 (dd, J = 8.1, 1.2 Hz, 1H), 6.80–6.78 (m, 1H), 6.77–6.74 (m, 1H), 5.04 (dd, J = 4.6, 0.7 Hz, 1H), 4.88 (d, J = 10.8 Hz, 1H), 4.53–4.49 (m, 1H), 4.43 (td, J = 9.4, 6.4 Hz, 1H), 3.64 (dd, J = 10.8, 4.6 Hz, 1H), 3.21 (ddd, J = 13.3, 6.4, 3.1 Hz, 1H), 2.43–2.37 (m, 1H). ^13^C NMR (176 MHz, Chloroform-d) δ 188.34, 177.30, 158.46, 156.92, 136.88, 129.97, 129.28, 127.52, 122.98, 121.02, 119.91, 119.47, 117.96, 117.95, 81.65, 69.06, 65.57, 63.70, 53.48, 30.57. HRMS calculated for [C_20_H_18_NO_5_^+^]: 352.1185, found: 352.1184. The er was determined by UPC2 using a chiral Chiralpack IB column gradient from 100% CO_2_ up to 40%; i-PrOH, 2.5 mL/min; detection wavelength = 245 nm; τmajor = 4.91 min, τminor = 5.19 min, (84:16 er).

(*1R,3R,3aS,9aS*)-1-(2-Hydroxyphenyl)-7-methyl-3a,4′,5′,9a-tetrahydro-1H,2′H-spiro[chromeno[2,3-c]pyrrole-3,3′-furan]-2′,9(2H)-dione **1n**

Pure product was isolated by flash chromatography on silica gel (CH_2_Cl_2_/acetone 100:1) as pale yellow oil in 47% yield, 2:1 dr. ^1^H NMR (700 MHz, Chloroform-d) δ 10.27 (s, 1H), 7.67 (d, J = 1.2 Hz, 1H), 7.35 (ddd, J = 8.4, 2.4, 0.7 Hz, 1H), 7.20 (ddd, J = 8.0, 7.4, 1.7 Hz, 1H), 6.91 (d, J = 8.4 Hz, 1H), 6.87 (dt, J = 7.7, 1.7 Hz, 2H), 6.80 (td, J = 7.4, 1.2 Hz, 1H), 5.13 (d, J = 9.9 Hz, 1H), 5.06 (dd, J = 5.4, 0.7 Hz, 1H), 4.52 (ddd, J = 9.6, 8.6, 1.2 Hz, 1H), 4.43 (ddd, J = 11.2, 9.6, 5.6 Hz, 1H), 3.45 (s, 1H), 3.31 (dd, J = 9.9, 5.4 Hz, 1H), 2.59 (ddd, J = 13.1, 11.2, 8.6 Hz, 1H), 2.52 (ddd, J = 13.1, 5.6, 1.2 Hz, 1H), 2.32 (s, 3H). ^13^C NMR (176 MHz, Chloroform-d) δ 188.60, 174.78, 157.05, 156.63, 138.11, 132.36, 129.74, 128.99, 126.66, 121.86, 119.77, 119.00, 118.02, 117.72, 83.62, 69.36, 65.19, 63.91, 53.51, 36.40, 20.57. HRMS calculated for [C_21_H_20_NO_5_^+^]: 366.1297, found: 366.1298. The er was determined by UPC2 using a chiral Chiralpack IB column gradient from 100% CO_2_ up to 40%; MeCN, 2.5 mL/min; detection wavelength = 245 nm; τmajor = 4.69 min, τminor = 4.55 min, (89:11 er).

(*1R,3S,3aS,9aS*)-1-(2-Hydroxyphenyl)-7-methyl-3a,4′,5′,9a-tetrah-ydro-1H,2′H-spiro[chromeno[2,3-c]pyrrole-3,3′-furan]-2′,9(2H)-di-one **1n′**

Pure product was isolated by flash chromatography on silica gel (CH_2_Cl_2_/acetone 100:1) as pale yellow oil in 23% yield, 2:1 dr. ^1^H NMR (700 MHz, Chloroform-d) δ 7.67 (d, J = 2.3 Hz, 1H), 7.37 (dd, J = 8.5, 2.3 Hz, 1H), 7.19 (ddd, J = 8.5, 7.1, 2.0 Hz, 1H), 6.94 (d, J = 8.4 Hz, 1H), 6.92–6.89 (m, 1H), 6.75 (dtd, J = 11.2, 7.6, 1.6 Hz, 2H), 4.99 (d, J = 4.5 Hz, 1H), 4.85 (d, J = 10.8 Hz, 1H), 4.50 (td, J = 8.8, 3.0 Hz, 1H), 4.41 (td, J = 9.3, 6.4 Hz, 1H), 3.59 (dd, J = 10.8, 4.5 Hz, 1H), 3.20 (ddd, J = 13.2, 6.4, 3.0 Hz, 1H), 2.38 (dt, J = 13.2, 8.9 Hz, 1H), 2.33 (d, J = 2.6 Hz, 3H). ^13^C NMR (176 MHz, Chloroform-d) δ 188.56, 177.40, 156.94, 156.49, 137.90, 132.58, 129.91, 129.31, 127.08, 121.07, 119.52, 119.41, 117.93, 117.73, 81.57, 69.03, 65.56, 63.73, 53.51, 30.52, 20.59. HRMS calculated for [C_21_H_20_NO_5_^+^]: 366.1297, found: 366.1294. The er was determined by UPC2 using a chiral Chiralpack IC column gradient from 100% CO_2_ up to 40%; MeCN, 2.5 mL/min; detection wavelength = 245 nm; τmajor = 6.81 min, τminor = 6.16 min, (78:22 er).

(*1R,3R,3aS,9aS*)-1-(2-Hydroxyphenyl)-6-methyl-3a,4′,5′,9a-tetrahydro-1H,2′H-spiro[chromeno[2,3-c]pyrrole-3,3′-furan]-2′,9(2H)-dione **1o**

Pure product was isolated by flash chromatography on silica gel (CH_2_Cl_2_/acetone 100:1) as pale yellow solid m.p.: 180–182 °C in 41% yield, 2:1 dr. ^1^H NMR (700 MHz, Chloroform-d) δ 10.26 (s, 1H), 7.78 (d, J = 8.0 Hz, 1H), 7.20 (ddd, J = 8.7, 7.4, 1.7 Hz, 1H), 6.90 (d, J = 8.0 Hz, 1H), 6.87 (m, 2H), 6.83 (s, 1H), 6.80 (td, J = 7.4, 1.2 Hz, 1H), 5.13 (d, J = 9.8 Hz, 1H), 5.06 (d, J = 5.5 Hz, 1H), 4.53 (t, J = 9.0 Hz, 1H), 4.43 (ddd, J = 11.1, 9.6, 5.5 Hz, 1H), 3.43 (s, 1H), 3.31 (dd, J = 9.8, 5.5 Hz, 1H), 2.59 (ddd, J = 13.0, 10.9, 8.3 Hz, 1H), 2.52 (dd, J = 13.0, 5.5 Hz, 1H), 2.37 (s, 3H). ^13^C NMR (176 MHz, Chloroform-d) δ 188.04, 174.78, 158.58, 157.02, 148.88, 129.72, 128.94, 127.00, 124.12, 121.95, 119.78, 118.19, 117.71, 117.08, 83.65, 69.37, 65.19, 63.93, 53.38, 36.35, 22.11. HRMS calculated for [C_21_H_20_NO_5_^+^]: 366.1297, found: 366.1295. The er was determined by UPC2 using a chiral Chiralpack IC column gradient from 100% CO_2_ up to 40%; MeCN, 2.5 mL/min; detection wavelength = 245 nm; τmajor = 5.87 min, τminor = 4.93 min, (88:12 er).

(*1R,3S,3aS,9aS*)-1-(2-Hydroxyphenyl)-6-methyl-3a,4′,5′,9a-tetrah-ydro-1H,2′H-spiro[chromeno[2,3-c]pyrrole-3,3′-furan]-2′,9(2H)-dione **1o′**

Pure product was isolated by flash chromatography on silica gel (CH_2_Cl_2_/acetone 100:1) as pale yellow solid m.p.: 135–137 °C in 21% yield, 2:1 dr. ^1^H NMR (700 MHz, Chloroform-d) δ 9.46 (s, 1H), 7.78 (dd, J = 8.1, 3.2 Hz, 1H), 7.19 (ddd, J = 8.8, 7.4, 1.8 Hz, 1H), 6.91 (ddd, J = 13.1, 8.1, 1.4 Hz, 2H), 6.86 (s, 1H), 6.80 (dd, J = 7.5, 1.8 Hz, 1H), 6.75 (td, J = 7.4, 1.4 Hz, 1H), 5.01 (dd, J = 4.7, 0.7 Hz, 1H), 4.85 (d, J = 10.7 Hz, 1H), 4.50 (ddd, J = 9.3, 8.3, 3.1 Hz, 1H), 4.41 (td, J = 9.3, 6.4 Hz, 1H), 3.59 (dd, J = 10.7, 4.7 Hz, 1H), 3.17 (ddd, J = 13.2, 6.4, 3.1 Hz, 1H), 2.40 (d, J = 0.7 Hz, 3H), 2.38–2.35 (m, 1H). ^13^C NMR (176 MHz, Chloroform-d) δ 188.54, 177.53, 158.65, 156.64, 148.40, 129.45, 129.34, 127.00, 123.82, 122.11, 119.27, 117.81, 117.39, 117.27, 81.84, 68.91, 65.76, 63.37, 53.18, 30.18, 21.97. HRMS calculated for [C_21_H_20_NO_5_^+^]: 366.1297, found: 366.1298. The er was determined by UPC2 using a chiral Chiralpack IC column gradient from 100% CO_2_ up to 40%; MeCN, 2.5 mL/min; detection wavelength = 245 nm; τmajor = 6.18 min, τminor = 5.06 min, (82.5:17.5 er).

(*1R,3R,3aS,9aS*)-7-Chloro-1-(2-hydroxyphenyl)-3a,4′,5′,9a-tetrahydro-1H,2′H-spiro[chromeno[2,3-c]pyrrole-3,3′-furan]-2′,9(2H)-dione **1p**

Pure product was isolated by flash chromatography on silica gel (CH_2_Cl_2_/acetone 100:1) as yellow solid m.p.: 164–166 °C in 36% yield, 1.5:1 dr. ^1^H NMR (700 MHz, Chloroform-d) δ 7.84 (dd, J = 2.7, 1.6 Hz, 1H), 7.48 (ddd, J = 8.9, 2.7, 0.6 Hz, 1H), 7.21 (ddd, J = 8.0, 7.3, 1.6 Hz, 1H), 6.98 (d, J = 8.9 Hz, 1H), 6.87 (d, J = 1.0 Hz, 1H), 6.86 (d, J = 1.2 Hz, 1H), 6.81 (td, J = 7.3, 1.2 Hz, 1H), 5.12 (d, J = 9.7 Hz, 1H), 5.10 (d, J = 5.7 Hz, 1H), 4.53 (ddd, J = 9.6, 8.4, 1.6 Hz, 1H), 4.43 (ddd, J = 10.9, 9.6, 5.8 Hz, 1H), 3.34 (dd, J = 9.7, 5.7 Hz, 1H), 2.62–2.56 (m, 1H), 2.54 (ddd, J = 13.1, 5.8, 1.6 Hz, 1H). ^13^C NMR (176 MHz, Chloroform-d) δ 187.38, 174.60, 156.98, 156.96, 136.85, 129.93, 128.98, 128.39, 126.43, 121.60, 120.11, 119.96, 119.92, 117.80, 83.73, 69.35, 65.22, 63.87, 53.06, 36.22. HRMS calculated for [C_20_H_17_ClNO_5_^+^]: 387.0688, found: 387.0686. The er was determined by UPC2 using a chiral Chiralpack IC column gradient from 100% CO_2_ up to 40%; MeCN, 2.5 mL/min; detection wavelength = 245 nm; τmajor = 5.26 min, τminor = 4.64 min, (87:13 er).

(*1R,3S,3aS,9aS*)-7-Chloro-1-(2-hydroxyphenyl)-3a,4′,5′,9a-tetrahydro-1H,2′H-spiro[chromeno[2,3-c]pyrrole-3,3′-furan]-2′,9(2H)-dione **1p′**

Pure product was isolated by flash chromatography on silica gel (CH_2_Cl_2_/acetone 100:1) as yellow solid m.p.: 155–157 °C in 24% yield, 1.5:1 dr. ^1^H NMR (700 MHz, Chloroform-d) δ 7.86 (dd, J = 2.7, 0.4 Hz, 1H), 7.51 (dd, J = 8.8, 2.7 Hz, 1H), 7.23–7.19 (m, 1H), 7.02 (dd, J = 8.8, 0.4 Hz, 1H), 6.91 (dd, J = 7.8, 0.9 Hz, 1H), 6.77 (dd, J = 1.5, 0.9 Hz, 1H), 6.76 (dd, J = 2.1, 0.8 Hz, 1H), 5.03 (d, J = 4.4 Hz, 1H), 4.85 (dd, J = 10.9, 2.5 Hz, 1H), 4.52 (ddd, J = 9.3, 8.3, 3.2 Hz, 1H), 4.43 (td, J = 9.3, 6.5 Hz, 1H), 3.66 (dd, J = 10.9, 4.4 Hz, 1H), 3.19 (ddd, J = 13.3, 6.5, 3.2 Hz, 1H), 2.41 (ddd, J = 13.3, 9.1, 8.3 Hz, 1H). ^13^C NMR (176 MHz, Chloroform-d) δ 187.25, 177.10, 156.84, 156.82, 136.71, 130.15, 129.29, 128.66, 126.86, 120.68, 120.64, 119.71, 119.58, 118.02, 81.95, 69.06, 65.55, 63.66, 53.25, 31.07. HRMS calculated for [C_20_H_17_ClNO_5_^+^]: 387.0688, found: 387.0691. The er was determined by UPC2 using a chiral Chiralpack IA column gradient from 100% CO_2_ up to 40%; i-PrOH, 2.5 mL/min; detection wavelength = 245 nm; τmajor = 5.39 min, τminor = 5.72 min, (71:29 er).

(*1R,3R,3aS,9aS*)-6-Fluoro-1-(2-hydroxyphenyl)-3a,4′,5′,9a-tetrahydro-1H,2′H-spiro[chromeno[2,3-c]pyrrole-3,3′-furan]-2′,9(2H)-dione **1r**

Pure product was isolated by flash chromatography on silica gel (CH_2_Cl_2_/acetone 100:1) as pale yellow oil in 34% yield, 1.5:1 dr. ^1^H NMR (700 MHz, Chloroform-d) δ 10.12 (s, 1H), 7.92 (dd, J = 8.8, 6.5 Hz, 1H), 7.21 (ddd, J = 8.1, 7.3, 1.7 Hz, 1H), 6.88 (ddd, J = 17.1, 7.9, 1.4 Hz, 2H), 6.84–6.78 (m, 1H), 6.71 (dd, J = 9.5, 2.3 Hz, 1H), 5.14 (d, J = 9.6 Hz, 1H), 5.12 (d, J = 5.8 Hz, 1H), 4.53 (ddd, J = 9.8, 8.4, 1.6 Hz, 1H), 4.44 (ddd, J = 10.8, 9.6, 5.8 Hz, 1H), 3.40 (s, 1H), 3.34 (dd, J = 9.6, 5.9 Hz, 1H), 2.60 (ddd, J = 13.1, 10.8, 8.4 Hz, 1H), 2.55 (ddd, J = 13.1, 5.9, 1.6 Hz, 1H). ^13^C NMR (176 MHz, Chloroform-d) δ 187.03, 174.60, 168.13, (d, J = 258.03 Hz), 160.24 (d, J = 13.9 Hz), 156.96, 129.89, 129.82 (d, J = 11.4 Hz), 128.90, 121.85, 119.90, 117.82, 116.27 (d, J = 2.78 Hz), 111.28 (d, J = 22.6 Hz), 105.20 (d, J = 24.9 Hz), 84.09, 69.32, 65.21, 63.81, 53.01, 36.20. HRMS calculated for [C_20_H_17_FNO_5_^+^]: 370.1091, found: 370.1088. The er was determined by UPC2 using a chiral Chiralpack IB column gradient from 100% CO_2_ up to 40%; MeCN, 2.5 mL/min; detection wavelength = 245 nm; τmajor = 4.59 min, τminor = 4.44 min, (86:14 er).

(*1R,3S,3aS,9aS*)-6-Fluoro-1-(2-hydroxyphenyl)-3a,4′,5′,9a-tetrahydro-1H,2′H-spiro[chromeno[2,3-c]pyrrole-3,3′-furan]-2′,9(2H)-dione **1r′**

Pure product was isolated by flash chromatography on silica gel (CH_2_Cl_2_/acetone 100:1) as pale yellow oil in 22% yield, 1.5:1 dr. ^1^H NMR (700 MHz, Chloroform-d) δ 7.54 (ddd, J = 8.0, 3.2, 1.4 Hz, 1H), 7.29 (ddd, J = 9.0, 7.5, 3.2 Hz, 1H), 7.21–7.17 (m, 1H), 7.05 (dd, J = 9.0, 4.1 Hz, 1H), 6.89 (d, J = 8.0 Hz, 1H), 6.76–6.74 (m, 2H), 5.00 (d, J = 4.3 Hz, 1H), 4.84 (dd, J = 11.0, 3.0 Hz, 1H), 4.53–4.48 (m, 1H), 4.42 (td, J = 9.3, 6.4 Hz, 1H), 3.64 (dd, J = 11.0, 4.3 Hz, 1H), 3.19 (ddd, J = 13.3, 6.5, 3.0 Hz, 1H), 2.40 (dt, J = 13.3, 8.8 Hz, 1H). ^13^C NMR (176 MHz, Chloroform-d) δ 187.57, 177.27, 158.10 (d, J = 247.4 Hz), 156.89, 154.56, 130.06, 129.33, 124.42, 124.28, 120.7, 119.77 (d, J = 7.5 Hz), 119.50, 117.95, 112.64 (d, J = 23.6 Hz), 81.93, 69.01, 65.58, 63.60, 53.28, 30.48. HRMS calculated for [C_20_H_17_FNO_5_^+^]: 370.1091, found: 370.1090. The er was determined by UPC2 using a chiral Chiralpack IC column gradient from 100% CO_2_ up to 40%; i-PrOH, 2.5 mL/min; detection wavelength = 245 nm; τmajor = 6.30 min, τminor = 5.48 min, (72:28 er).

(*1R,3R,3aS,9aS*)-7-Chloro-1-(2-hydroxyphenyl)-6-methyl-3a,4′,5′,9a-tetrahydro-1H,2′H-spiro[chromeno[2,3-c]pyrrole-3,3′-furan]-2′,9(2H)-dione **1s**

Pure product was isolated by flash chromatography on silica gel (CH_2_Cl_2_/acetone 100:1) as pale yellow oil in 43% yield, 2:1 dr. ^1^H NMR (700 MHz, DMSO-d6) δ 9.90 (s, 1H), 7.76 (s, 1H), 7.45 (dd, J = 7.6, 1.7 Hz, 1H), 7.24 (td, J = 7.7, 1.7 Hz, 1H), 7.22 (d, J = 0.9 Hz, 1H), 6.93 (td, J = 7.4, 1.2 Hz, 1H), 6.89 (dd, J = 8.0, 1.2 Hz, 1H), 5.50 (d, J = 6.9 Hz, 1H), 5.16 (dd, J = 8.9, 5.3 Hz, 1H), 4.60–4.45 (m, 2H), 3.46 (s, 1H), 3.44 (dd, J = 8.9, 6.9 Hz, 1H), 2.74 (ddd, J = 12.9, 5.9, 2.5 Hz, 2H), 2.65 (m, 3H), 2.59–2.53 (m, 1H). ^13^C NMR (176 MHz, DMSO) δ 188.35, 175.49, 157.12, 155.51, 144.52, 128.37, 128.25, 126.27, 126.18, 125.21, 120.08, 118.83, 118.30, 115.40, 83.79, 69.22, 65.07, 59.17, 52.51, 34.84, 20.00. HRMS calculated for [C_21_H_19_ClNO_5_^+^]: 401.0844, found: 401.0842. The er was determined by UPC2 using a chiral Chiralpack IC column gradient from 100% CO_2_ up to 40%; MeCN, 2.5 mL/min; detection wavelength = 245 nm; τmajor = 5.78 min, τminor = 4.92 min, (87:13 er).

(*1R,3S,3aS,9aS*)-7-Chloro-1-(2-hydroxyphenyl)-6-methyl-3a,4′,5′,9a-tetrahydro-1H,2′H-spiro[chromeno[2,3-c]pyrrole-3,3′-furan]-2′,9(2H)-dione **1s′**

Pure product was isolated by flash chromatography on silica gel (CH_2_Cl_2_/acetone 100:1) as pale yellow oil in 21% yield, 2:1 dr. ^1^H NMR (700 MHz, Chloroform-d) δ 8.08 (d, J = 2.6 Hz, 1H), 7.84 (s, 1H), 7.20 (ddd, J = 8.2, 6.6, 2.3 Hz, 1H), 6.96 (s, 1H), 6.90 (dd, J = 8.2, 1.1 Hz, 1H), 6.78–6.74 (m, 1H), 5.01 (dd, J = 4.6, 0.7 Hz, 1H), 4.83 (d, J = 10.8 Hz, 1H), 4.67 (s, 1H), 4.50 (ddd, J = 9.3, 8.3, 3.2 Hz, 1H), 4.41 (td, J = 9.3, 6.5 Hz, 1H), 3.61 (dd, J = 10.8, 4.6 Hz, 1H), 3.16 (ddd, J = 13.2, 6.5, 3.2 Hz, 1H), 2.42 (s, 3H), 2.40–2.37 (m, 1H). ^13^C NMR (176 MHz, Chloroform-d) δ 187.12, 177.17, 156.84, 156.66, 146.20, 130.05, 129.26, 129.22, 127.15, 120.85, 120.04, 119.53, 118.81, 117.97, 81.86, 68.99, 65.54, 63.71, 53.18, 30.53, 29.85. HRMS calculated for [C_21_H_19_ClNO_5_^+^]: 401.0844, found: 401.0845. The er was determined by UPC2 using a chiral Chiralpack IB column gradient from 100% CO_2_ up to 40%; i-PrOH, 2.5 mL/min; detection wavelength = 245 nm; τmajor = 5.14 min, τminor = 5.52 min, (77:23 er).

(*1R,3R,3aS,9aS*)-1-(2-Hydroxy-5-methylphenyl)-3a,4′,5′,9a-tetrahydro-1H,2′H-spiro[chromeno[2,3-c]pyrrole-3,3′-furan]-2′,9(2H)-dione **1t**

Pure product was isolated by flash chromatography on silica gel (CH_2_Cl_2_/acetone 100:1) as yellow oil in 43% yield, 1.5:1 dr. A few drops of DMSO-d6 were added to increase the solubility. ^1^H NMR (700 MHz, Chloroform-d) δ 9.80 (s, 1H), 7.71 (dd, J = 7.8, 1.8 Hz, 1H), 7.38 (ddd, J = 8.5, 7.2, 1.8 Hz, 1H), 6.92 (td, J = 7.5, 1.0 Hz, 1H), 6.84 (dd, J = 8.5, 1.0 Hz, 1H), 6.82 (dd, J = 8.2, 2.2 Hz, 1H), 6.72 (d, J = 2.1 Hz, 1H), 6.59 (d, J = 8.2 Hz, 1H), 5.04 (d, J = 6.9 Hz, 1H), 4.98 (d, J = 8.8 Hz, 1H), 4.35 (ddd, J = 9.2, 8.2, 2.6 Hz, 1H), 4.31 (td, J = 9.6, 6.2 Hz, 1H), 3.59 (s, 1H), 3.22 (dd, J = 8.8, 6.9 Hz, 1H), 2.44–2.41 (m, 1H), 2.09 (s, 3H). ^13^C NMR (176 MHz, Chloroform-d) δ 188.89, 175.05, 158.45, 154.07, 136.35, 129.56, 129.09, 128.34, 126.53, 121.97, 119.20, 117.70, 116.77, 83.15, 69.02, 65.08, 62.65, 52.47, 35.26, 29.45, 20.33. HRMS calculated for [C_21_H_20_NO_5_^+^]: 366.1341, found: 366.1333. The er was determined by UPC2 using a chiral Chiralpack IB column gradient from 100% CO_2_ up to 40%; i-PrOH, 2.5 mL/min; detection wavelength = 245 nm; τmajor = 5.18 min, τminor = 4.87 min, (86:14 er).

(*1R,3S,3aS,9aS*)-1-(2-Hydroxy-5-methylphenyl)-3a,4′,5′,9a-tetrahydro-1H,2′H-spiro[chromeno[2,3-c]pyrrole-3,3′-furan]-2′,9(2H)-dione **1t′**

Pure product was isolated by flash chromatography on silica gel (CH_2_Cl_2_/acetone 100:1) as yellow oil in 29% yield, 1.5:1 dr. ^1^H NMR (700 MHz, Chloroform-d) δ 7.90 (dd, J = 7.8, 1.8 Hz, 1H), 7.57 (ddd, J = 8.7, 7.2, 1.8 Hz, 1H), 7.14–7.11 (m, 1H), 7.04 (d, J = 7.8 Hz, 1H), 6.99 (dd, J = 8.3, 2.2 Hz, 1H), 6.81 (dd, J = 8.2, 2.2 Hz, 1H), 6.61 (d, J = 2.2 Hz, 1H), 5.05 (d, J = 4.8 Hz, 1H), 4.82 (dd, J = 10.6, 7.1 Hz, 1H), 4.50 (td, J = 8.8, 3.2 Hz, 1H), 4.42 (td, J = 9.3, 6.5 Hz, 1H), 3.63 (dd, J = 10.6, 4.8 Hz, 1H), 3.17 (ddd, J = 13.2, 6.5, 3.2 Hz, 1H), 2.37 (dt, J = 13.2, 8.8 Hz, 1H), 2.18 (s, 3H). ^13^C NMR (176 MHz, Chloroform-d) δ 188.49, 177.36, 158.55, 154.45, 136.85, 130.53, 129.63, 128.59, 127.51, 122.90, 120.97, 117.92, 117.74, 81.79, 68.98, 65.59, 63.56, 53.34, 30.61, 29.85, 20.59. HRMS calculated for [C_21_H_20_NO_5_^+^]: 366.1341, found: 366.1338.

(*1R,3R,3aS,9aS*)-1-(5-Bromo-2-hydroxyphenyl)-3a,4′,5′,9a-tetrahydro-1H,2′H-spiro[chromeno[2,3-c]pyrrole-3,3′-furan]-2′,9(2H)-dione **1u**

Pure product was isolated by flash chromatography on silica gel (CH_2_Cl_2_/acetone 100:1) as white powder in 59% yield, 1.5:1 dr. A few drops of DMSO-d6 were added to increase the solubility. ^1^H NMR (700 MHz, Chloroform-d) δ 9.73 (s, 1H), 7.44 (dd, J = 7.9, 1.7 Hz, 1H), 7.15 (ddd, J = 8.4, 7.1, 1.7 Hz, 1H), 7.04 (dd, J = 2.5, 0.6 Hz, 1H), 6.84 (dd, J = 8.6, 2.5 Hz, 1H), 6.68 (ddd, J = 7.9, 7.1, 1.0 Hz, 1H), 6.59 (dd, J = 8.4, 1.0 Hz, 1H), 6.35 (d, J = 8.6 Hz, 1H), 4.85 (d, J = 7.3 Hz, 1H), 4.79 (d, J = 8.4 Hz, 1H), 4.14–4.05 (m, 2H), 3.52 (s, 1H), 2.93 (dd, J = 8.3, 7.5 Hz, 1H), 2.27 (ddd, J = 13.0, 6.0, 3.0 Hz, 1H), 2.23–2.15 (m, 2H). ^13^C NMR (176 MHz, Chloroform-d) δ 188.83, 174.83, 158.09, 154.65, 135.70, 130.67, 130.29, 127.06, 125.78, 121.14, 118.60, 117.72, 117.09, 110.28, 82.46, 68.52, 64.62, 59.65, 51.82, 34.32. HRMS calculated for [C_20_H_17_BrNO_5_^+^]: 430.0290, found: 430.0285.

(*1R,3S,3aS,9aS*)-1-(5-Bromo-2-hydroxyphenyl)-3a,4′,5′,9a-tetrahydro-1H,2′H-spiro[chromeno[2,3-c]pyrrole-3,3′-furan]-2′,9(2H)-dione **1u′**

Pure product was isolated by flash chromatography on silica gel (CH_2_Cl_2_/acetone 100:1) as white powder in 39% yield, 1.5:1 dr. ^1^H NMR (700 MHz, Chloroform-d) δ 9.53 (s, 1H), 7.91 (dt, J = 7.8, 1.9 Hz, 1H), 7.58 (ddd, J = 8.7, 7.2, 1.9 Hz, 1H), 7.27 (t, J = 2.8 Hz, 1H), 7.15–7.12 (m, 1H), 7.05 (dd, J = 8.3, 0.9 Hz, 1H), 6.92–6.89 (m, 1H), 6.78 (dd, J = 8.7, 5.9 Hz, 1H), 5.02 (dd, J = 4.6, 2.5 Hz, 1H), 4.80 (dd, J = 10.7, 2.5 Hz, 1H), 4.51 (td, J = 8.9, 2.9 Hz, 1H), 4.42 (td, J = 9.4, 6.4 Hz, 1H), 3.60 (ddd, J = 10.7, 4.6, 1.9 Hz, 1H), 3.20 (ddd, J = 13.3, 6.4, 2.9 Hz, 1H), 2.69 (s, 1H), 2.39 (dt, J = 13.3, 8.9 Hz, 1H). ^13^C NMR (176 MHz, Chloroform-d) δ 188.01, 177.36, 158.45, 156.08, 137.04, 132.69, 131.79, 127.62, 123.32, 123.14, 119.84, 117.95, 111.21, 81.47, 69.11, 65.62, 63.03, 53.26, 30.39, 29.85. HRMS calculated for [C_20_H_17_BrNO_5_+]: 430.0290, found: 430.0283. The er was determined by UPC2 using a chiral Chiralpack IC column gradient from 100% CO_2_ up to 40%; MeCN, 2.5 mL/min; detection wavelength = 245 nm; τmajor = 5.67 min, τminor = 4.98 min, (70.5:29.5 er).

(*1R,3R,3aS,9aS*)-1-(5-Chloro-2-hydroxyphenyl)-3a,4′,5′,9a-tetrahydro-1H,2′H-spiro[chromeno[2,3-c]pyrrole-3,3′-furan]-2′,9(2H)-dione **1w**

Pure product was isolated by flash chromatography on silica gel (CH_2_Cl_2_/acetone 100:1) as white powder in 63% yield, 2:1 dr. A few drops of DMSO-d6 were added to increase the solubility. ^1^H NMR (700 MHz, Chloroform-d) δ 10.17 (s, 1H), 7.89 (dd, J = 7.9, 1.8 Hz, 1H), 7.53 (ddd, J = 8.4, 7.2, 1.8 Hz, 1H), 7.14 (dd, J = 8.6, 2.6 Hz, 1H), 7.09 (ddd, J = 7.9, 7.2, 1.0 Hz, 1H), 7.02–6.96 (m, 2H), 6.80 (d, J = 8.6 Hz, 1H), 5.13 (d, J = 8.8 Hz, 1H), 5.10 (d, J = 6.6 Hz, 1H), 4.52–4.49 (m, 1H), 4.47–4.43 (m, 1H), 3.30 (dd, J = 8.8, 6.6 Hz, 1H), 3.28 (s, 1H), 2.60–2.54 (m, 2H). ^13^C NMR (176 MHz, Chloroform-d) δ 188.13, 174.17, 157.49, 153.34, 134.85, 126.73, 126.69, 126.65, 124.95, 121.84, 120.26, 117.97, 116.38, 116.12, 82.04, 67.92, 63.87, 58.14, 51.43, 33.79. HRMS calculated for [C_20_H_17_ClNO_5_^+^]: 387.0688, found: 387.0694. The er was determined by UPC2 using a chiral Chiralpack IC column gradient from 100% CO_2_ up to 40%; MeCN, 2.5 mL/min; detection wavelength = 245 nm; τmajor = 4.78 min, τminor = 4.50 min, (99:1 er).

(*1R,3S,3aS,9aS*)-1-(5-Chloro-2-hydroxyphenyl)-3a,4′,5′,9a-tetrahydro-1H,2′H-spiro[chromeno[2,3-c]pyrrole-3,3′-furan]-2′,9(2H)-dione **1w′**

Pure product was isolated by flash chromatography on silica gel (CH_2_Cl_2_/acetone 100:1) as white powder in 32% yield, 2:1 dr. A few drops of DMSO-d6 were added to increase the solubility. ^1^H NMR (700 MHz, Chloroform-d) δ 9.44 (s, 1H), 7.92 (dd, J = 7.8, 1.7 Hz, 1H), 7.58 (ddd, J = 8.7, 7.2, 1.7 Hz, 1H), 7.16–7.13 (m, 2H), 7.05 (dd, J = 8.4, 0.9 Hz, 1H), 6.85 (dd, J = 8.7, 1.9 Hz, 1H), 6.79 (d, J = 2.5 Hz, 1H), 5.04 (d, J = 4.6 Hz, 1H), 4.82 (d, J = 10.7 Hz, 1H), 4.54–4.50 (m, 1H), 4.43 (td, J = 9.4, 6.4 Hz, 1H), 3.63 (dd, J = 10.7, 4.6 Hz, 1H), 3.21 (ddd, J = 13.3, 6.4, 2.9 Hz, 1H), 2.65 (s, 1H), 2.39 (dt, J = 13.3, 8.9 Hz, 1H). ^13^C NMR (176 MHz, Chloroform-d) δ 188.71, 177.29, 158.59, 154.99, 136.49, 128.77, 128.70, 126.85, 124.70, 123.52, 122.23, 119.43, 118.01, 117.72, 81.98, 68.80, 65.69, 61.67, 53.35, 30.00, 29.46. HRMS calculated for [C_20_H_17_ClNO_5_^+^]: 387.0688, found: 387.0686. The er was determined by UPC2 using a chiral Chiralpack IC column gradient from 100% CO_2_ up to 40%; MeCN, 2.5 mL/min; detection wavelength = 245 nm; τmajor = 5.39 min, τminor = 4.81 min, (75:25 er).

(*1R,3R,3aS,9aS*)-1-(2-Hydroxy-5-nitrophenyl)-3a,4′,5′,9a-tetrahydro-1H,2′H-spiro[chromeno[2,3-c]pyrrole-3,3′-furan]-2′,9(2H)-dione **1x**

Pure product was isolated by flash chromatography on silica gel (CH_2_Cl_2_/acetone 100:1) as pale yellow oil in 65% yield, 2:1 dr. A few drops of DMSO-d6 were added to increase the solubility. ^1^H NMR (700 MHz, Chloroform-d) δ 11.30 (s, 1H), 8.13 (dd, J = 9.0, 2.8 Hz, 1H), 8.06 (dd, J = 2.8, 0.7 Hz, 1H), 7.93 (ddd, J = 8.0, 1.8, 0.5 Hz, 1H), 7.58 (ddd, J = 8.4, 7.2, 1.8 Hz, 1H), 7.13 (ddd, J = 8.0, 7.2, 1.0 Hz, 1H), 7.01 (ddd, J = 8.4, 1.0, 0.5 Hz, 1H), 6.96 (d, J = 9.0 Hz, 1H), 5.29 (d, J = 9.2 Hz, 1H), 5.12 (dd, J = 6.8, 0.6 Hz, 1H), 4.58–4.52 (m, 1H), 4.52–4.46 (m, 1H), 3.29 (dd, J = 8.5, 6.8 Hz, 1H), 3.28 (s, 1H), 2.68–2.59 (m, 2H). ^13^C NMR (176 MHz, Chloroform-d) δ 188.70, 174.77, 161.62, 158.14, 139.50, 135.84, 126.04, 125.79, 124.20, 123.91, 121.24, 118.54, 117.20, 115.81, 82.73, 77.16, 68.63, 64.60, 58.64, 52.30, 34.72. HRMS calculated for [C_20_H_17_N_2_O_7_^+^]: 397.0991, found: 397.0994. The er was determined by UPC2 using a chiral Chiralpack IB column gradient from 100% CO_2_ up to 40%; i-PrOH, 2.5 mL/min; detection wavelength = 245 nm; τmajor = 5.20 min, τminor = 5.38 min, (89:11 er).

(*1R,3S,3aS,9aS*)-1-(2-Hydroxy-5-nitrophenyl)-3a,4′,5′,9a-tetrahydro-1H,2′H-spiro[chromeno[2,3-c]pyrrole-3,3′-furan]-2′,9(2H)-dione **1x′**

Pure product was isolated by flash chromatography on silica gel (CH_2_Cl_2_/acetone 100:1) as pale yellow oil in 33% yield, 2:1 dr. ^1^H NMR (700 MHz, Chloroform-d) δ 10.70 (s, 1H), 8.09 (ddd, J = 9.0, 2.7, 1.7 Hz, 1H), 7.91 (dd, J = 7.8, 1.7 Hz, 1H), 7.69 (dd, J = 2.7, 0.6 Hz, 1H), 7.61 (ddd, J = 8.4, 7.2, 1.7 Hz, 1H), 7.18–7.15 (m, 1H), 7.07 (dd, J = 8.4, 1.0 Hz, 1H), 6.94 (dd, J = 9.0, 1.5 Hz, 1H), 5.02 (d, J = 4.1 Hz, 1H), 4.94 (d, J = 11.1 Hz, 1H), 4.56 (ddd, J = 9.4, 8.5, 2.5 Hz, 1H), 4.46 (td, J = 9.7, 6.3 Hz, 1H), 3.59 (ddd, J = 11.1, 4.1, 0.5 Hz, 1H), 3.31–3.27 (m, 1H), 2.46 (ddd, J = 13.3, 9.7, 8.5 Hz, 1H). ^13^C NMR (176 MHz, Chloroform-d) δ 187.58, 177.39, 163.16, 158.34, 140.30, 137.29, 127.73, 126.08, 125.80, 123.47, 121.32, 119.79, 118.52, 118.04, 81.11, 69.46, 65.71, 63.20, 53.46, 30.12. HRMS calculated for [C_20_H_17_N_2_O_7_^+^]: 397.0991, found: 397.0993. The er was determined by UPC2 using a chiral Chiralpack IB column gradient from 100% CO_2_ up to 40%; MeOH, 2.5 mL/min; detection wavelength = 245 nm; τmajor = 5.20 min, τminor = 5.38 min, (74:26 er).

#### 3.2.3. General Procedure for the synthesis of the Tetrahydro-spiro[benzo[e]chromeno[3′,2′:3,4]pyrrolo[1,2-c][1,3]oxazine **9a**

An ordinary screw-cap vial was charged with a magnetic stirring bar, the corresponding pyrrolidine **1k** (0.1 mmol, 1 equiv), 37% aq solution of formaldehyde (0.1 mmol, 1 equiv), trifluoroacetic acid (0.2 equiv), and CHCl_3_ (0.5 mL). The resulting mixture was stirred at 60 °C for 3 h. Subsequently, sat. aq NaHCO_3_ (1 mL) was added, phases were separated, and the aqueous layer was extracted with CH_2_Cl_2_ (2 × 2 mL). The combined organic layers were dried (anhyd. MgSO_4_). After filtration, volatiles were removed under reduced pressure, and the residue was directly subjected to FC on silica gel (eluent: hexane/EtOAc) to afford the desired product **9a**.

(*8aS,14aS,14bR*)-Diethyl 2-(*tert*-butyl)-14-oxo-8a,14,14a,14b-tetrahydro-benzo[e]chromeno[3′,2′:3,4]pyrrolo[1,2-c][1,3]oxazine-8,8(6*H*)-dicarboxylate **9a**

Pure product was isolated by flash chromatography on silica gel (hexane/ethyl acetate 15:1) as pale yellow oil in 95% yield. ^1^H NMR (700 MHz, Chloroform-d) δ 7.97 (ddd, J = 7.9, 1.8, 0.5 Hz, 1H), 7.60 (dd, J = 2.4, 0.9 Hz, 1H), 7.50 (ddd, J = 8.2, 7.2, 1.8 Hz, 1H), 7.13 (ddd, J = 8.5, 2.4, 0.7 Hz, 1H), 7.08 (ddd, J = 8.2, 7.2, 1.0 Hz, 1H), 6.91 (ddd, J = 8.5, 1.1, 0.5 Hz, 1H), 6.66 (d, J = 8.5 Hz, 1H), 5.38 (dt, J = 5.1, 0.6 Hz, 1H), 5.31 (dd, J = 11.4, 0.5 Hz, 1H), 5.14 (d, J = 7.4 Hz, 1H), 5.06 (dd, J = 11.4, 0.7 Hz, 1H), 4.49 (dq, J = 10.7, 7.1 Hz, 1H), 4.37 (dq, J = 10.7, 7.1 Hz, 1H), 4.15 (qd, J = 7.1, 2.2 Hz, 2H), 3.75 (dd, J = 7.4, 5.1 Hz, 1H). ^13^C NMR (176 MHz, Chloroform-d) δ 190.34, 168.58, 167.02, 160.20, 151.77, 144.93, 136.71, 127.41, 124.94, 124.61, 124.39, 122.51, 119.82, 118.37, 116.45, 83.94, 76.47, 62.94, 62.19, 59.95, 55.24, 34.56, 31.67 (3xC), 14.35, 13.59. HRMS calculated for [C_28_H_32_NO_7_^+^]: 494.2134, found: 494.2136. The er was determined by UPC2 using a chiral Chiralpack IA column gradient from 100% CO_2_ up to 40%; i-PrOH, 2.5 mL/min; detection wavelength = 245 nm; τmajor = 2.93 min, τminor = 3.30 min, (75:25 er).

## 4. Conclusions

In conclusion, a new decarboxylative (3+2)-cycloaddition of azomethine ylides **3** or **4** with chromone-3-carboxylic acids **2** was developed. The scope studies confirmed the high efficiency of the transformation with regard to both chromone-3-carboxylic acids and diethyl iminomalonates **3** or iminodihydrofuran-2-one **4,** providing access to a wide variety of interesting hybrid molecules bearing two important heterocyclic scaffolds: chromanone–pyrolidine ring systems.

## Figures and Tables

**Table 1:** Enantioselective (3+2)-cycloaddition of chromone-3-carboxylic acids **2** and diethyl iminomalonates **3**—optimization studies ^a^.

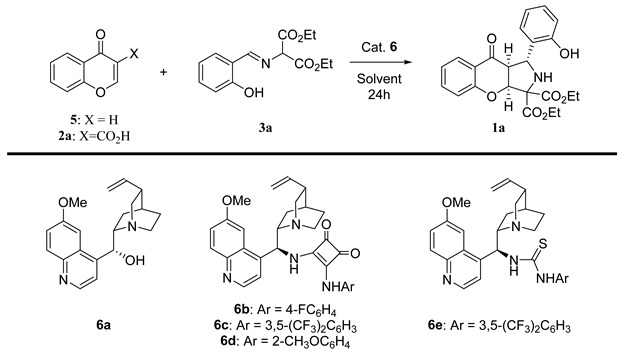							
**No.**	**Cat.**	**Solvent**	**X**	**Temp.**	**Yield (%)**	**dr**	**er**
1	**6a**	CH_2_Cl_2_	**5** (H)	r.t.	-	-	-
2	**6a**	CH_2_Cl_2_	**2a**(COOH)	r.t.	98%	>20:1	52:48
3	**6b**	CH_2_Cl_2_	**2a**(COOH)	r.t.	97%	8:1	75:25
4	**6c**	CH_2_Cl_2_	**2a**(COOH)	r.t.	98%	4:1	80:20
5	**6d**	CH_2_Cl_2_	**2a**(COOH)	r.t.	97%	4:1	71:29
6	**6e**	CH_2_Cl_2_	**2a**(COOH)	r.t.	99%	17:1	61:39
7	**6c**	THF	**2a**(COOH)	r.t.	98%	4:1	82:18
8	**6c**	CHCl_3_	**2a**(COOH)	r.t.	93%	5:1	88:12
9	**6c**	CH_3_CN	**2a**(COOH)	r.t.	99%	3:1	54:46
10	**6c**	CH_2_ClCH_2_Cl	**2a**(COOH)	r.t.	98%	2:1	81:19
11	**6c**	CHCl_3_	**2a**(COOH)	0 °C	92%	5:1	90:10
12	**6c**	CHCl_3_	**2a**(COOH)	−20 °C	82%	7:1	90:10
13 ^b^	**6c**	CHCl_3_	**2a**(COOH)	0 °C	90%	4:1	90:10
14 ^c^	**6c**	CHCl_3_	**2a**(COOH)	0 °C	93%	5:1	90:10
15 ^c,d^	**6c**	CHCl_3_	**2a**(COOH)	0 °C	94%	5:1	90:10
16 ^c,e^	**6c**	CHCl_3_	**2a**(COOH)	0 °C	93%	5:1	90:10

^a^ All reactions were performed in a 0.10 mmol scale using **2a** or **5** (1.0 equiv.) and **3a** (1.0 equiv.) in the presence of the corresponding catalyst **6a-e** (20 mol%) in the solvent (0.2 mL) for 24h. ^b^ Reaction was performed in CHCl_3_ (0.4 mL). ^c^ Reaction was performed in CHCl_3_ (0.1 mL). ^d^ Reaction was performed using 10 mol % of **6c**. ^e^ Reaction was performed using **6c** (5 mol %).

**Table 2:** Enantioselective, decarboxylative (3+2)-cycloaddition of chromone-3-carboxylic acids 2 and iminodihydrofuran-2-one **4**—optimization studies ^a^.

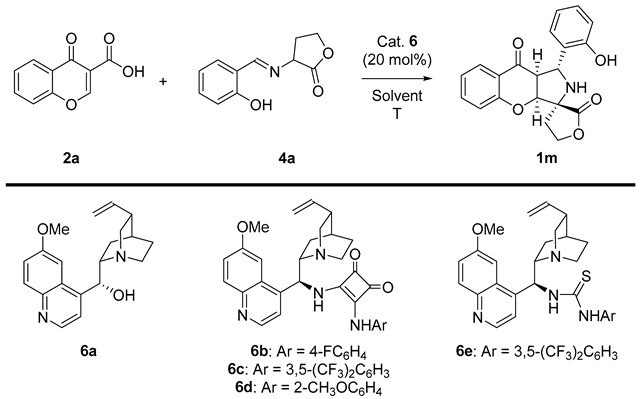						
**No.**	**Catalyst**	**Solvent**	**Temp.**	**Yield [%]**	**dr**	**er (major/minor)**
1	**6a**	CH_2_Cl_2_	r.t.	85%	1.5:1	60:40/55:45
3	**6b**	CH_2_Cl_2_	r.t.	71%	1.8:1	72:28/67:33
4	**6c**	CH_2_Cl_2_	r.t.	82%	1.5:1	98:2/74:26
5	**6d**	CH_2_Cl_2_	r.t.	70%	1.6:1	80:20/66:34
6	**6e**	CH_2_Cl_2_	r.t.	90%	1.8:1	78:22/60:40
7	**6c**	THF	r.t.	87%	1.4:1	89:11/66:34
8	**6c**	CHCl_3_	r.t.	83%	1.3:1	88:12/85:15
9	**6c**	CH_3_CN	r.t.	94%	1.5:1	62:38/59:41
10	**6c**	ClCH_2_CH_2_Cl	r.t.	88%	1.2:1	91:9/75:25
11	**6c**	CH_2_Cl_2_	0 °C	71%	2:1	91.5:8.5/84:16
12	**6c**	CH_2_Cl_2_	−20 °C	-	-	-
13 ^b^	**6c**	CH_2_Cl_2_	0 °C	70%	2:1	91.5:8.5/84:16
14 ^c^	**6c**	CH_2_Cl_2_	0 °C	39%	2:1	91.5:8.5/84:16
15 ^b,d^	**6c**	CH_2_Cl_2_	0 °C	52%	2:1	91.5:8.5/84:16
16 ^b,e^	**6c**	CH_2_Cl_2_	0 °C	41%	2:1	91.5:8.5/84:16
17 ^b,f^	**6c**	CH_2_Cl_2_	0 °C	87%	2:1	91.5:8.5/84:16

^a^ All reactions were performed on a 0.10 mmol scale using **2a** (1.0 equiv.) and **4a** (1.0 equiv.) in the presence of the corresponding catalyst **6a-e** (20 mol%) in the solvent (0.2 mL) for 24 h. ^b^ Reaction was performed in CH_2_Cl_2_ (0.4 mL). ^c^ Reaction was performed in CH_2_Cl_2_ (0.1 mL). ^d^ Reaction was performed using **6c** (10 mol%). ^e^ Reaction was performed using **6c** (5 mol%). ^f^ Reaction performed for 48 h.

## Data Availability

Data are contained within the article and Supplementary Materials.

## Supplementary Materials

The following supporting information can be downloaded at: https://www.mdpi.com/article/10.3390/molecules27206809/s1, Section 1: General methods; Section 2: Crystal and X-ray data; Section 3: NMR data; Section 4: HPLC data.
