# Supplementary material for: Enantioselective, Decarboxylative (3+2)-Cycloaddition of Azomethine Ylides and Chromone-3-Carboxylic Acids
Source: Molecules. 2022 Oct 11;27(20):6809. doi: 10.3390/molecules27206809 (PMC9607314; doi:10.3390/molecules27206809)
Supplement: Supplementary file 1 [file molecules-27-06809-s001.zip › molecules-1938635-supplementary.pdf]

Enantioselective, decarboxylative [3+2] cycloaddition of azomethine ylides and chromone-3-carboxylic acids

Ewelina Kowalska,[a] Lesław Sieroń[b] and Anna Albrecht[b*]

[a] Institute of Organic Chemistry, Lodz University of Technology, Żeromskiego 116, 90-924 Łódź, Poland
[b] Institute of General and Ecological Chemistry, Faculty of Chemistry, Lodz University of Technology, Żeromskiego 116, 90-924 Łódź, Poland
anna.albrecht@p.lodz.pl

Contents

**1.**	General methods	S2
**2.**	Crystal and X-ray data	S3
**3.**	NMR data	S5
**4.**	HPLC data	S39

1. General methods

NMR spectra were acquired on a Bruker Ultra Shield 700 instrument, running at 700 MHz for ^{1}H and 176 MHz for ^{13}C, respectively. Chemical shifts (δ) are reported in ppm relative to residual solvent signals ($CDCl_3$: 7.26 ppm for ^{1}H NMR, 77.16 ppm for ^{13}C NMR). Mass spectra were recorded on a Bruker Maxis Impact spectrometer using electrospray (ES+) ionization (referenced to the mass of the charged species). Analytical thin layer chromatography (TLC) was performed using pre-coated aluminum backed plates (Merck Kieselgel 60 F254) and visualized by ultraviolet irradiation. Unless otherwise noted, analytical grade solvents and commercially available reagents were used without further purification. For flash chromatography (FC) silica gel (Silica gel 60, 230-400 mesh, Fluka and Davisil Grade 643, 200-425 mesh). The enantiomeric ratio (er) of the products were determined by Ultra Performance Convergence Chromatography (UPC2) or HPLC using Daicel Chiralpak IA, IB, IC and IG columns as chiral stationary phases. Diethyl 2-hydroxyarylideneaminomalonates **3** and 2-hydroxyarylideneaminolactones **4** were synthetized according to the literature procedure[1]. Chromone-3- carboxylic acids[2] **2** were prepared from the corresponding substituted 2-hydroxybenzaldehydes following the literature procedure.

---

[1] Tian, L., Xu, G. Q., Li, Y. H., Liang, Y. M., Xu, P. F., *Chemical Community*, **2014**, *50*(19), 2428-2430

[2] Ishizuka, N., Matsumura, K. I., Sakai, K., Fujimoto, M., Mihara, S. I., Yamamori, T., *Journal of medicinal chemistry*, **2002**, *45*(10), 2041-2055.

2. Crystal and X-ray data

Single-crystal X-ray diffraction data were collected at temperature of 100 K. The compound **1e** ($C_{23}H_{22}BrNO_7$) crystallizes in centrosymmetric triclinic space group *P*-1 as racemic mixture, and **1m** ($C_{20}H_{17}NO_5$) crystallizes in the non-centrosymmetric orthorhombic space group $P2_12_12_1$. The asymmetric unit of both crystals contains one crystallographically independent molecule (Fig. **1**).

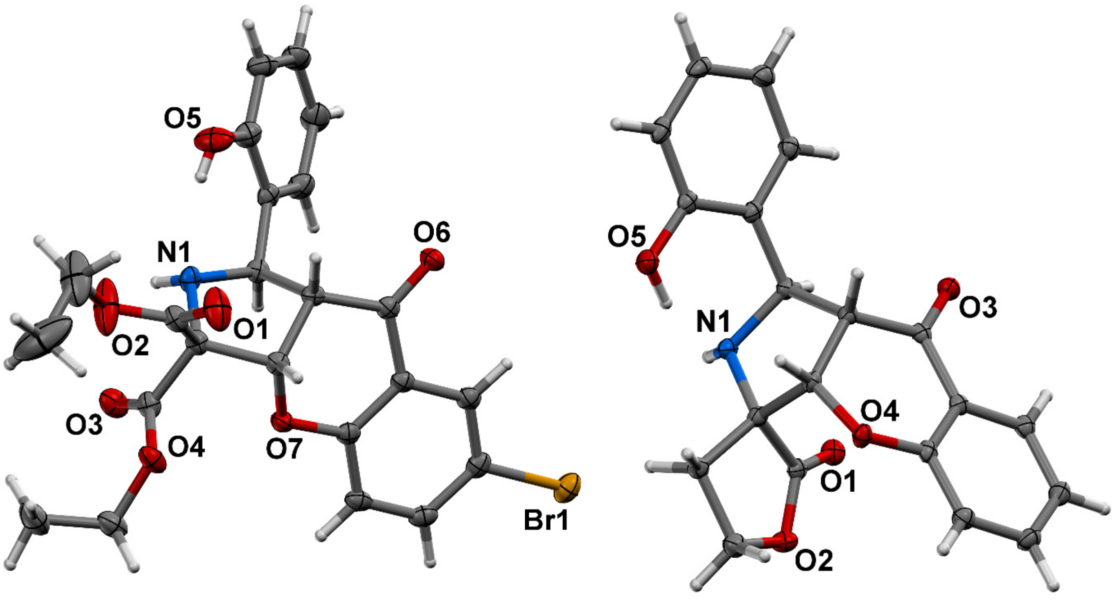

a) b)

Figure S1. Views of the molecules of **1e** (a) and **1m** (b) at 100 K, with the atom-labeling schemes and 50% probability ellipsoids. Hydrogen atoms are drawn with an arbitrary radius.

Single crystal X-ray diffraction data were collected at 100 K by the ω-scan technique using a RIGAKU XtaLAB Synergy, Dualflex, Pilatus 300K diffractometer[3] with PhotonJet micro-focus X-ray Source Cu-Kα (λ = 1.54184 Å). Data collection, cell refinement, data reduction and absorption correction were performed using CrysAlis PRO software.[3] The crystal structure was solved by using direct methods with the SHELXT 2018/2 program.[4] Atomic scattering factors

[3] Rigaku OD. CrysAlis PRO. Rigaku Oxford Diffraction Ltd, Yarnton, Oxfordshire, England, **2019**.
[4] Sheldrick, G.M. "SHELXT - integrated space-group and crystal-structure determination", *Acta Cryst*. **2015**, *A71*, 3-8.

were taken from the International Tables for X-ray Crystallography. Positional parameters of non-H-atoms were refined by a full-matrix least-squares method on F^2 with anisotropic thermal parameters by using the SHELXL 2018/3 program.[5] All hydrogen atoms were placed in calculated positions (C–H = 0.95–1.00 Å) and included as riding contributions with isotropic displacement parameters set to 1.2-1.5 times the U_{eq} of the parent atom.

1e: Formula $C_{23}H_{22}BrNO_7$, triclinic, space group *P*-1, *Z* = 4, unit cell constants *a* = 11.1608(1), *b* = 14.1880(2), *c* = 16.0038(2) Å, α = 69.852(1), β = 75.621(1) , γ = 74.609(1)° , *V* = 2259.06(5) $Å^3$. The integration of the data yielded a total of 53433 reflections with θ angles in the range of 2.99 to 66.59° of which 7974 were unique (R_{int} = 2.69%), and 7815 were greater than $2\sigma(F^2)$. The final anisotropic full-matrix least-squares refinement on F^2 with 597 parameters converged to the final R_1 = 0.0320 (for $I > 2\sigma(I)$) and wR_2 = 0.0806 (all data). The goodness-of-fit was 1.037. The largest peak in the final difference electron density synthesis was 0.93 e $Å^{-3}$ and the largest hole was -0.67 e $Å^{-3}$.

1m: Formula $C_{20}H_{17}NO_5$, orthorhombic, space group $P2_12_12_1$, *Z* = 4, unit cell constants *a* = 6.6243(1), *b* = 10.9183(1), *c* = 22.0348(1) Å, *V* = 1593.69(3) $Å^3$. The integration of the data yielded a total of 37878 reflections with θ angles in the range of 4.01 to 66.58 of which 2806 reflections were unique (R_{int} = 2.46%), and 2786 were greater than $2\sigma(F^2)$. The final anisotropic full-matrix least-squares refinement on F^2 with 244 parameters converged to the final R_1 = 0.0211 (for $I > 2\sigma(I)$) and wR_2 = 0.0530 for all data. The goodness-of-fit was 1.056. The largest peak in the final difference electron density synthesis was 0.20 e $Å^{-3}$ and the largest hole was -0.12 e $Å^{-3}$. The absolute configuration was determined from anomalous scattering, by calculating the x Flack parameter[6] of 0.00(2) using 1154 quotients.

CCDC 2167375 (**1e**) and CCDC 2167382 (**1m**) contain the supplementary crystallographic data for this paper. These data can be obtained free of charge from The Cambridge Crystallographic Data Centre via www.ccdc.cam.ac.uk/structure.

[5] Sheldrick, G.M. "Crystal structure refinement with SHELXL", *Acta Cryst*. **2015**, *C71*, 3-8.
[6] Parsons, S.; Flack, H.D.; Wagner, T. "Use of intensity quotients and differences in absolute structure refinement" *Acta Cryst*. **2013**, *B69*, 249-259.

3. NMR Data

(1*R*,3a*S*,9a*S*)-Diethyl 1-(2-hydroxyphenyl)-9-oxo-1,2,9,9a-tetrahydrochromeno[2,3-*c*]pyrrole-3,3(3a*H*)-dicarboxylate 1a

^{1}H NMR

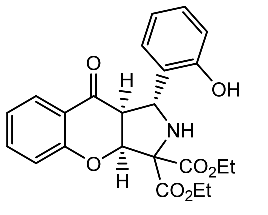

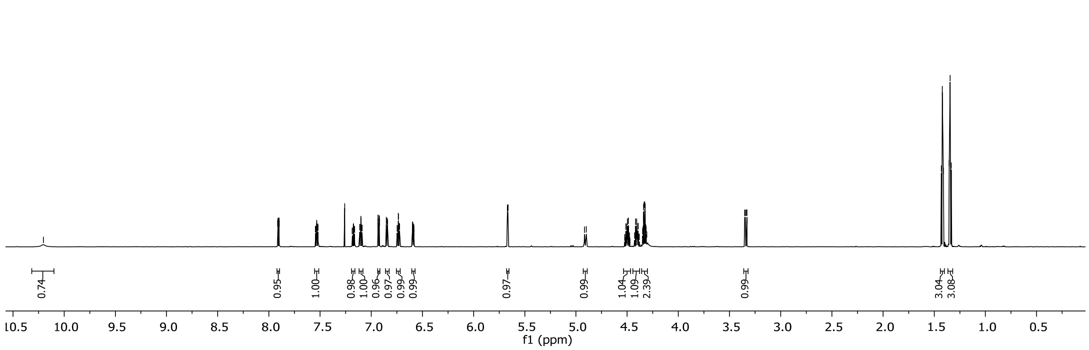

^{13}C NMR

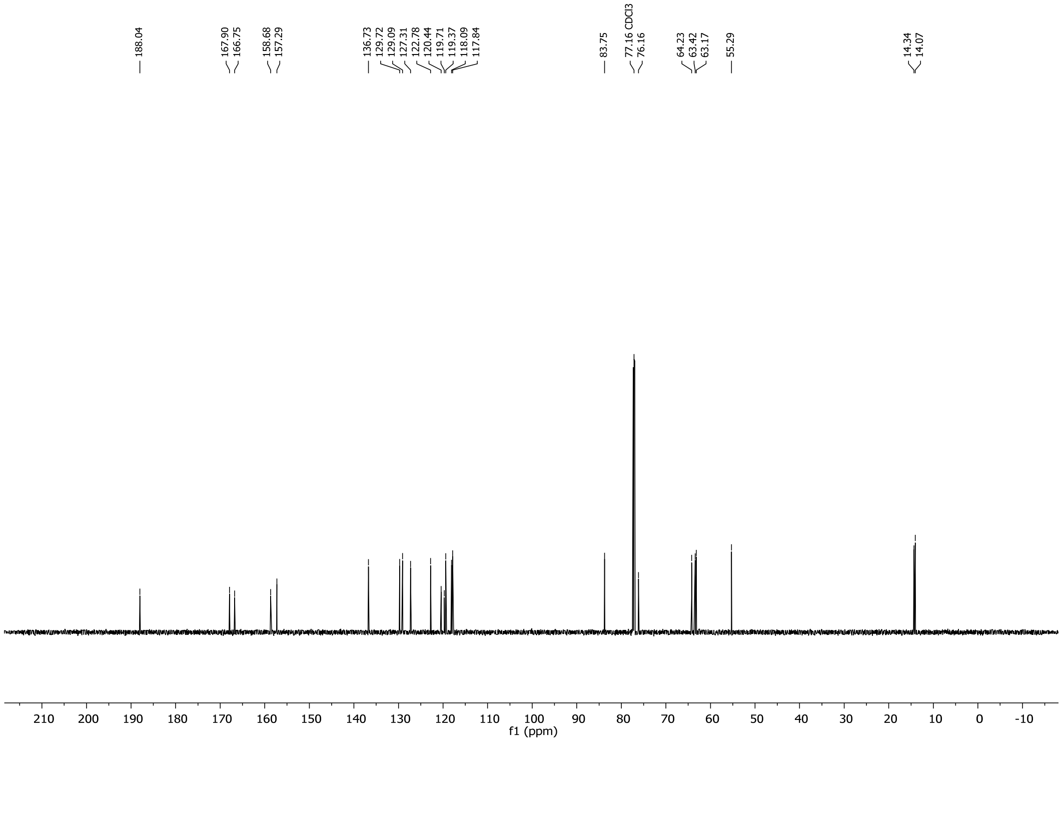

(1*R*,3a*S*,9a*S*)-Diethyl 1-(2-hydroxyphenyl)-7-methyl-9-oxo-1,2,9,9a-tetrahydrochromeno[2,3-*c*]pyrrole-3,3(3a*H*)-dicarboxylate 1b

^{1}H NMR

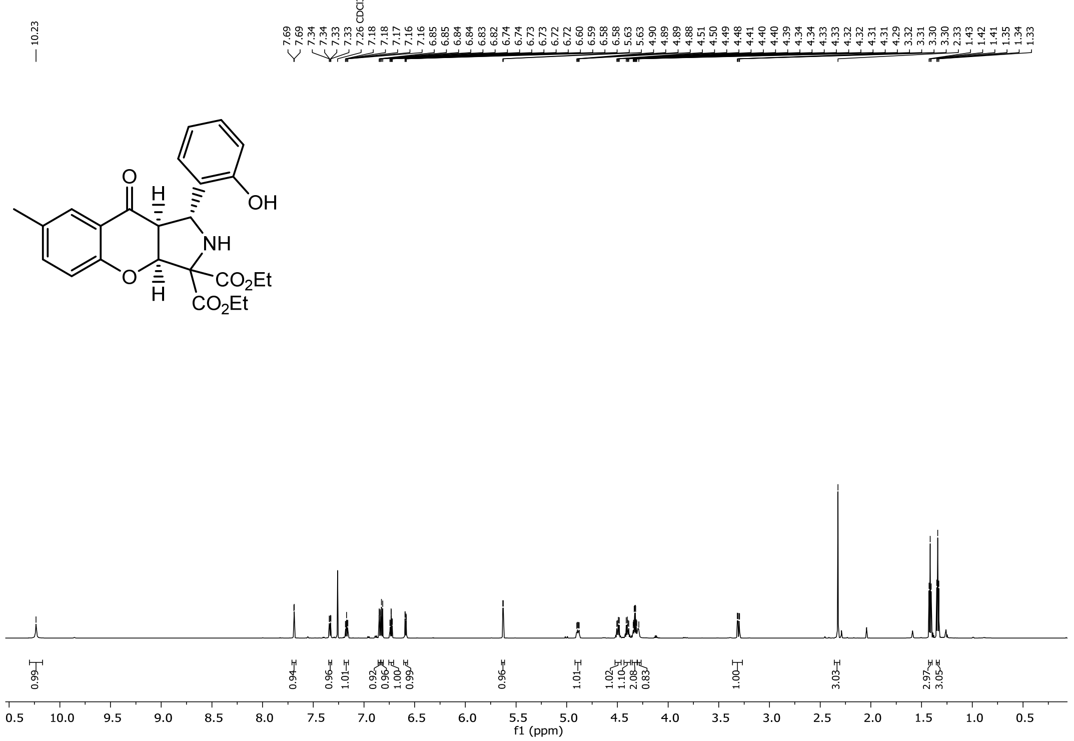

^{13}C NMR

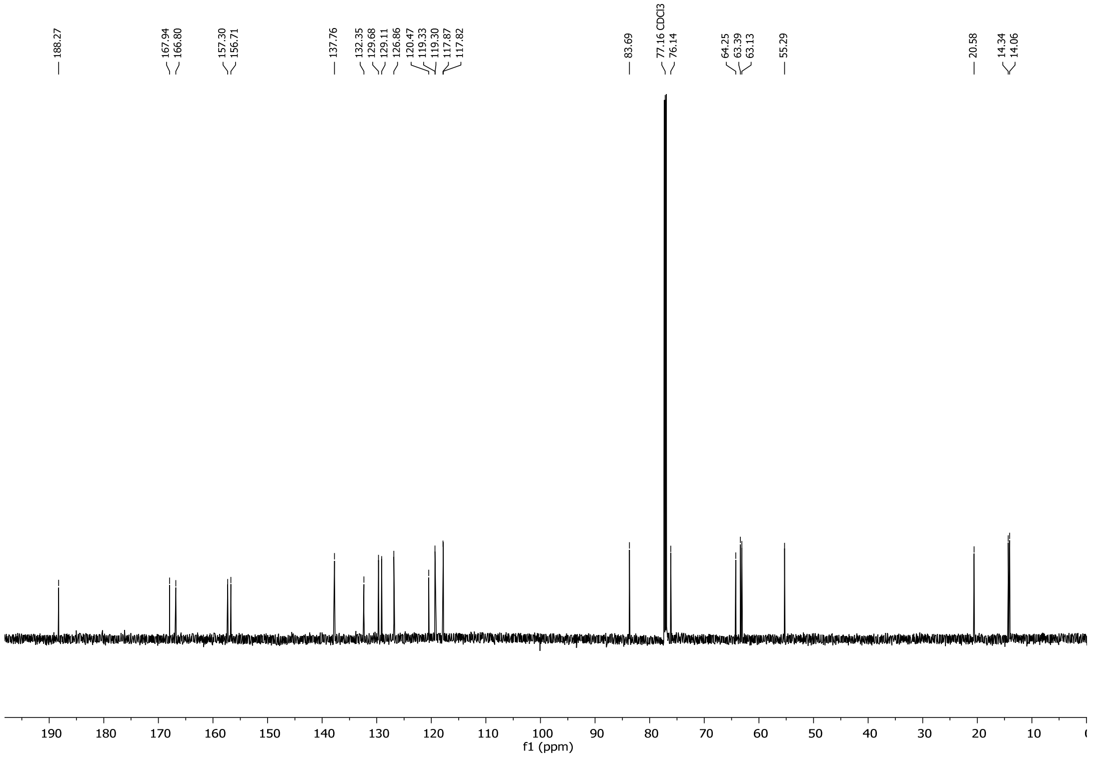

(1*R*,3a*S*,9a*S*)-Diethyl 1-(2-hydroxyphenyl)-6-methyl-9-oxo-1,2,9,9a-tetrahydrochromeno[2,3-*c*]pyrrole-3,3(3a*H*)-dicarboxylate 1c

^{1}H NMR

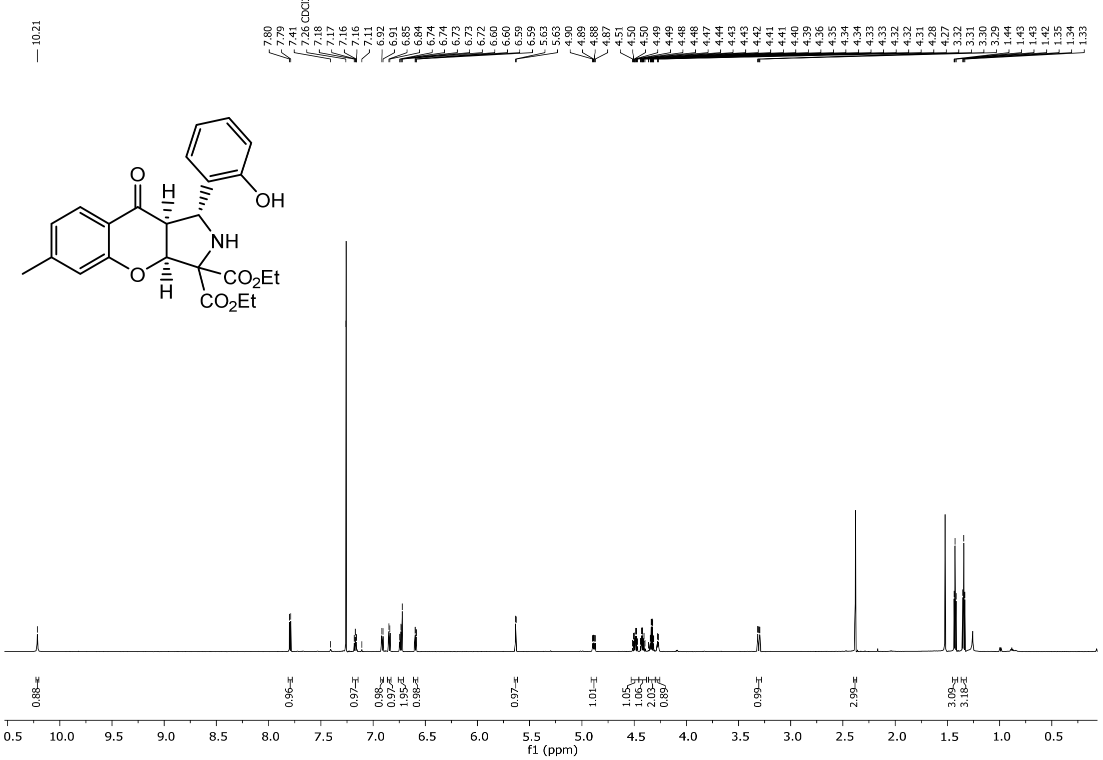

^{13}C NMR

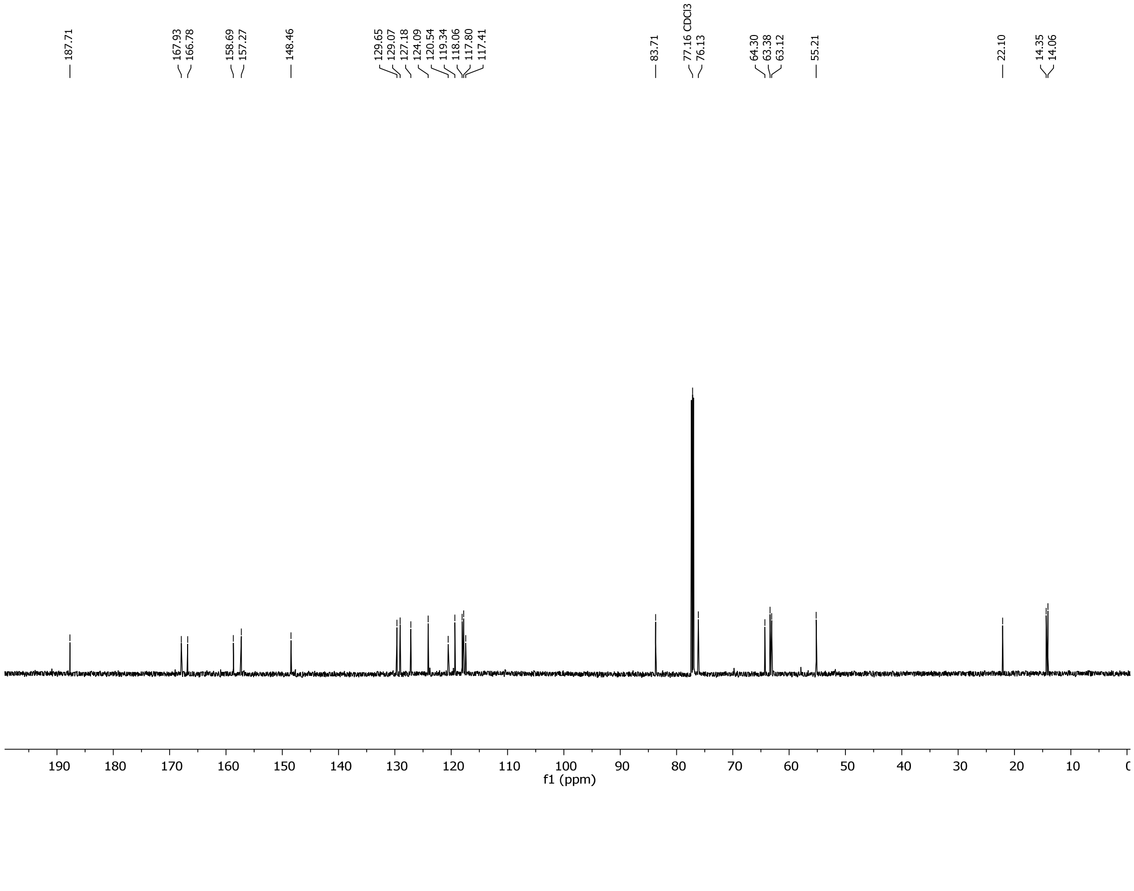

(1*R*,3a*S*,9a*S*)-Diethyl 7-fluoro-1-(2-hydroxyphenyl)-9-oxo-1,2,9,9a-tetrahydrochromeno[2,3-*c*]pyrrole-3,3(3a*H*)-dicarboxylate 1d

^{1}H NMR

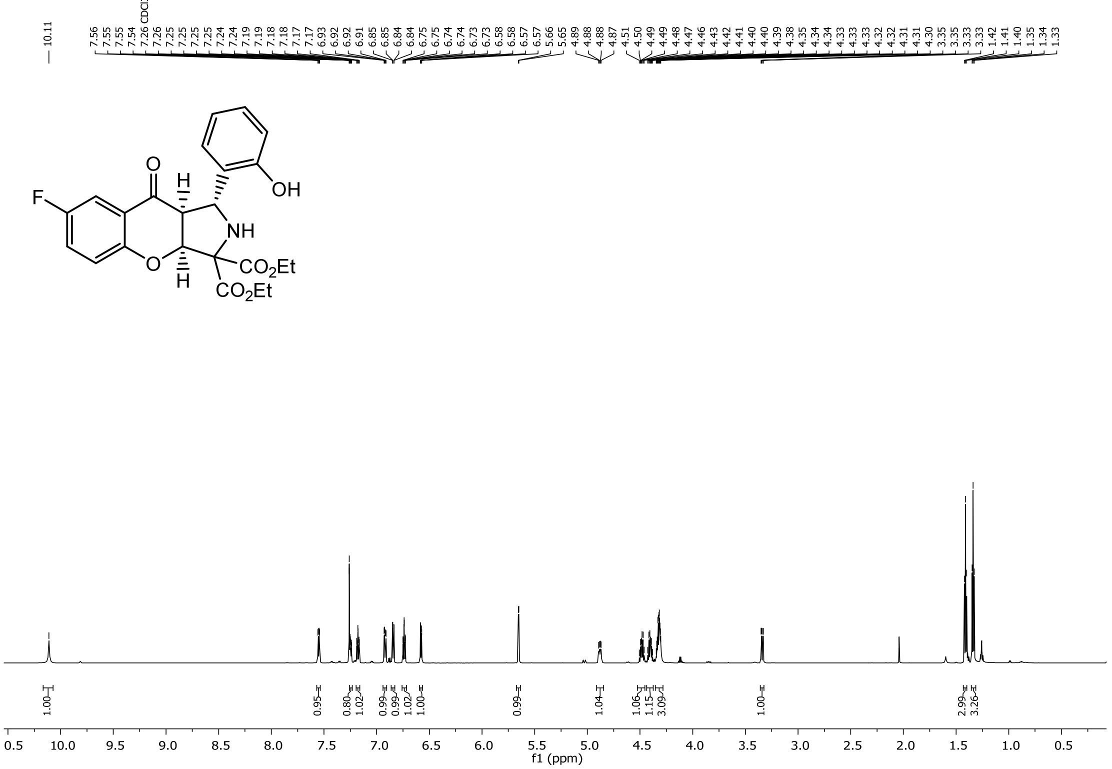

^{13}C NMR

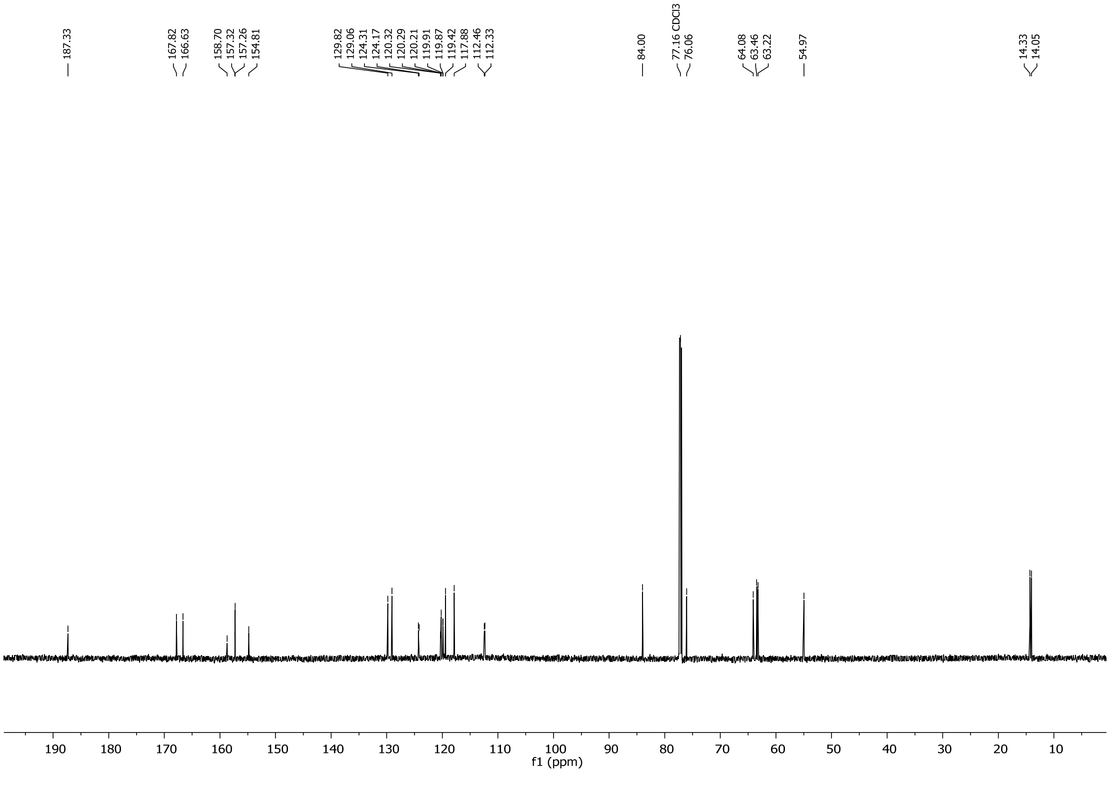

(1*R*,3a*S*,9a*S*)-Diethyl 7-bromo-1-(2-hydroxyphenyl)-9-oxo-1,2,9,9a-tetrahydrochromeno[2,3-*c*]pyrrole-3,3(3a*H*)-dicarboxylate 1e

^{1}H NMR

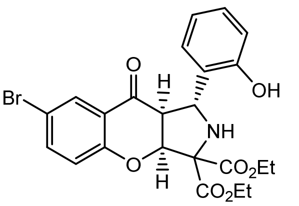

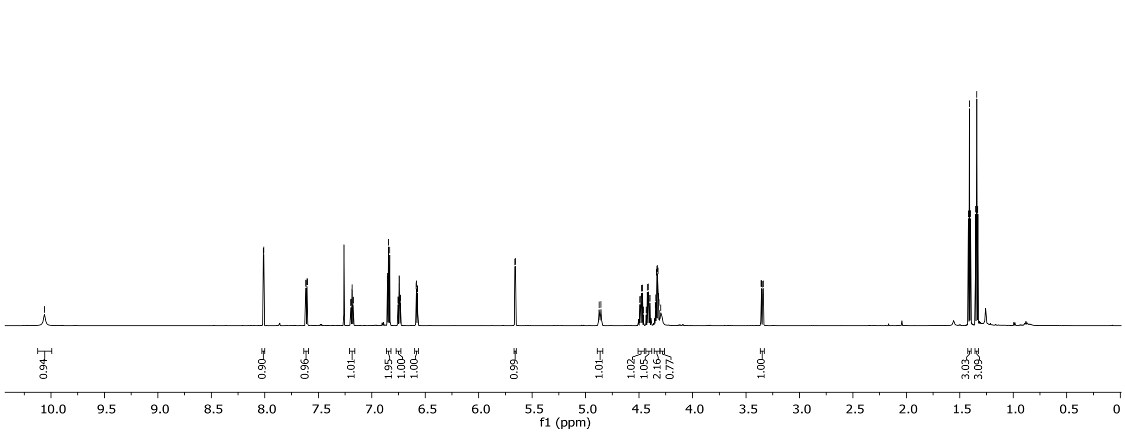

^{13}C NMR

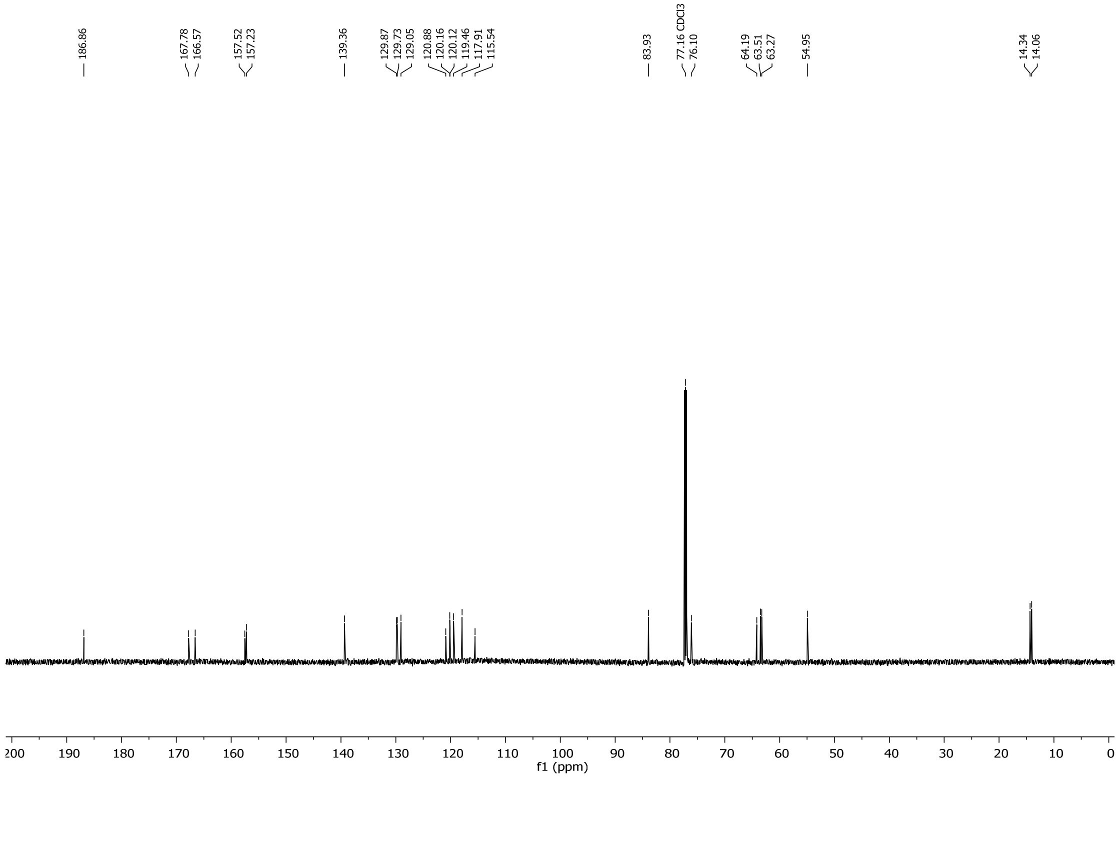

(1*R*,3a*S*,9a*S*)-Diethyl 7-chloro-1-(2-hydroxyphenyl)-9-oxo-1,2,9,9a-tetrahydrochromeno[2,3-*c*]pyrrole-3,3(3a*H*)-dicarboxylate 1f

^{1}H NMR

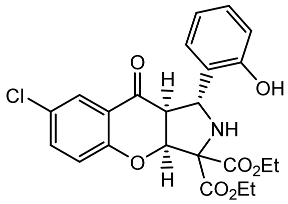

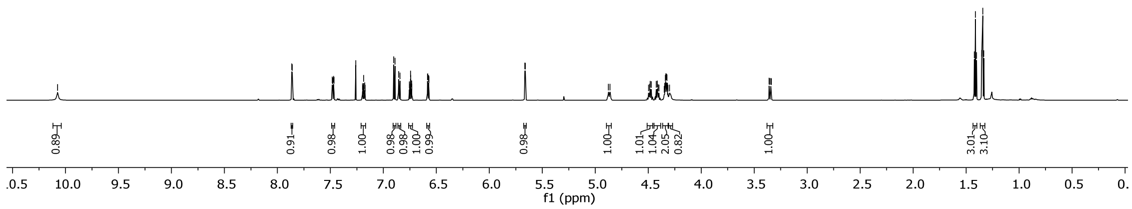

^{13}C NMR

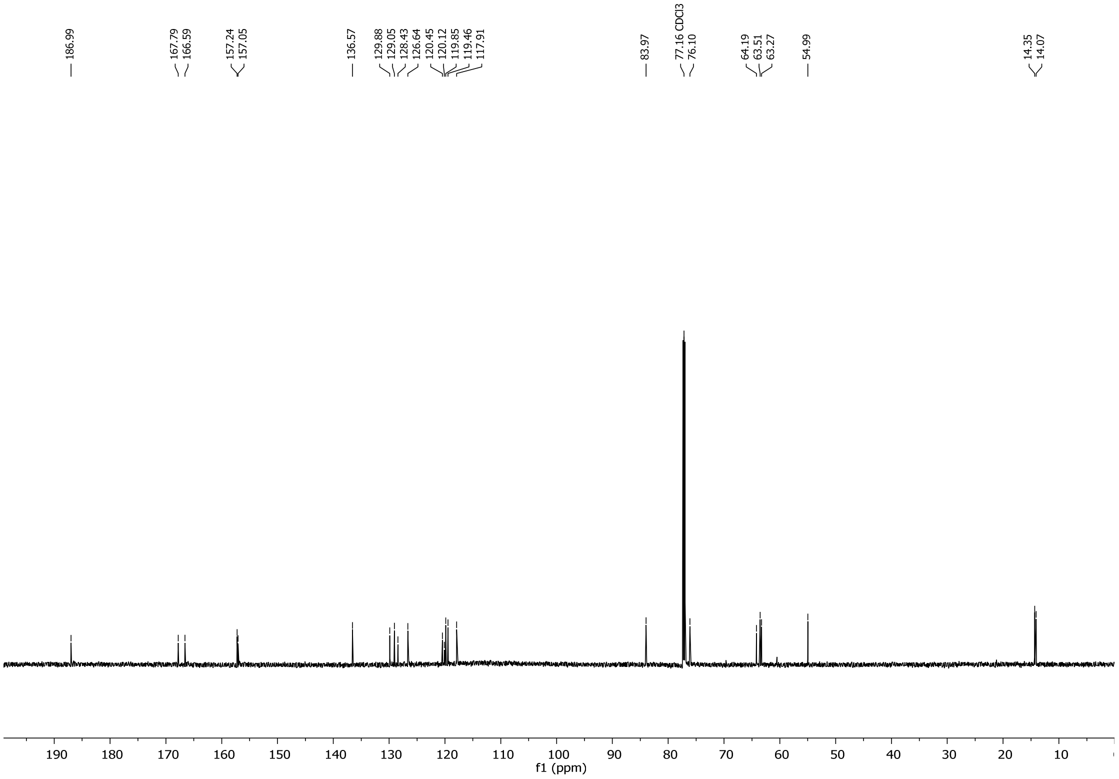

(1*R*,3a*S*,9a*S*)-Diethyl 7-chloro-1-(2-hydroxyphenyl)-6-methyl-9-oxo-1,2,9,9a-tetrahydrochromeno[2,3-*c*]pyrrole-3,3(3a*H*)-dicarboxylate 1g

^{1}H NMR

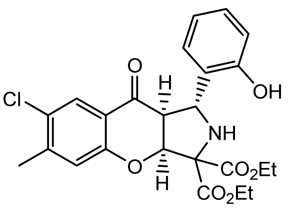

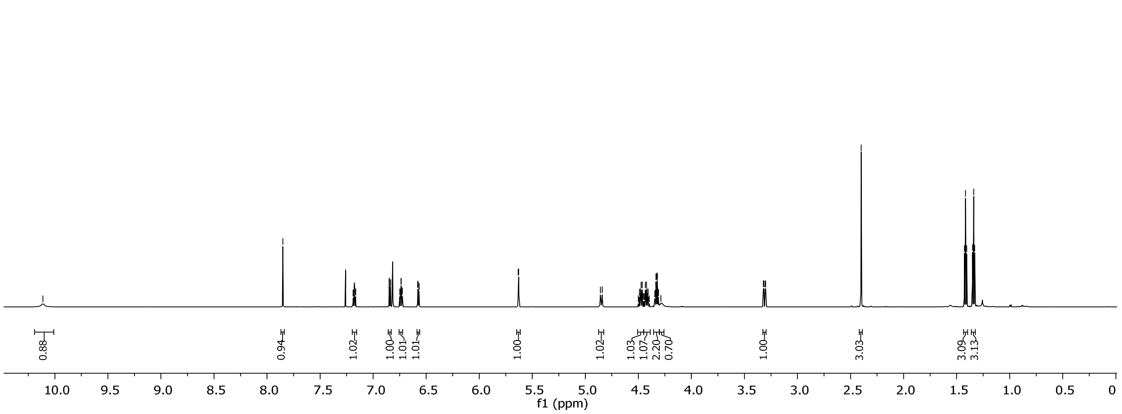

^{13}C NMR

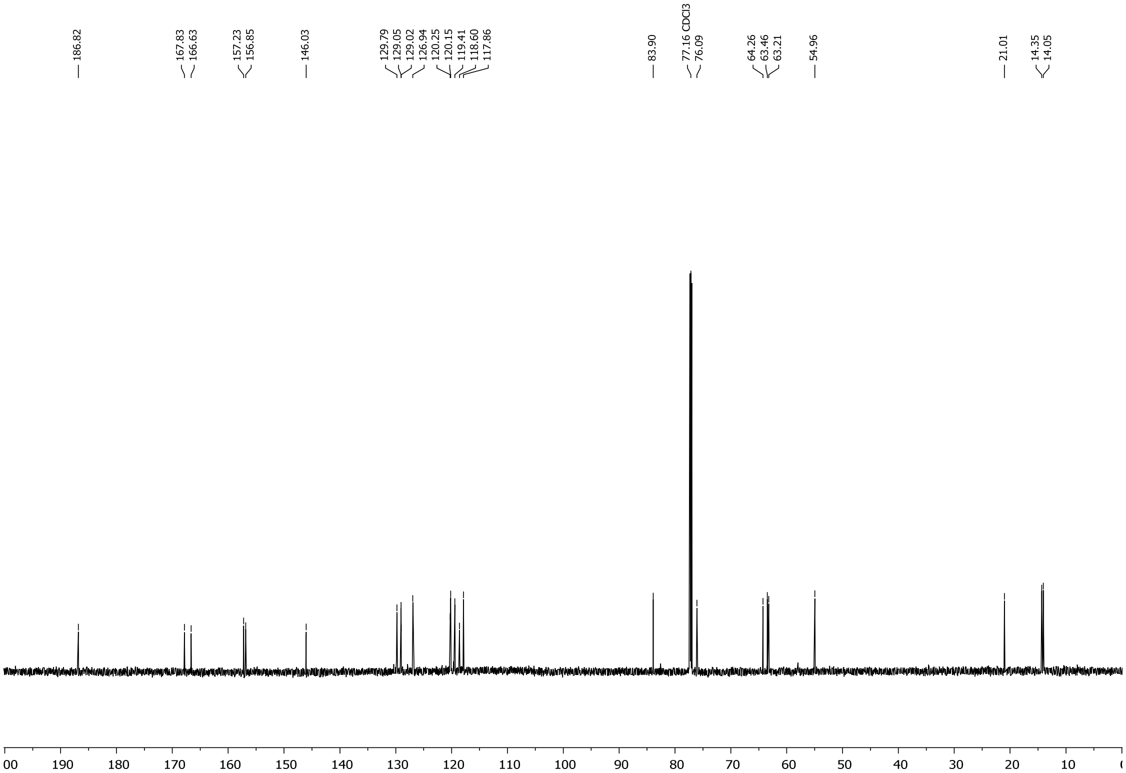

(7a*S*,10*R*,10a*S*)-Diethyl 10-(2-hydroxyphenyl)-11-oxo-9,10,10a,11-tetrahydrobenzo[5,6]chromeno[2,3-*c*]pyrrole-8,8(7a*H*)-dicarboxylate 1h

^{1}H NMR

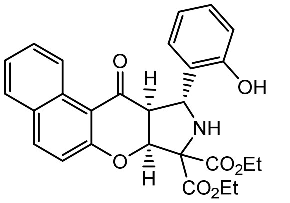

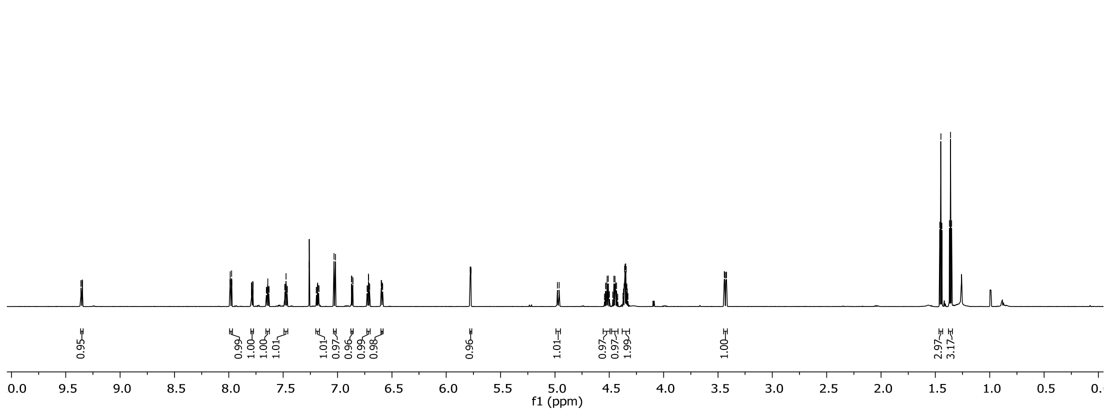

^{13}C NMR

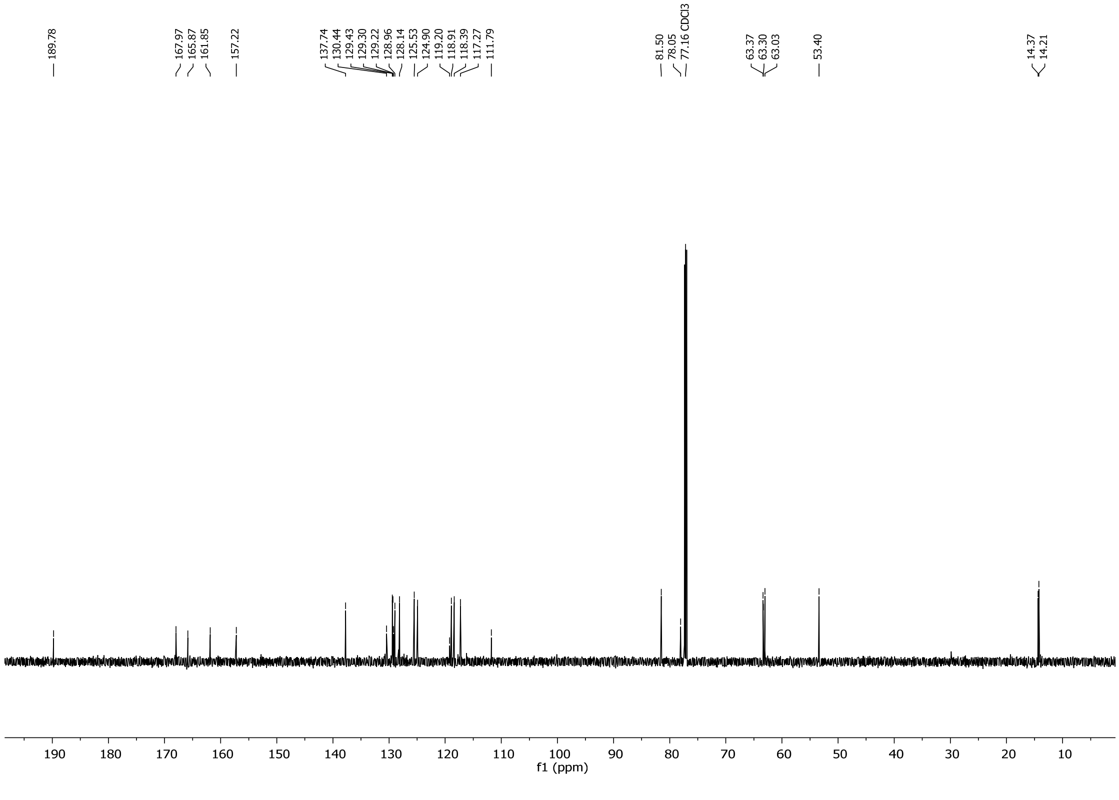

(1*R*,3a*S*,9a*S*)-Diethyl 1-(2-hydroxy-5-methylphenyl)-9-oxo-1,2,9,9a-tetrahydrochromeno[2,3-*c*]pyrrole-3,3(3a*H*)-dicarboxylate 1i

^{1}H NMR

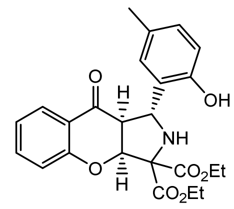

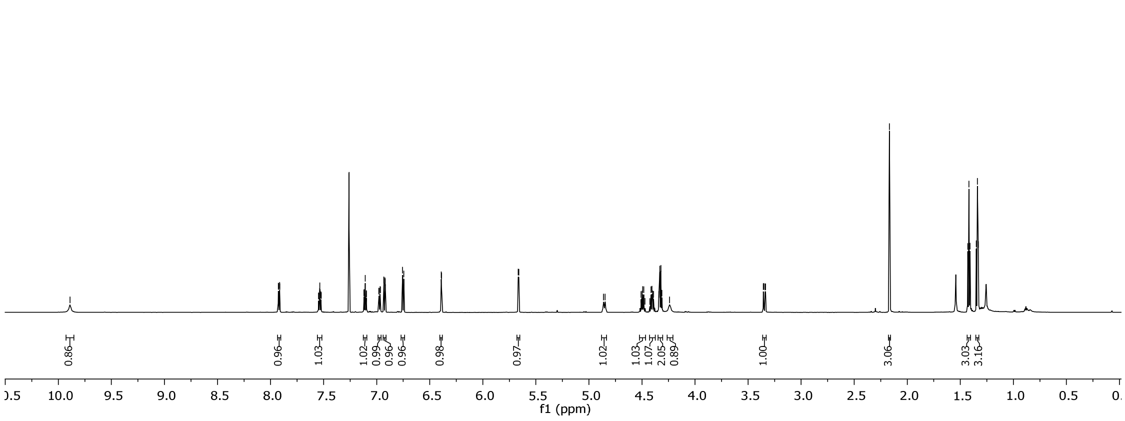

^{13}C NMR

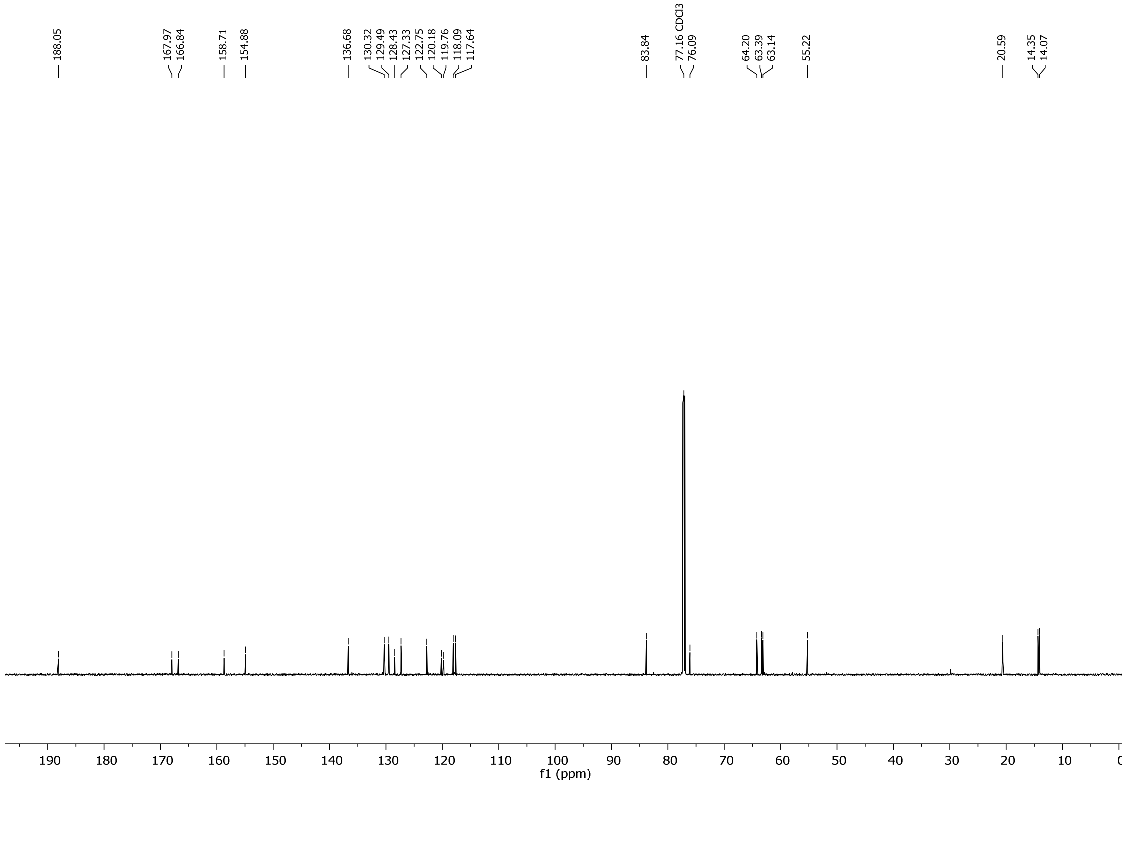

(1*R*,3a*S*,9a*S*)-Diethyl 1-(5-chloro-2-hydroxyphenyl)-9-oxo-1,2,9,9a-tetrahydrochromeno[2,3-*c*]pyrrole-3,3(3a*H*)-dicarboxylate 1j

^{1}H NMR

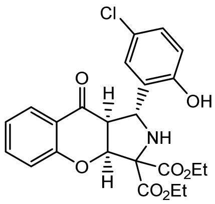

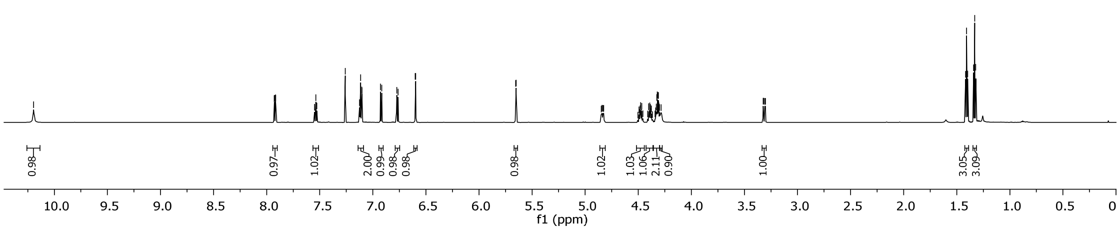

^{13}C NMR

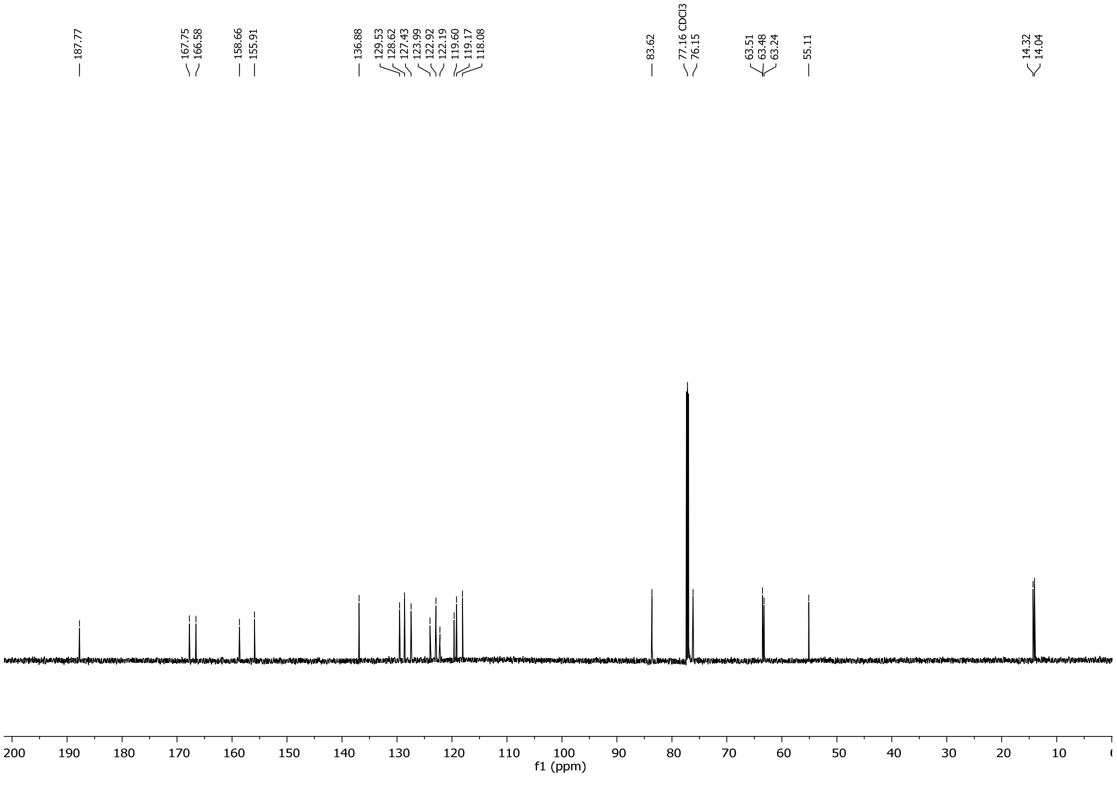

(1*R*,3a*S*,9a*S*)-Diethyl 1-(5-(*tert*-butyl)-2-hydroxyphenyl)-9-oxo-1,2,9,9a-tetrahydrochromeno[2,3-*c*]pyrrole-3,3(3a*H*)-dicarboxylate 1k

^{1}H NMR

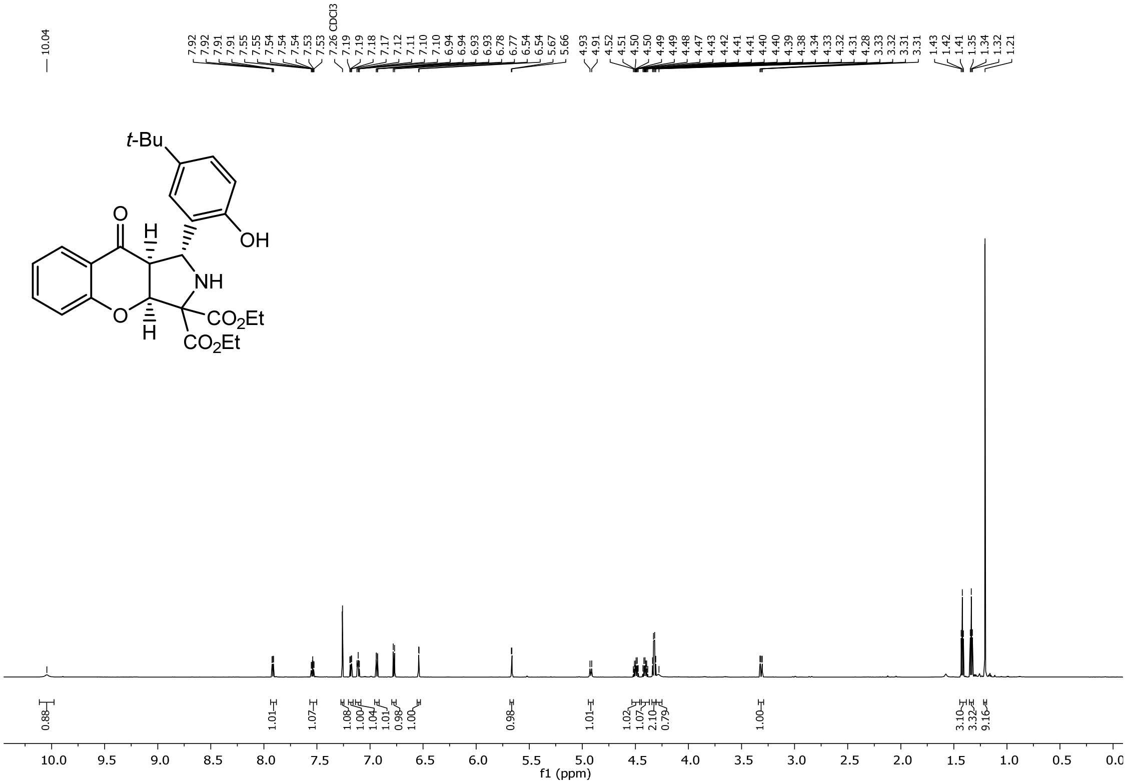

^{13}C NMR

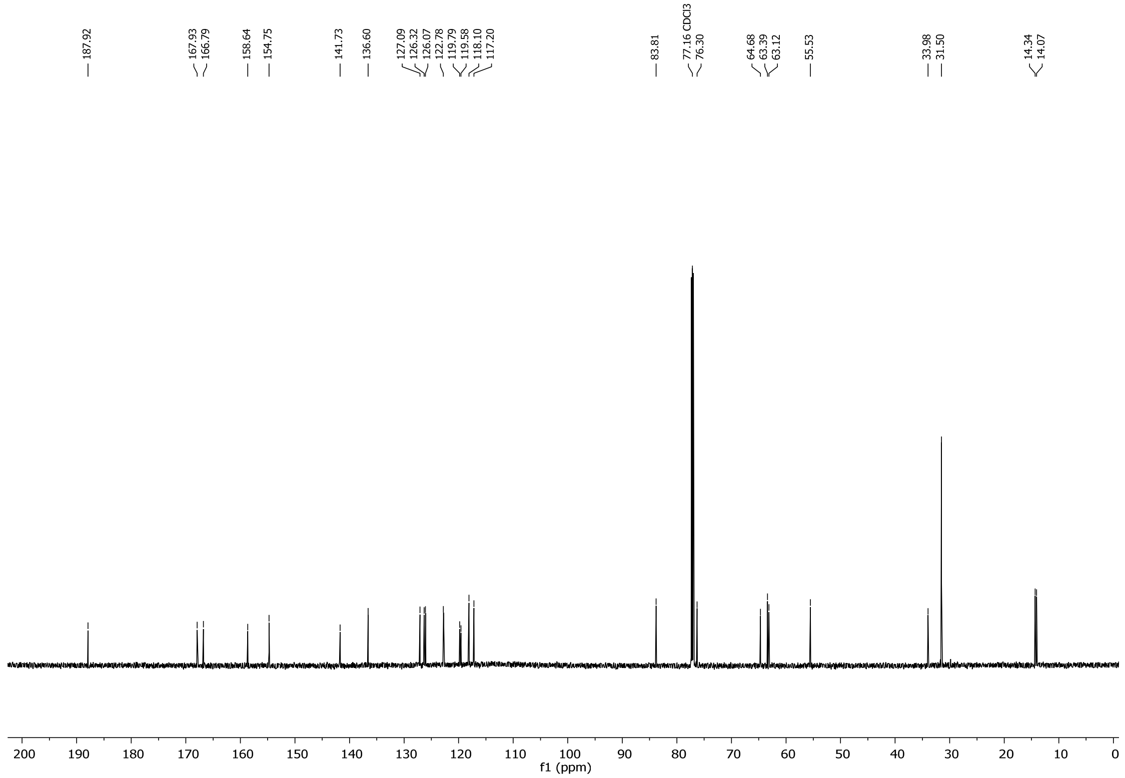

(1*R*,3a*S*,9a*S*)-Diethyl 1-(2-hydroxy-5-nitrophenyl)-9-oxo-1,2,9,9a-tetrahydrochromeno[2,3-*c*]pyrrole-3,3(3a*H*)-dicarboxylate 1l

^{1}H NMR

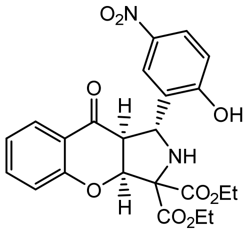

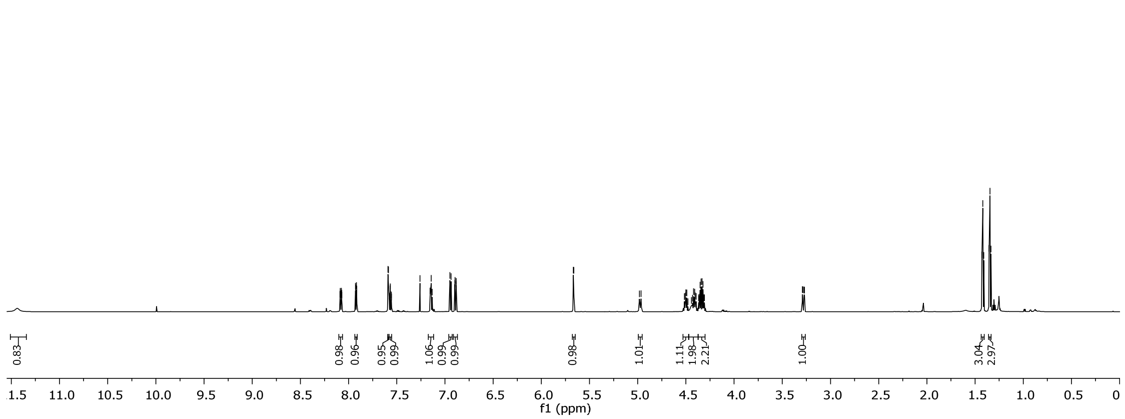

^{13}C NMR

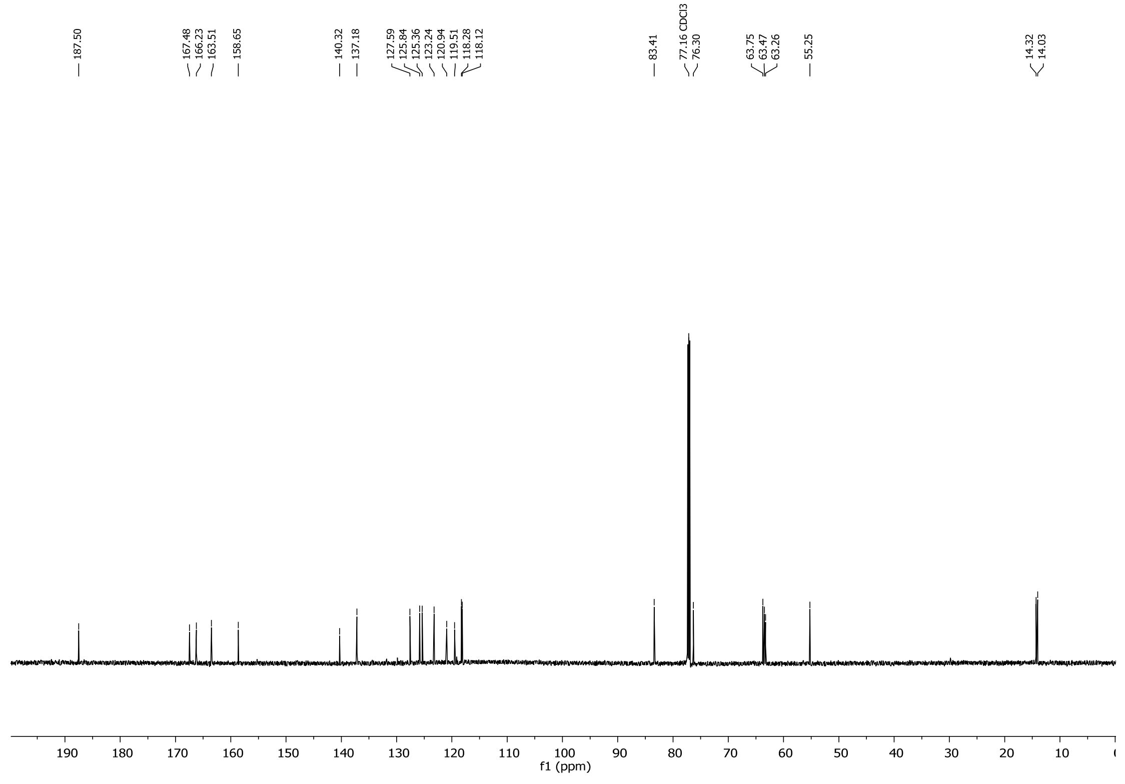

(1*R*,3*R*,3a*S*,9a*S*)-1-(2-Hydroxyphenyl)-3a,4',5',9a-tetrahydro-1*H*,2'*H*-spiro[chromeno[2,3-*c*]pyrrole-3,3'-furan]-2',9(2*H*)-dione 1m

^{1}H NMR

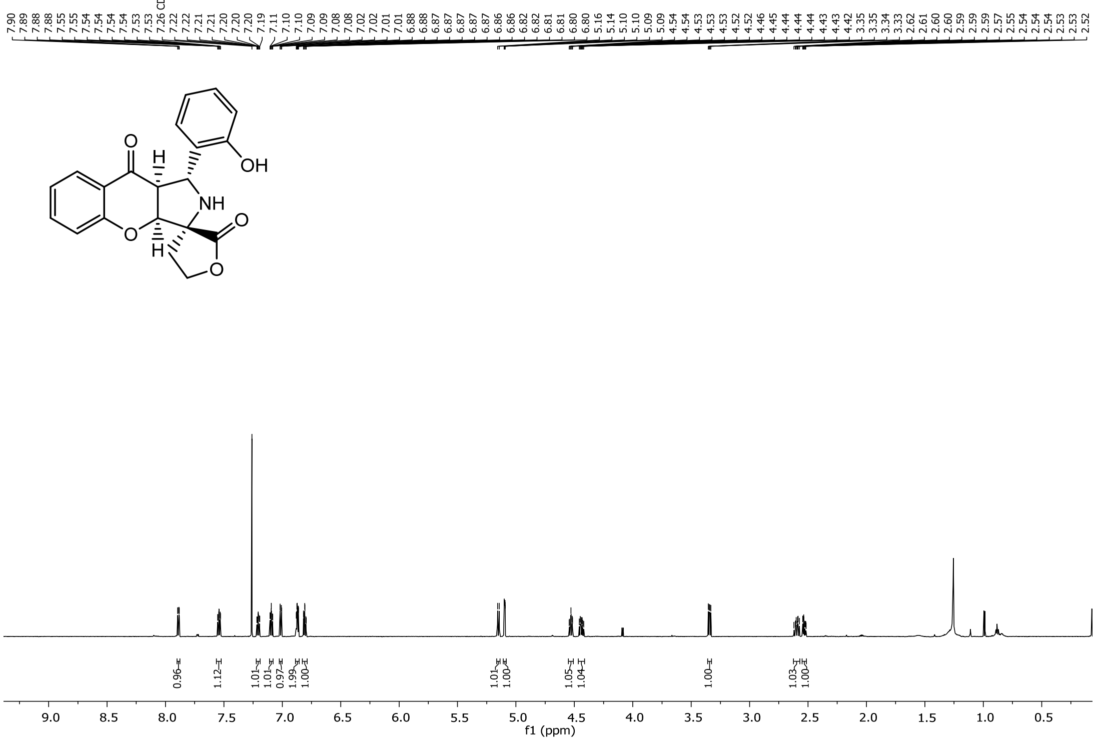

^{13}C NMR

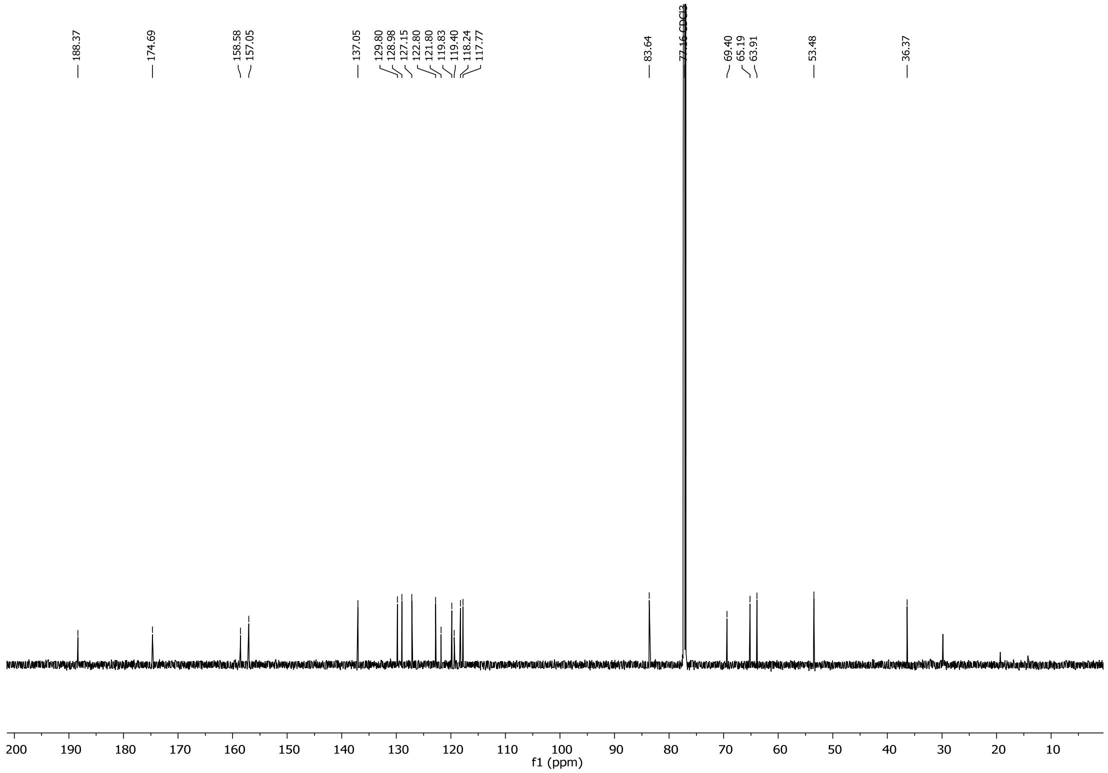

(1*R*,3*S*,3a*S*,9a*S*)-1-(2-Hydroxyphenyl)-3a,4',5',9a-tetrahydro-1*H*,2'*H*-spiro[chromeno[2,3-*c*]pyrrole-3,3'-furan]-2',9(2*H*)-dione 1m'

^{1}H NMR

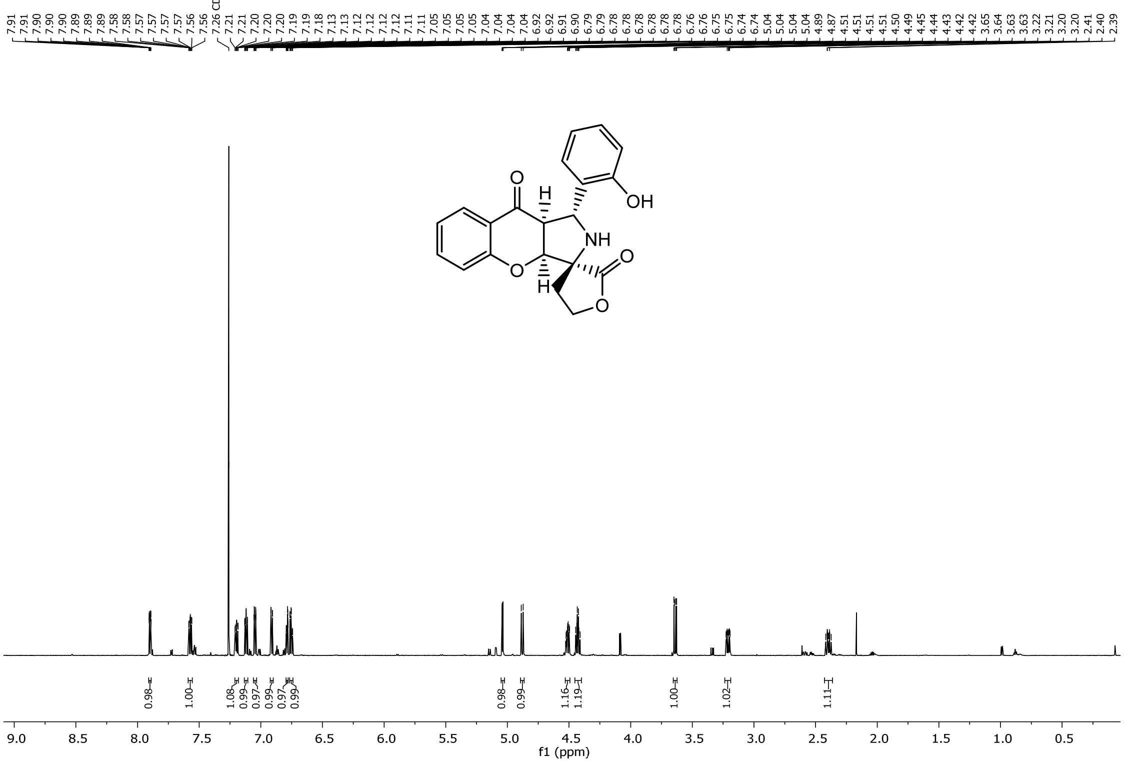

^{13}C NMR

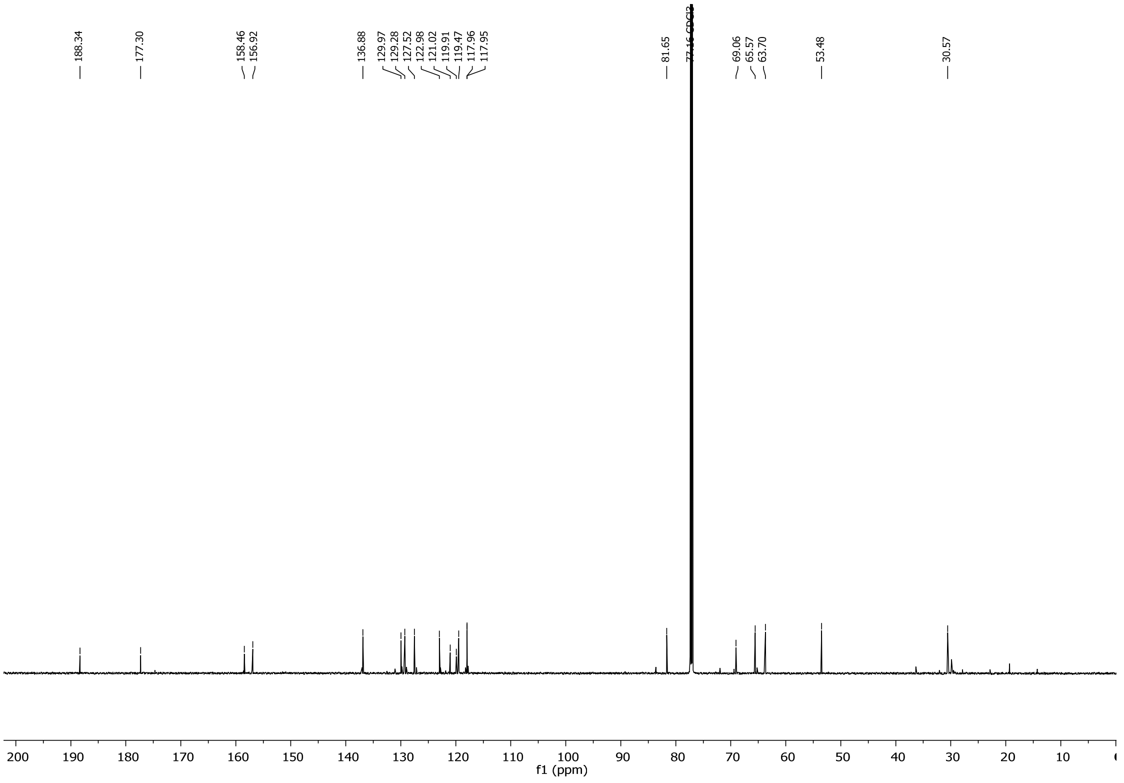

(1*R*,3*R*,3a*S*,9a*S*)-1-(2-Hydroxyphenyl)-7-methyl-3a,4',5',9a-tetrahydro-1*H*,2'*H*-spiro[chromeno[2,3-*c*]pyrrole-3,3'-furan]-2',9(2*H*)-dione 1n

^{1}H NMR

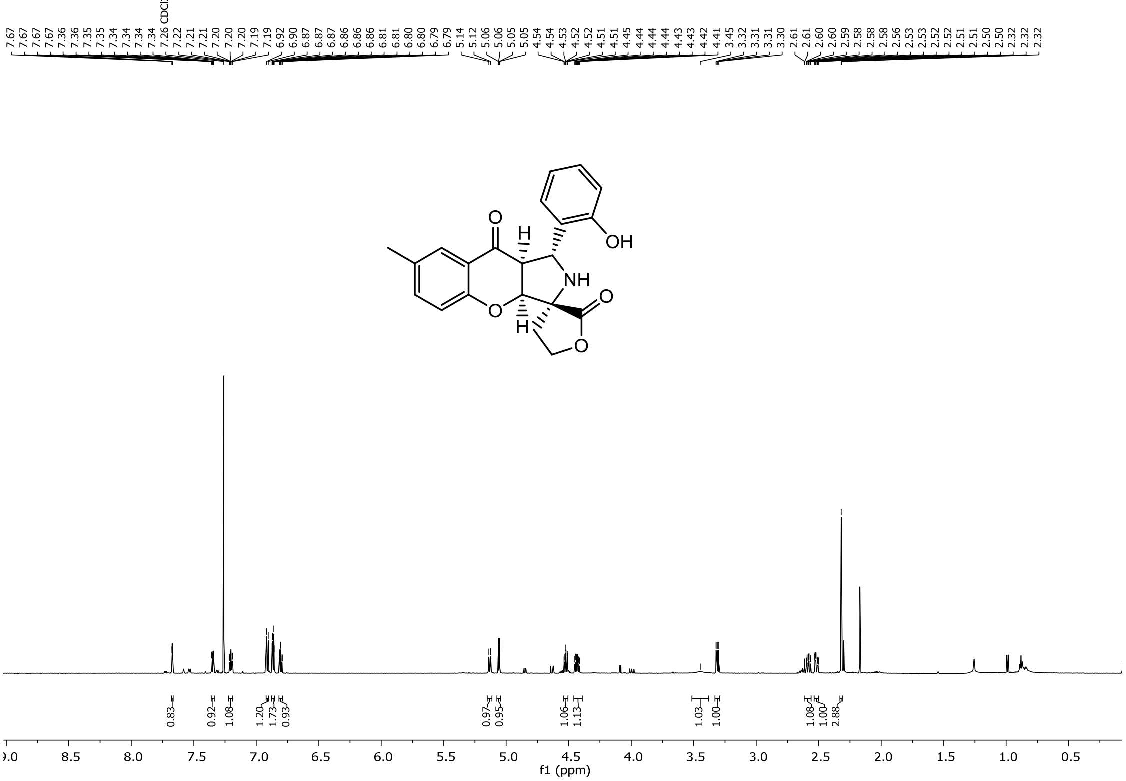

^{13}C NMR

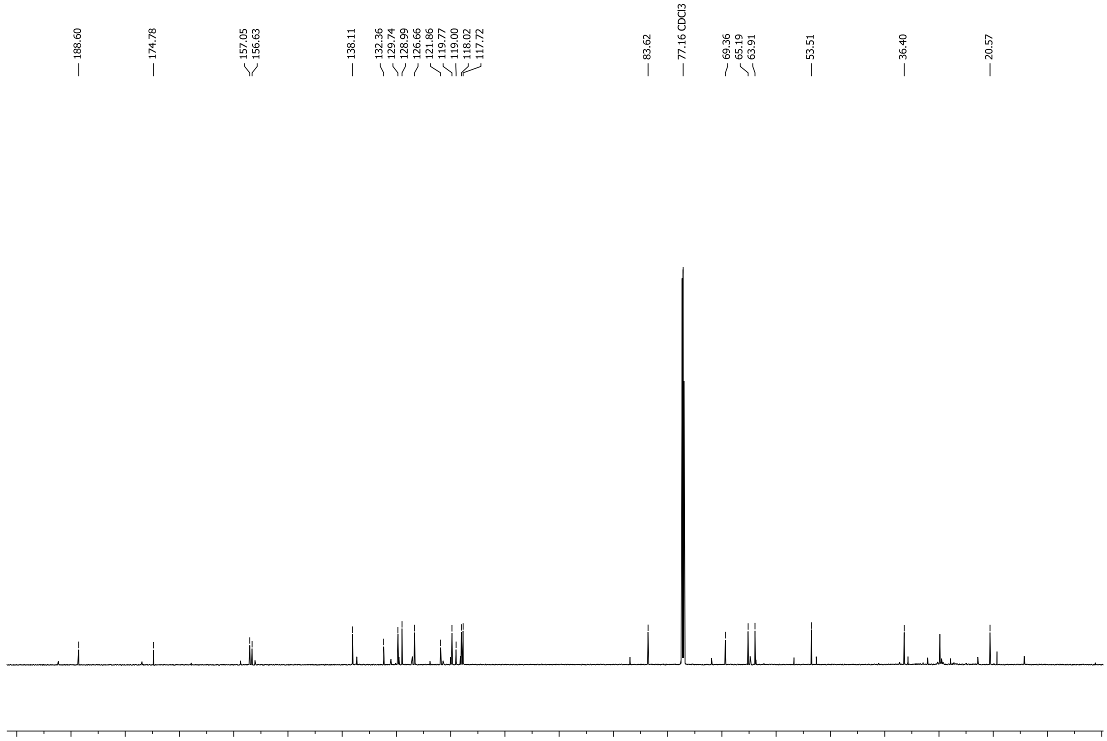

(1*R*,3*S*,3a*S*,9a*S*)-1-(2-Hydroxyphenyl)-7-methyl-3a,4',5',9a-tetrahydro-1*H*,2'*H*-spiro[chromeno[2,3-*c*]pyrrole-3,3'-furan]-2',9(2*H*)-dione 1n'

^{1}H NMR

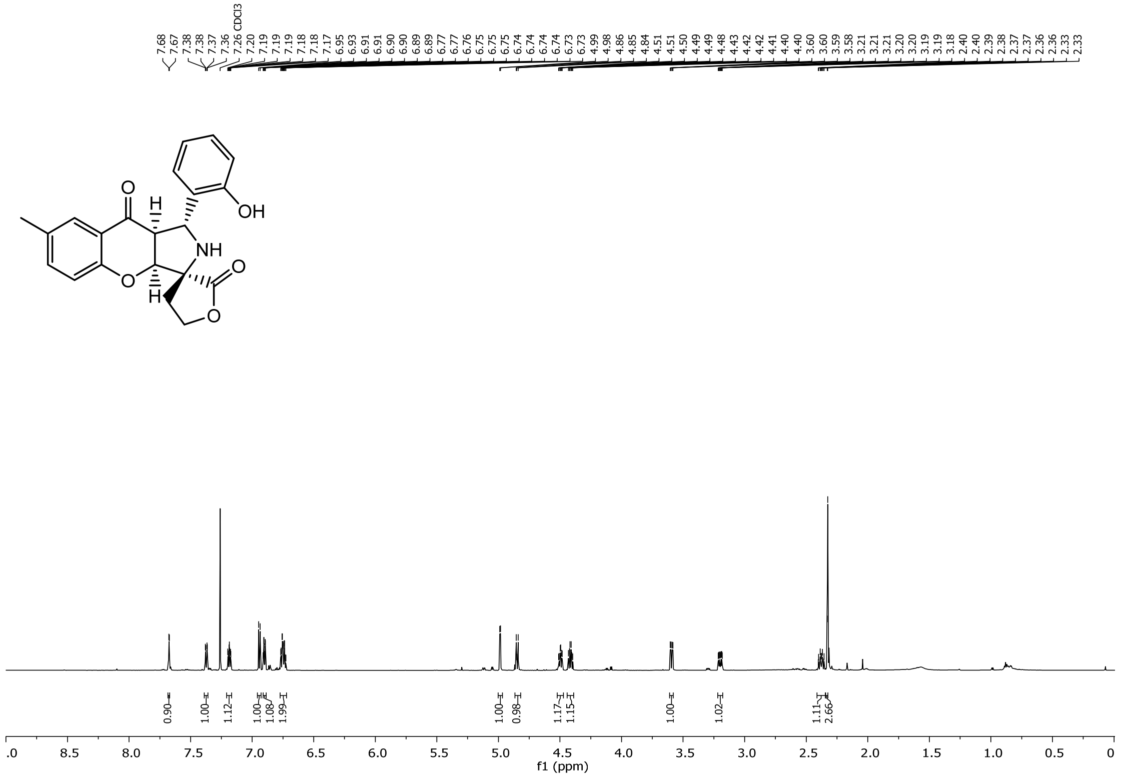

^{13}C NMR

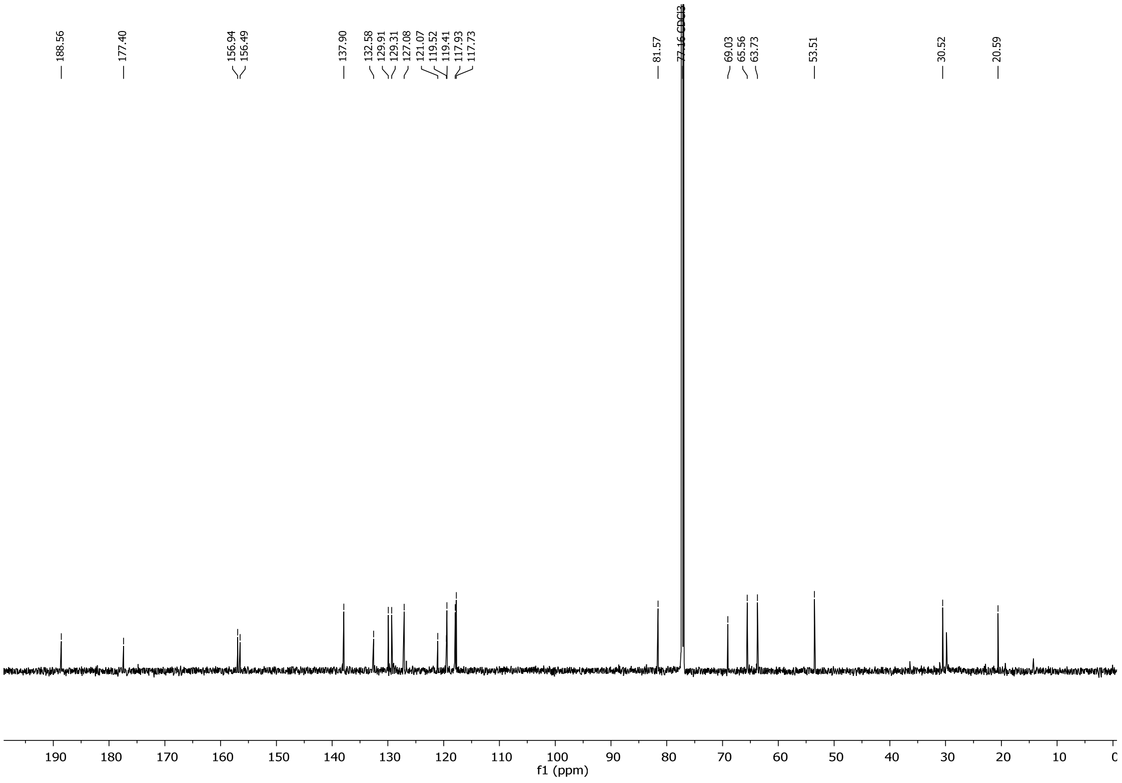

(1*R*,3*R*,3a*S*,9a*S*)-1-(2-Hydroxyphenyl)-6-methyl-3a,4',5',9a-tetrahydro-1*H*,2'*H*-spiro[chromeno[2,3-*c*]pyrrole-3,3'-furan]-2',9(2*H*)-dione 1o

^{1}H NMR

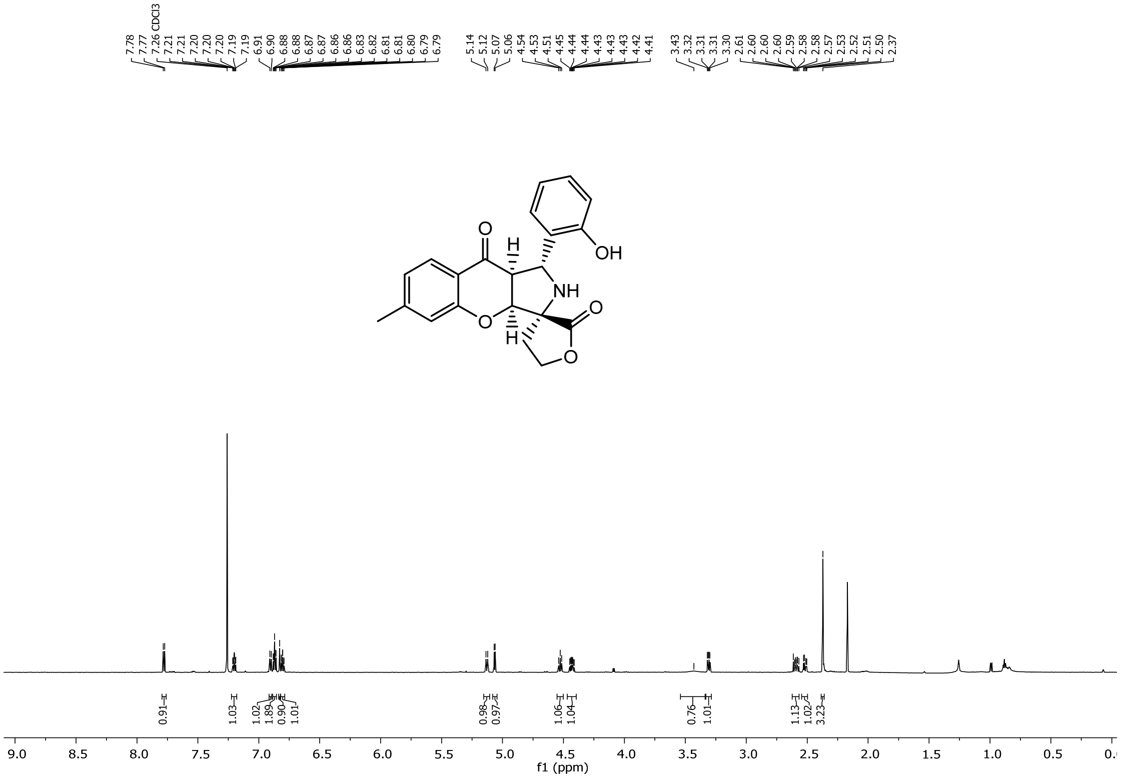

^{13}C NMR

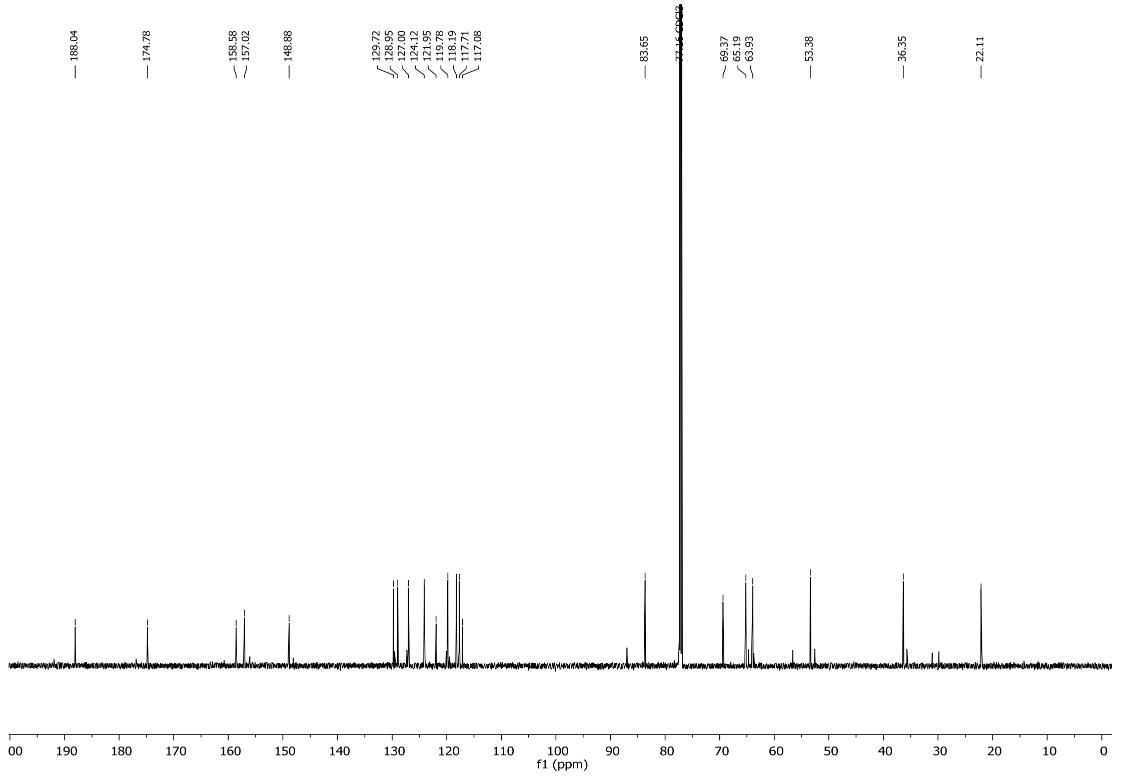

(1*R*,3*S*,3a*S*,9a*S*)-1-(2-Hydroxyphenyl)-6-methyl-3a,4',5',9a-tetrahydro-1*H*,2'*H*-spiro[chromeno[2,3-*c*]pyrrole-3,3'-furan]-2',9(2*H*)-dione 1o'

^{1}H NMR

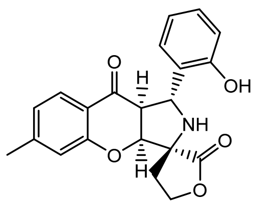

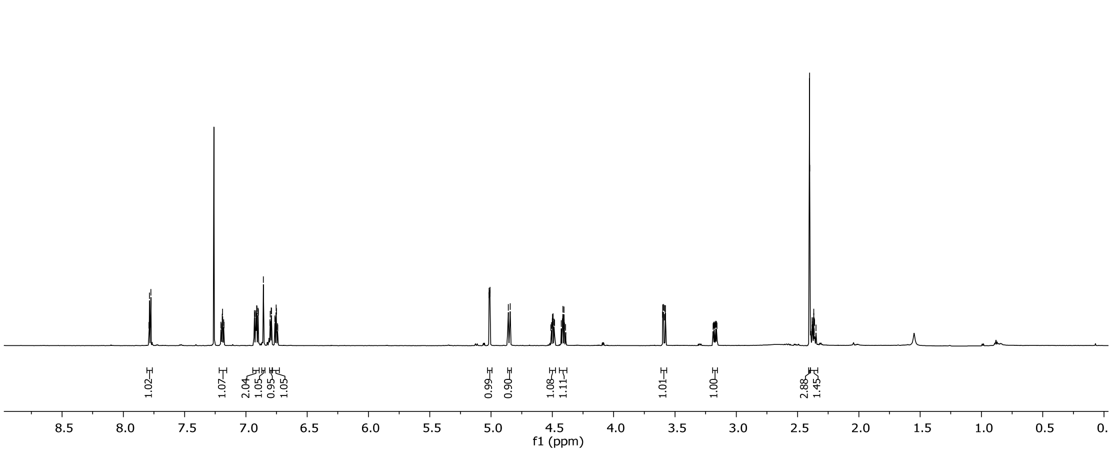

^{13}C NMR

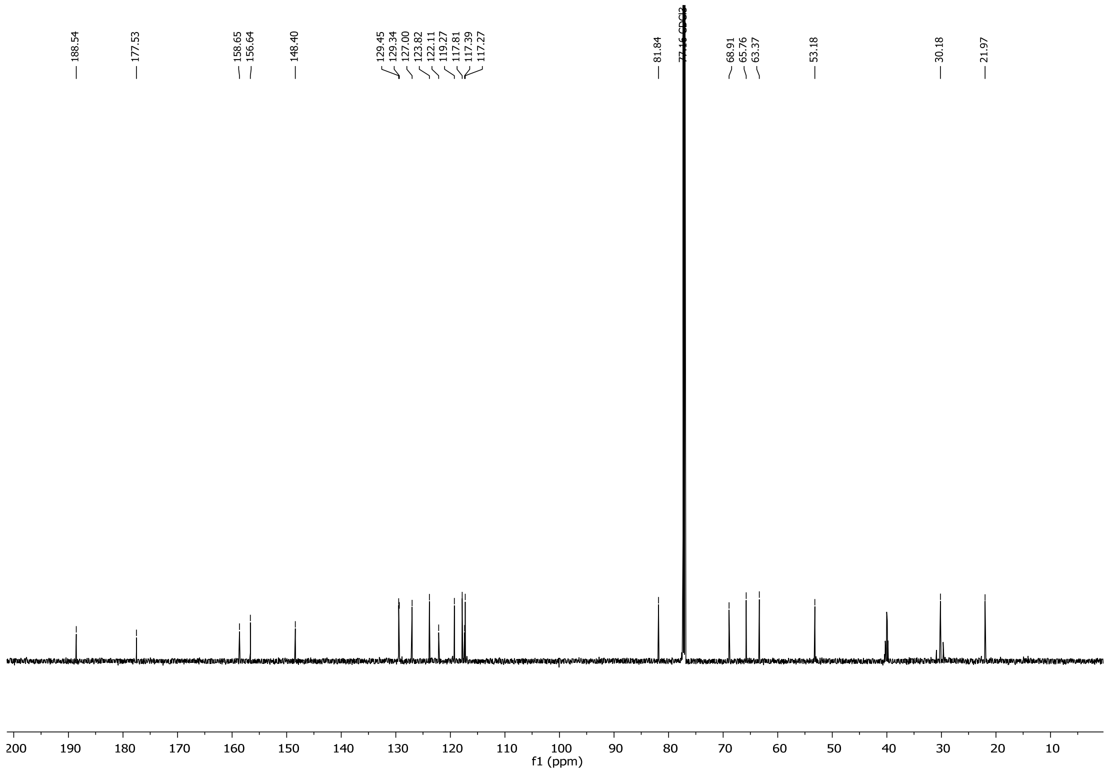

(1*R*,3*R*,3a*S*,9a*S*)-7-Chloro-1-(2-hydroxyphenyl)-3a,4',5',9a-tetrahydro-1*H*,2'*H*-spiro[chromeno[2,3-*c*]pyrrole-3,3'-furan]-2',9(2*H*)-dione 1p

^{1}H NMR

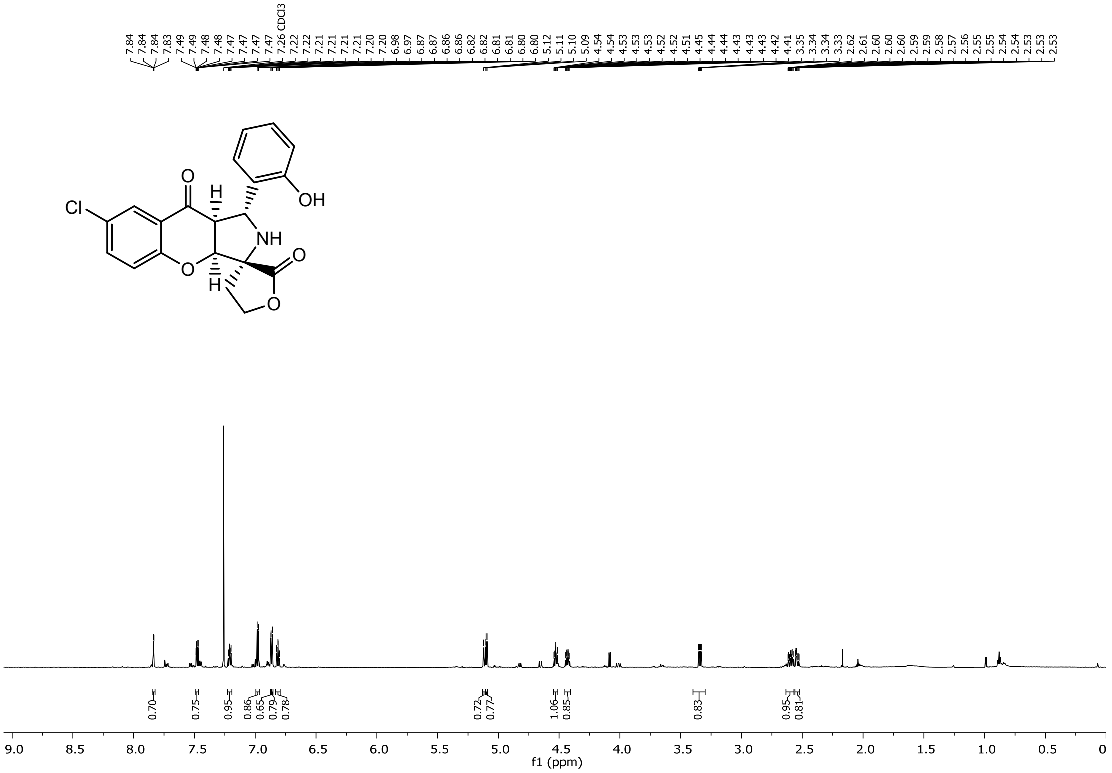

^{13}C NMR

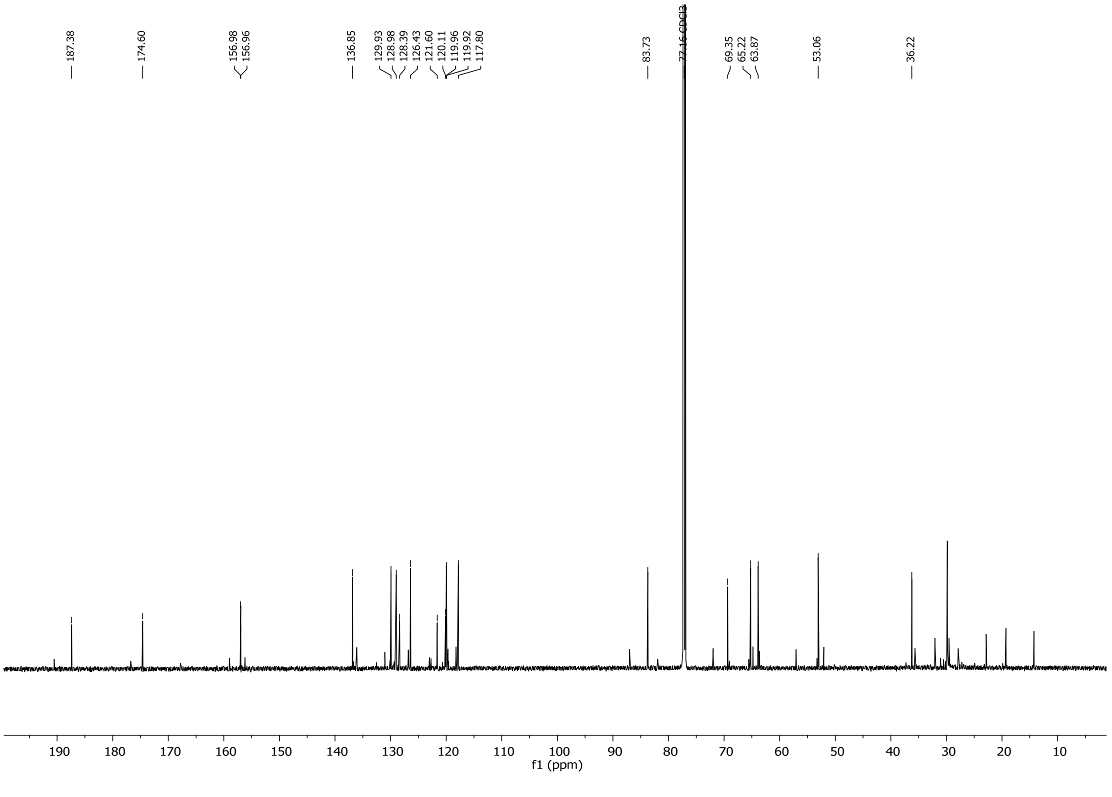

(1*R*,3*S*,3a*S*,9a*S*)-7-Chloro-1-(2-hydroxyphenyl)-3a,4',5',9a-tetrahydro-1*H*,2'*H*-spiro[chromeno[2,3-*c*]pyrrole-3,3'-furan]-2',9(2*H*)-dione 1p'

^{1}H NMR

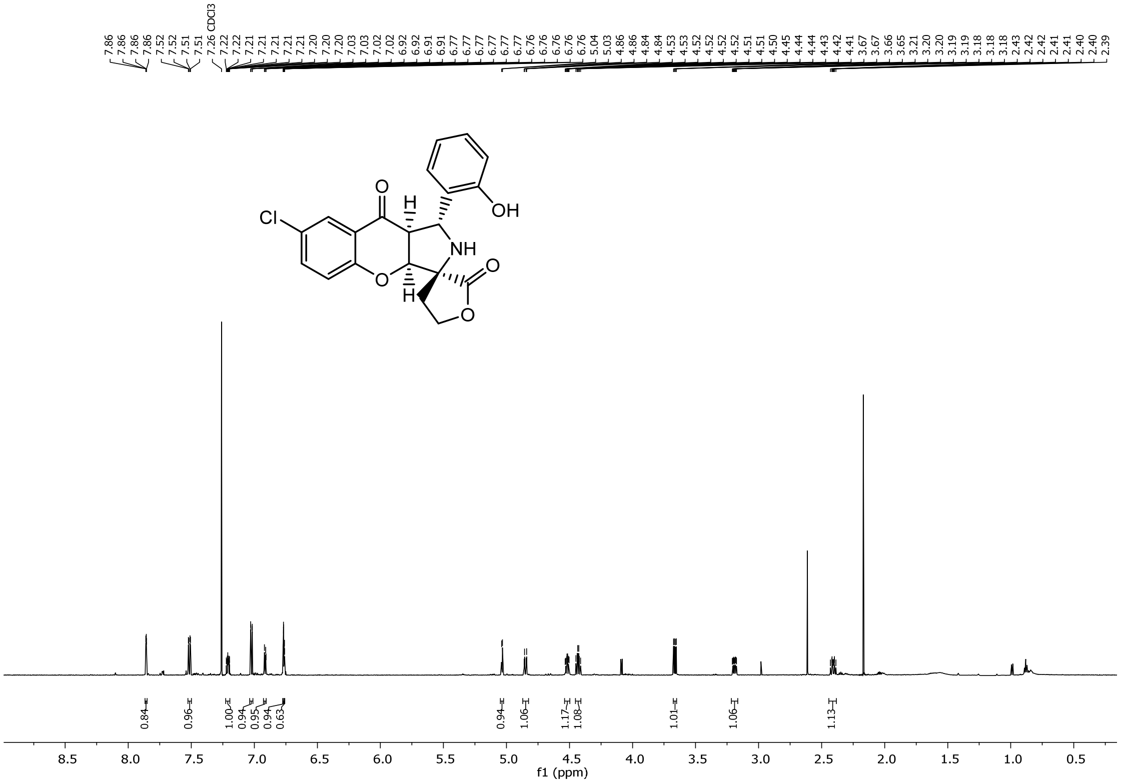

^{13}C NMR

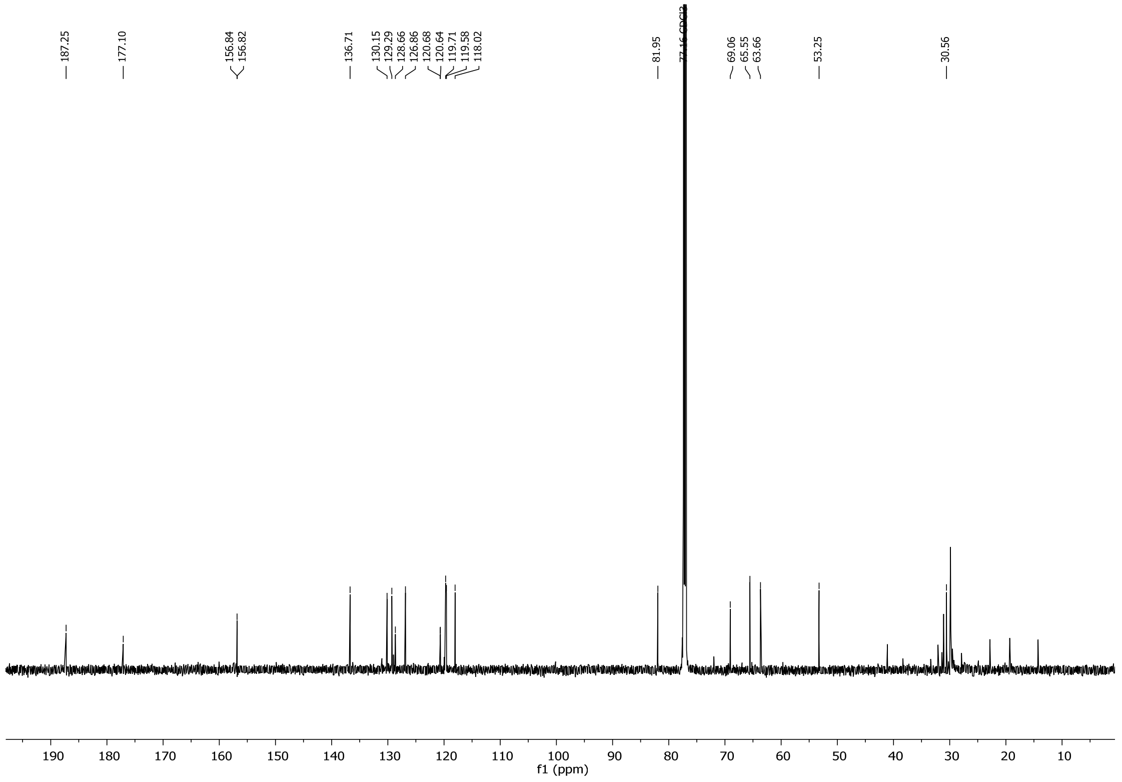

(1*R*,3*R*,3a*S*,9a*S*)-6-Fluoro-1-(2-hydroxyphenyl)-3a,4',5',9a-tetrahydro-1*H*,2'*H*-spiro[chromeno[2,3-*c*]pyrrole-3,3'-furan]-2',9(2*H*)-dione 1r

^{1}H NMR

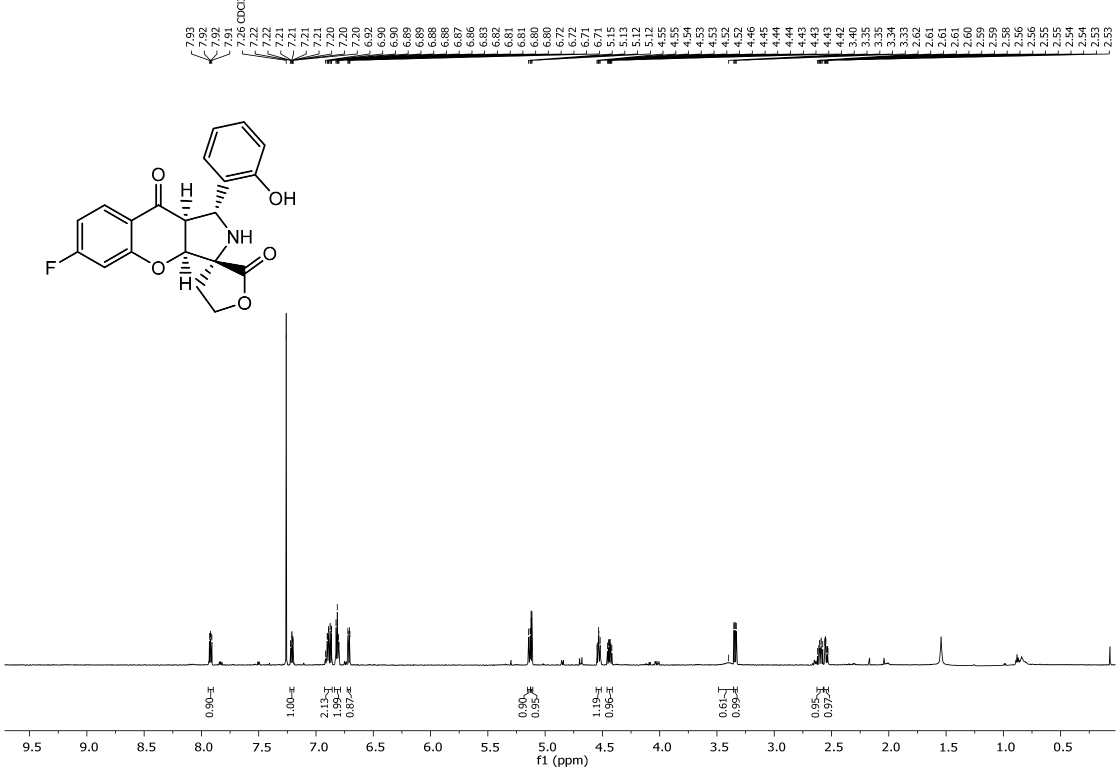

^{13}C NMR

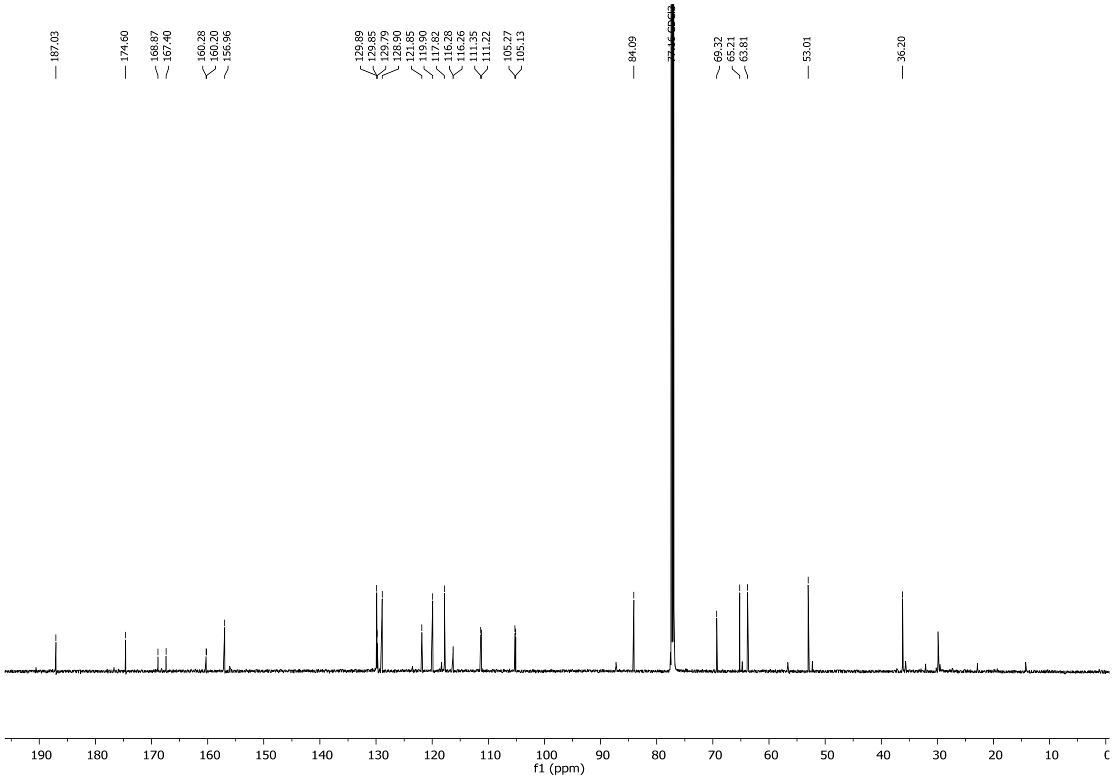

(1*R*,3*S*,3a*S*,9a*S*)-6-Fluoro-1-(2-hydroxyphenyl)-3a,4',5',9a-tetrahydro-1*H*,2'*H*-spiro[chromeno[2,3-*c*]pyrrole-3,3'-furan]-2',9(2*H*)-dione 1r′

^{1}H NMR

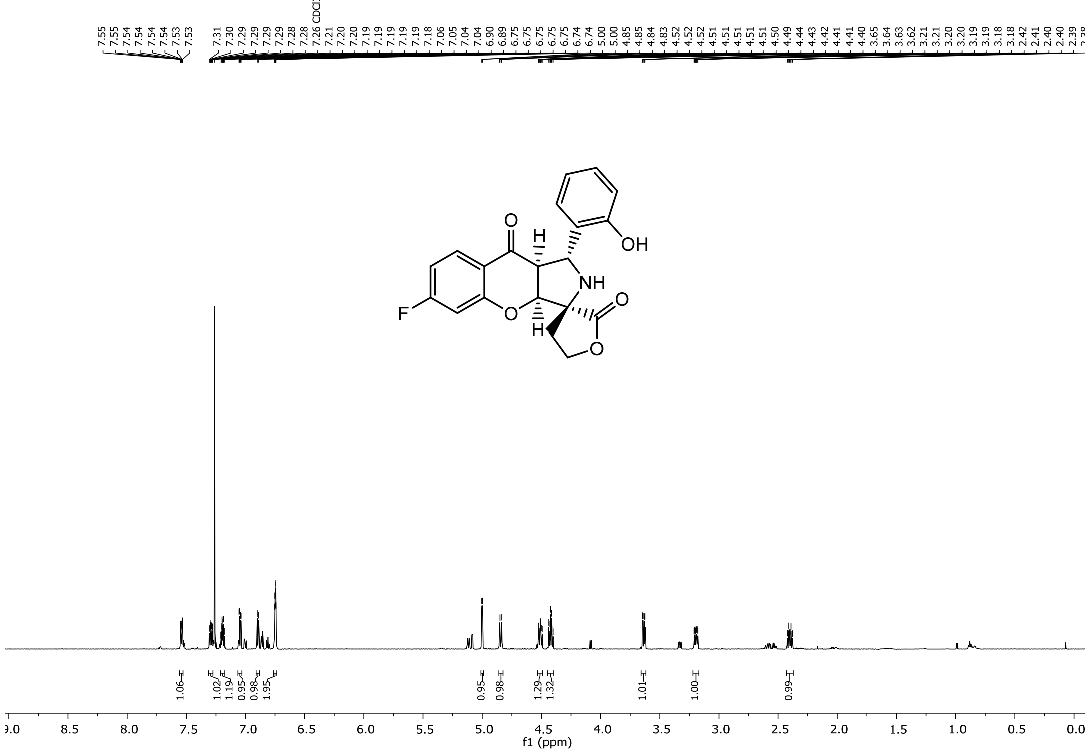

^{13}C NMR

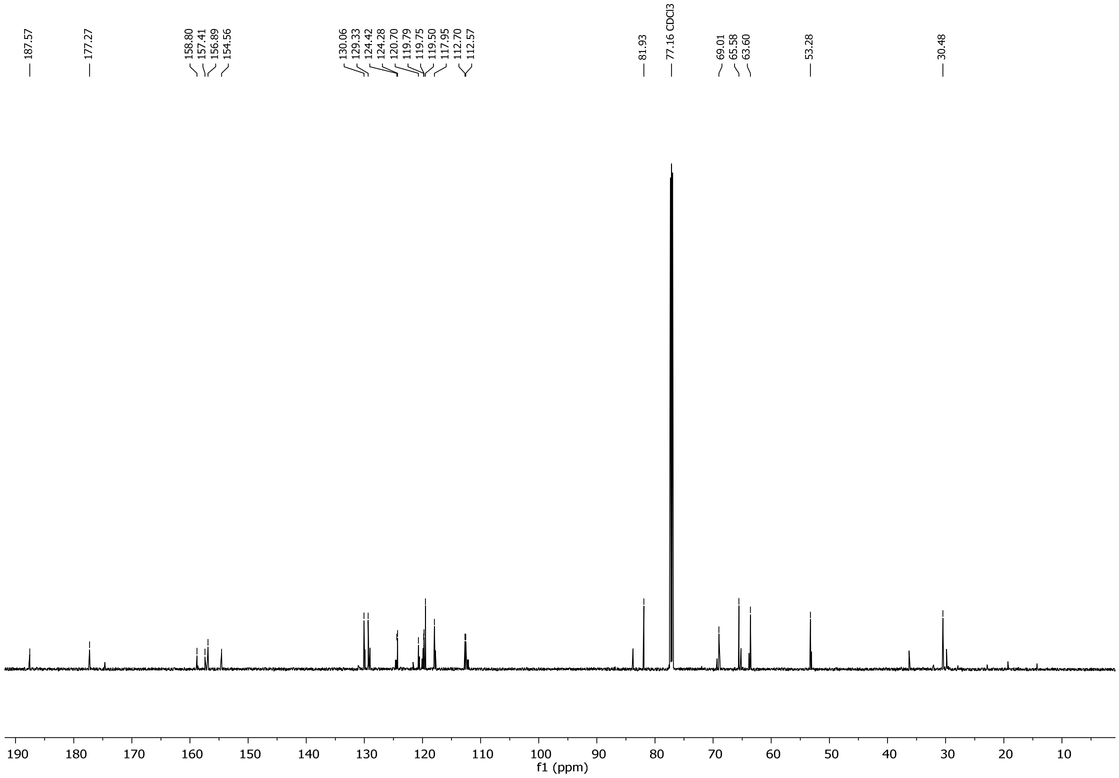

(1*R*,3*R*,3a*S*,9a*S*)-7-Chloro-1-(2-hydroxyphenyl)-6-methyl-3a,4',5',9a-tetrahydro-1*H*,2'*H*-spiro[chromeno[2,3-*c*]pyrrole-3,3'-furan]-2',9(2*H*)-dione 1s

^{1}H NMR

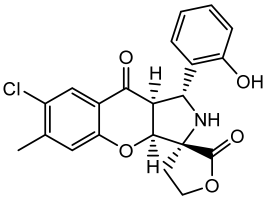

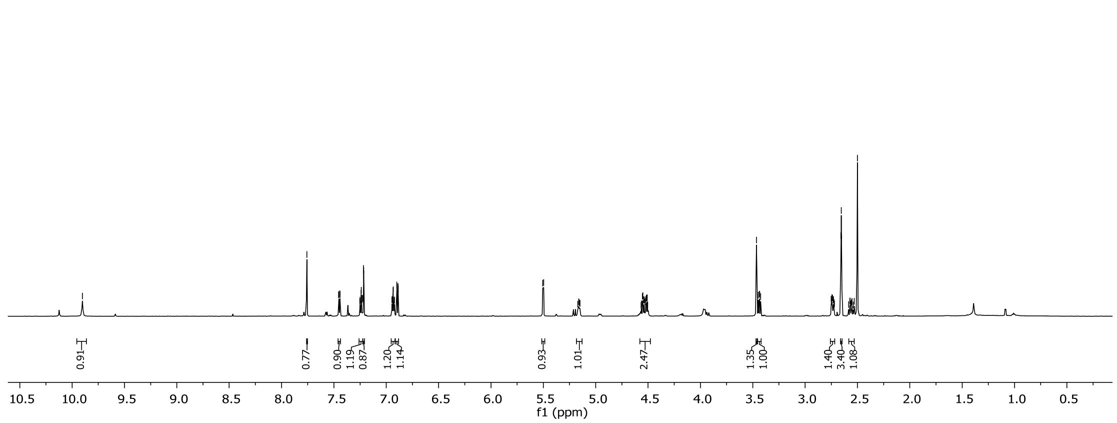

^{13}C NMR

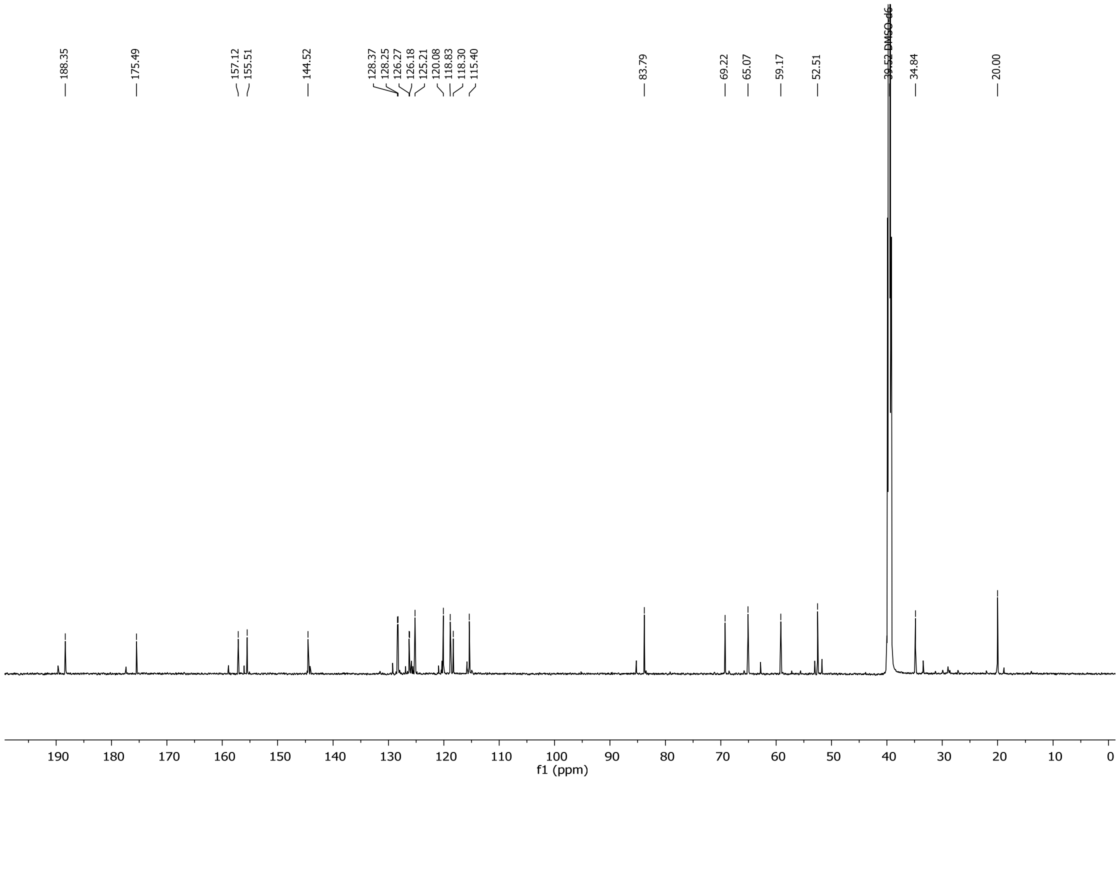

(1*R*,3*S*,3a*S*,9a*S*)-7-Chloro-1-(2-hydroxyphenyl)-6-methyl-3a,4',5',9a-tetrahydro-1*H*,2'*H*-spiro[chromeno[2,3-*c*]pyrrole-3,3'-furan]-2',9(2*H*)-dione 1s'

^{1}H NMR

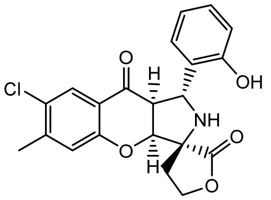

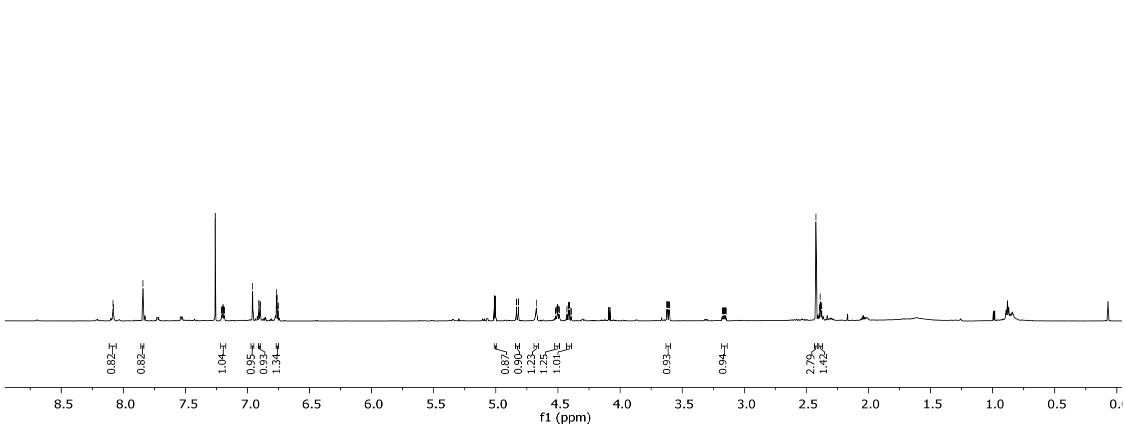

^{13}C NMR

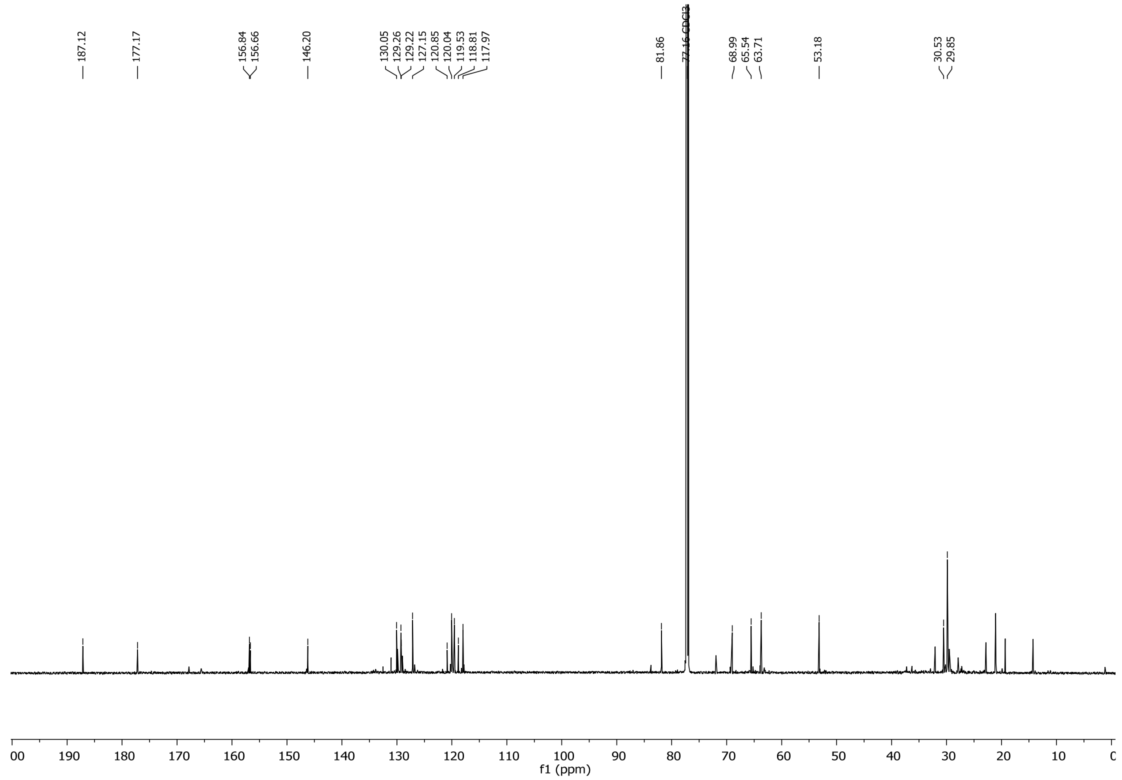

(1*R*,3*R*,3a*S*,9a*S*)-1-(2-Hydroxy-5-methylphenyl)-3a,4',5',9a-tetrahydro-1*H*,2'*H*-spiro[chromeno[2,3-*c*]pyrrole-3,3'-furan]-2',9(2*H*)-dione 1t

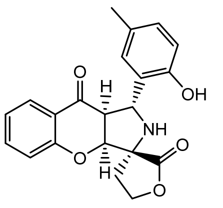

^{1}H NMR

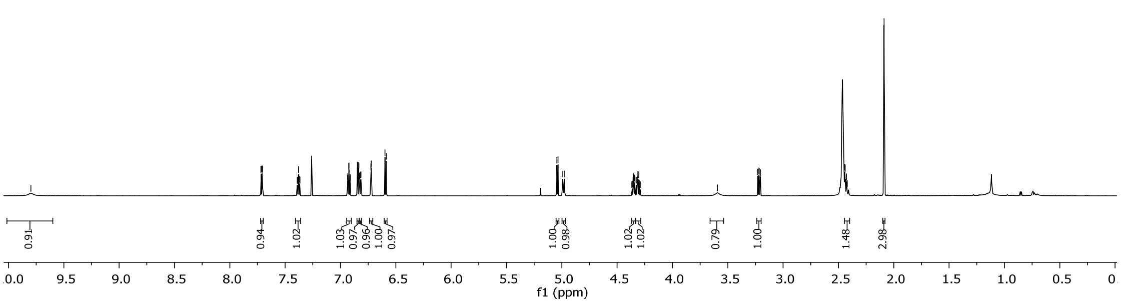

^{13}C NMR

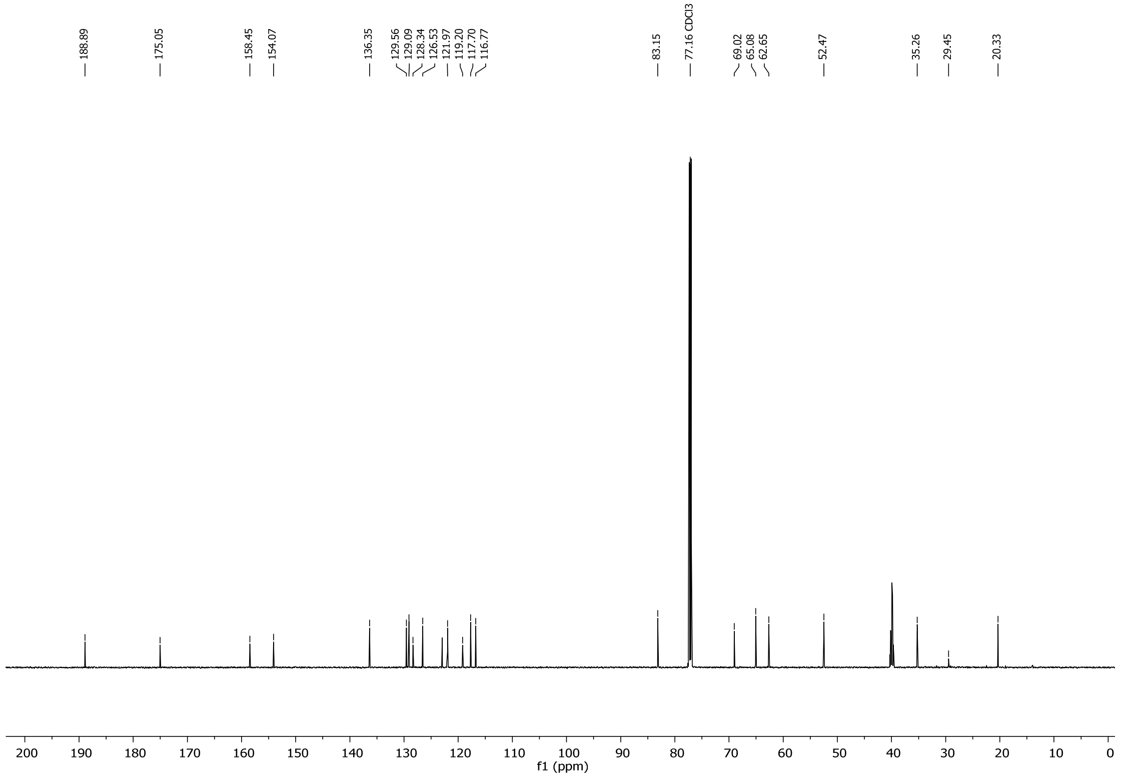

(1*R*,3*S*,3a*S*,9a*S*)-1-(2-Hydroxy-5-methylphenyl)-3a,4',5',9a-tetrahydro-1*H*,2'*H*-spiro[chromeno[2,3-*c*]pyrrole-3,3'-furan]-2',9(2*H*)-dione 1t'

^{1}H NMR

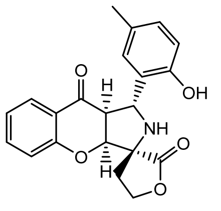

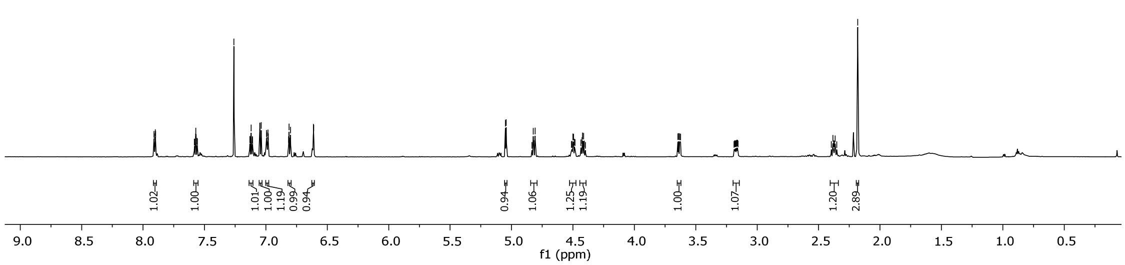

^{13}C NMR

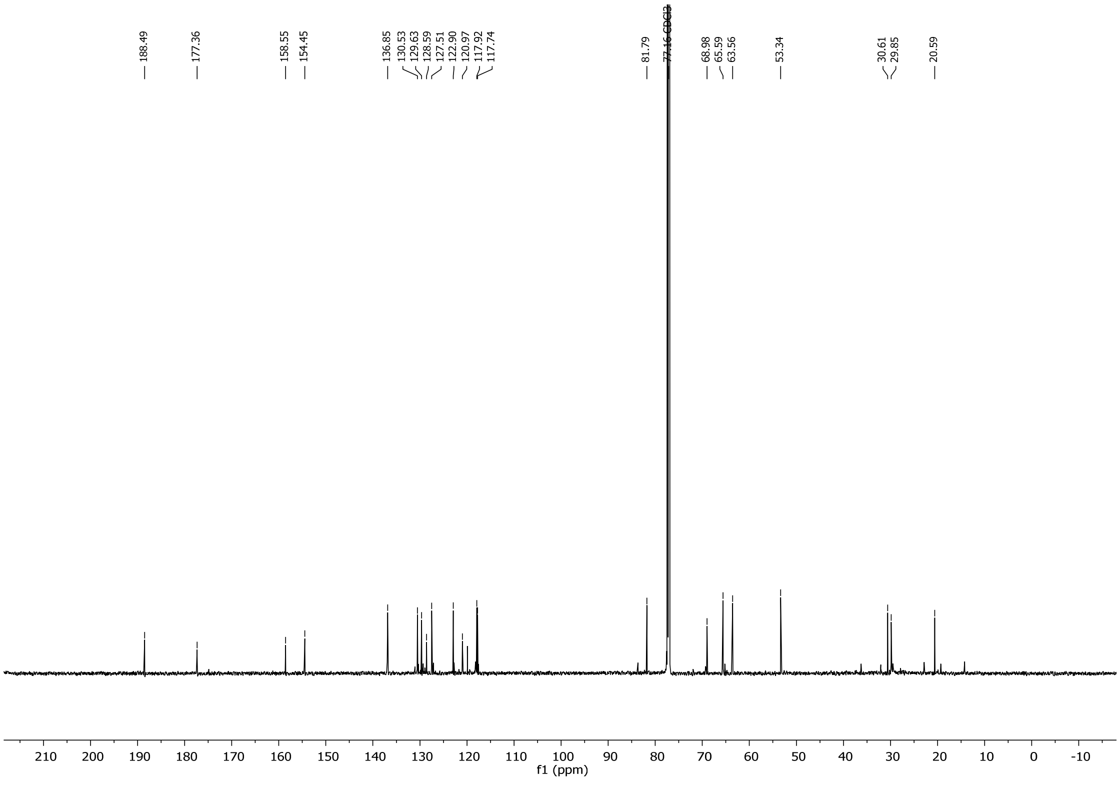

(1*R*,3*R*,3a*S*,9a*S*)-1-(5-Bromo-2-hydroxyphenyl)-3a,4',5',9a-tetrahydro-1*H*,2'*H*-spiro[chromeno[2,3-*c*]pyrrole-3,3'-furan]-2',9(2*H*)-dione 1u

^{1}H NMR

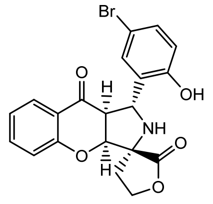

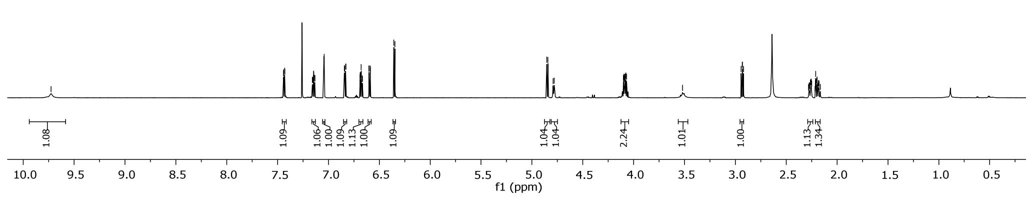

^{13}C NMR

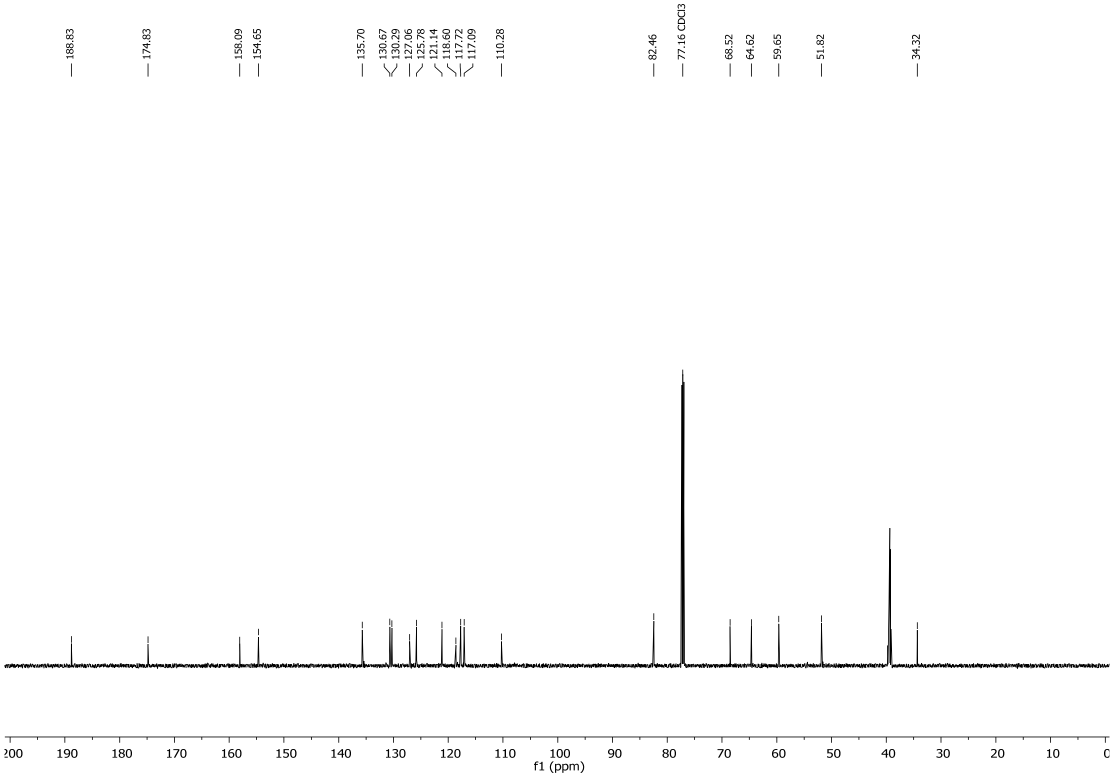

(1*R*,3*S*,3a*S*,9a*S*)-1-(5-Bromo-2-hydroxyphenyl)-3a,4',5',9a-tetrahydro-1*H*,2'*H*-spiro[chromeno[2,3-*c*]pyrrole-3,3'-furan]-2',9(2*H*)-dione 1u'

^{1}H NMR

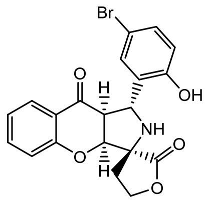

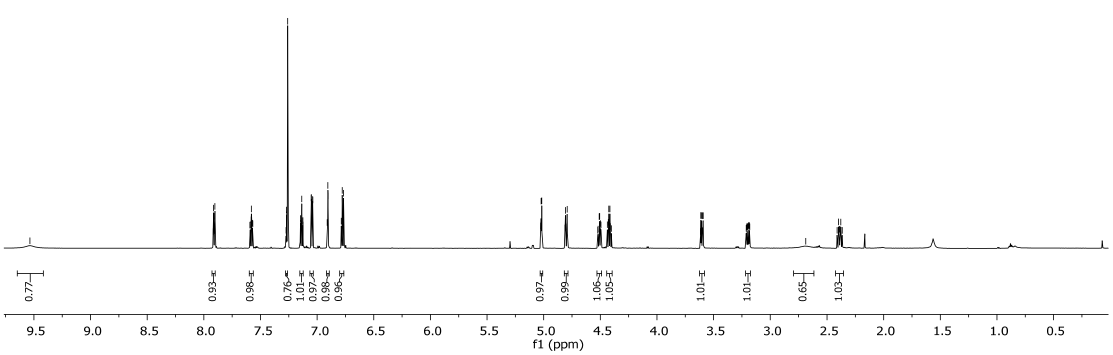

^{13}C NMR

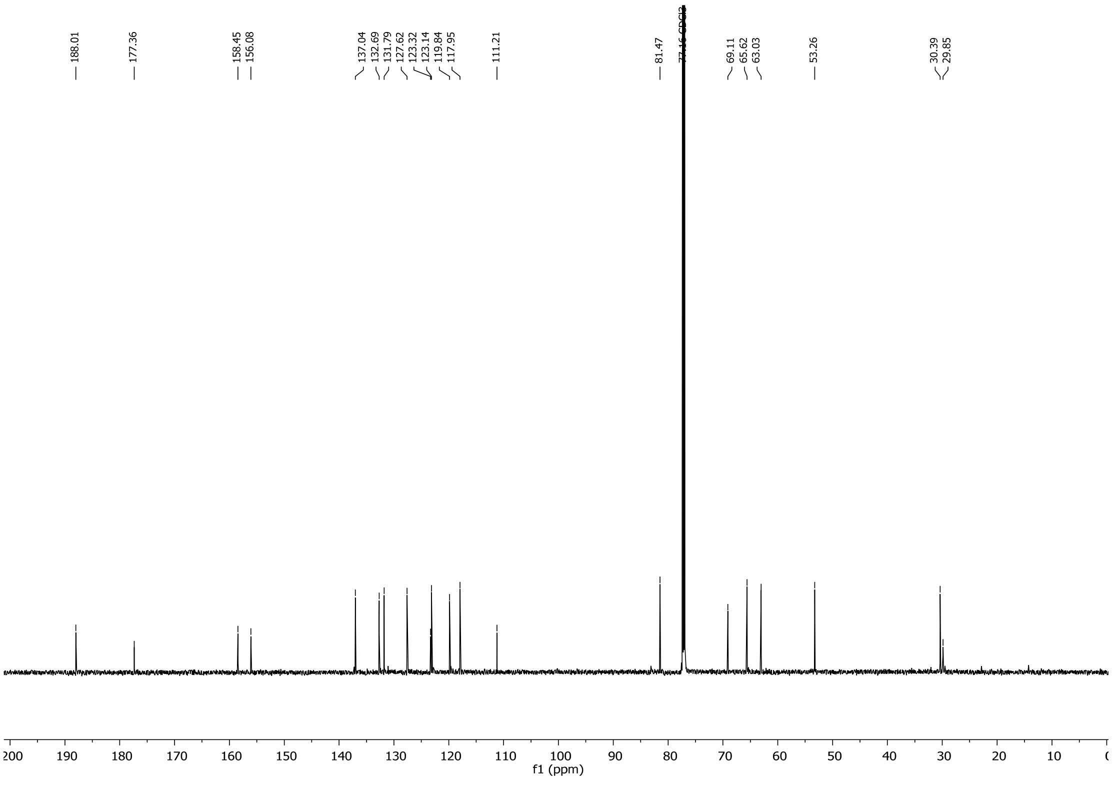

(1*R*,3*R*,3a*S*,9a*S*)-1-(5-Chloro-2-hydroxyphenyl)-3a,4',5',9a-tetrahydro-1*H*,2'*H*-spiro[chromeno[2,3-*c*]pyrrole-3,3'-furan]-2',9(2*H*)-dione 1w

^{1}H NMR

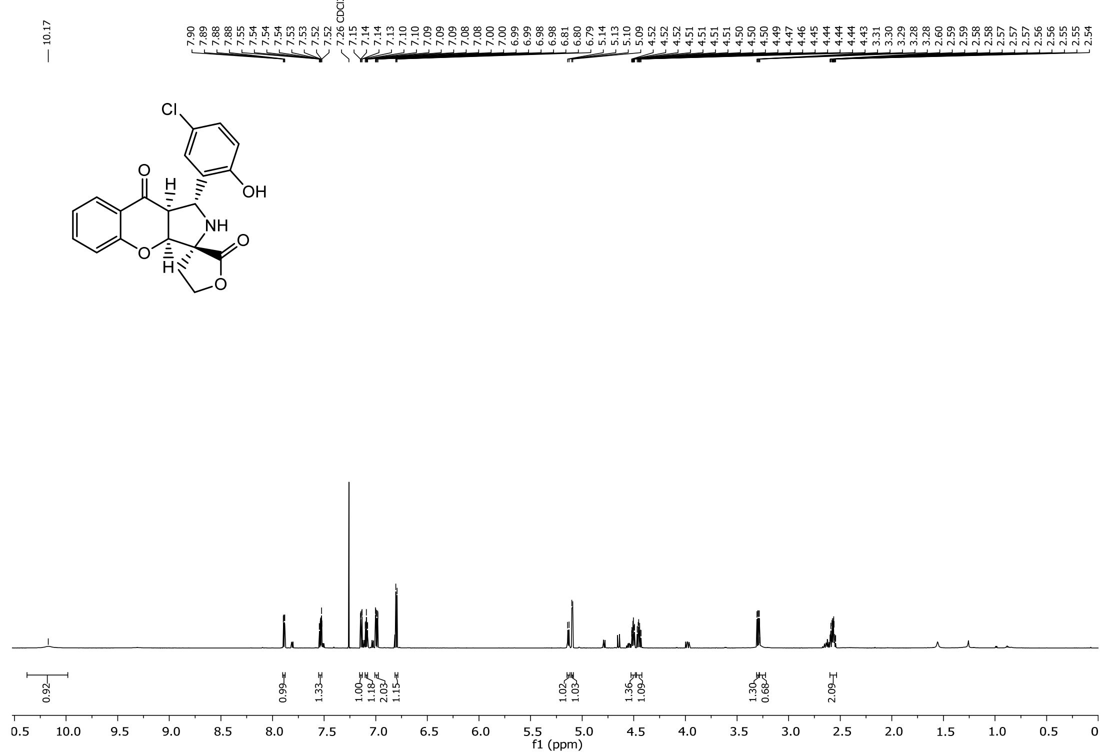

^{13}C NMR

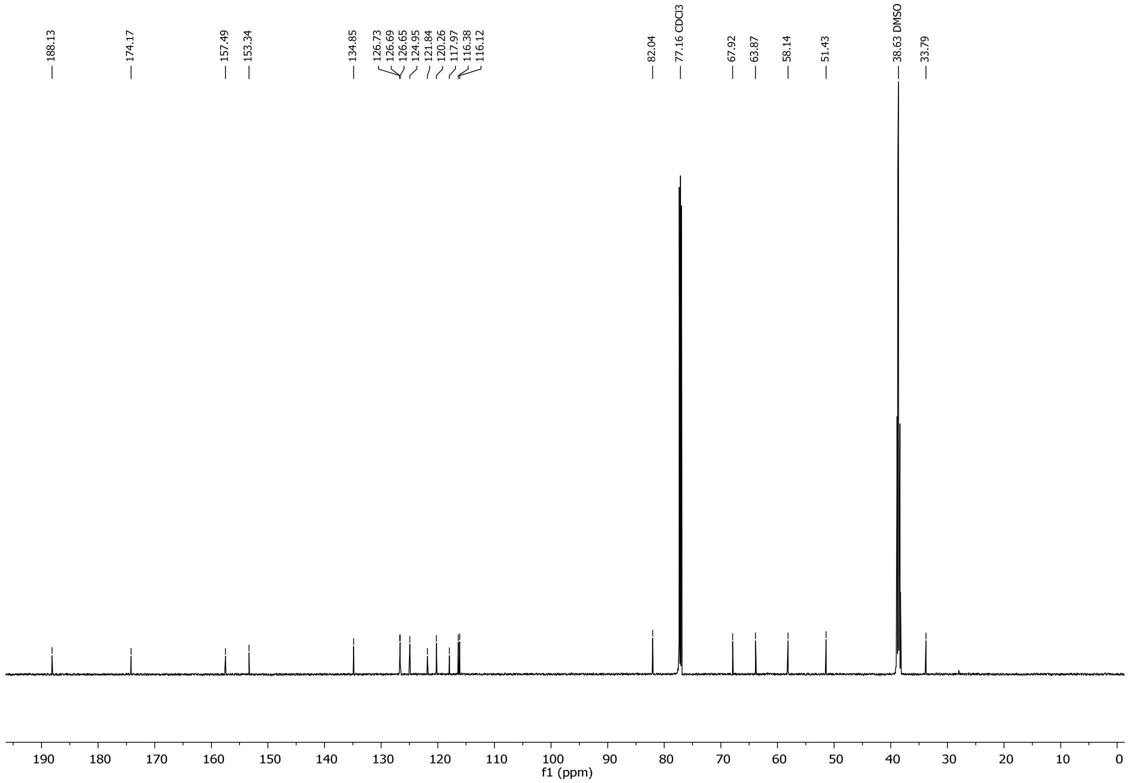

(1*R*,3*S*,3a*S*,9a*S*)-1-(5-Chloro-2-hydroxyphenyl)-3a,4',5',9a-tetrahydro-1*H*,2'*H*-spiro[chromeno[2,3-*c*]pyrrole-3,3'-furan]-2',9(2*H*)-dione 1w’

^{1}H NMR

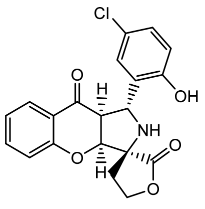

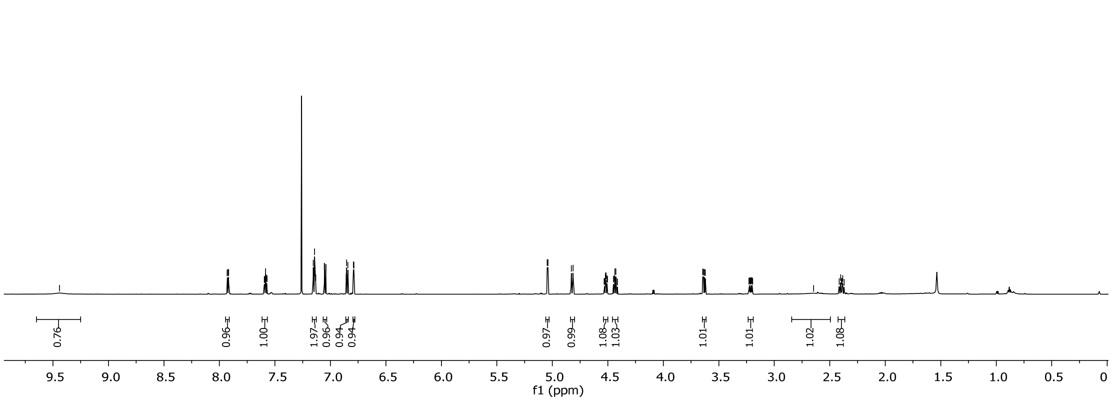

^{13}C NMR

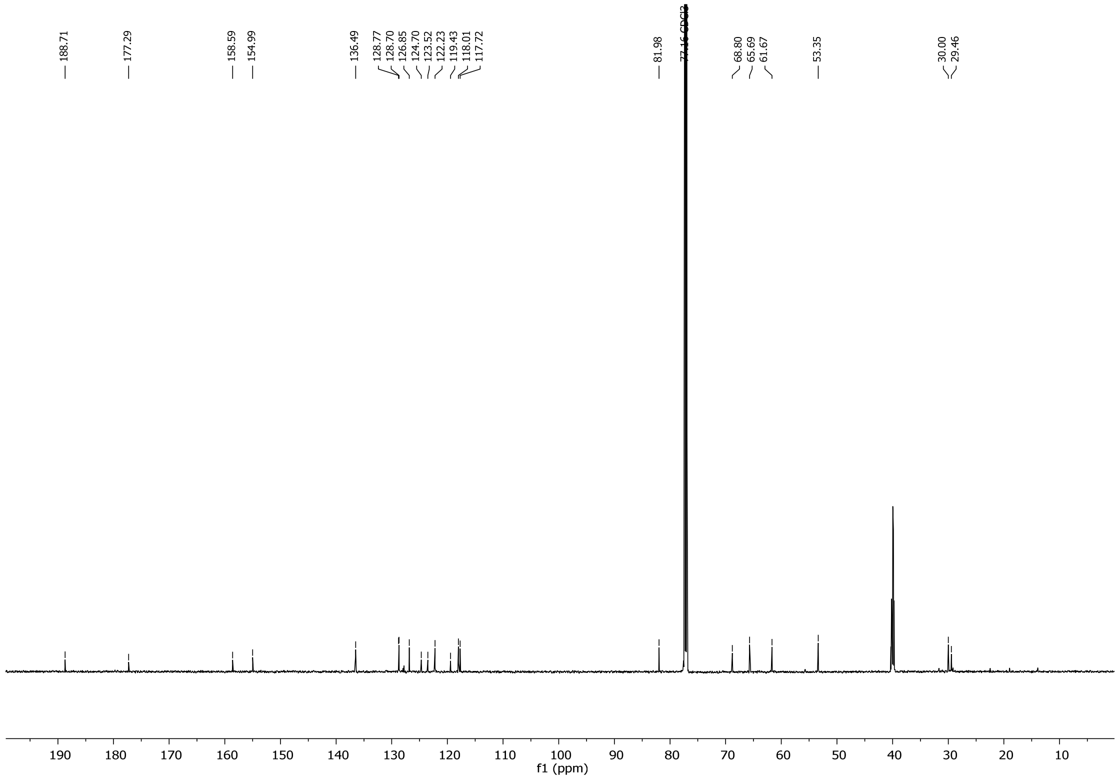

(1*R*,3*R*,3a*S*,9a*S*)-1-(2-Hydroxy-5-nitrophenyl)-3a,4',5',9a-tetrahydro-1*H*,2'*H*-spiro[chromeno[2,3-*c*]pyrrole-3,3'-furan]-2',9(2*H*)-dione 1x

^{1}H NMR

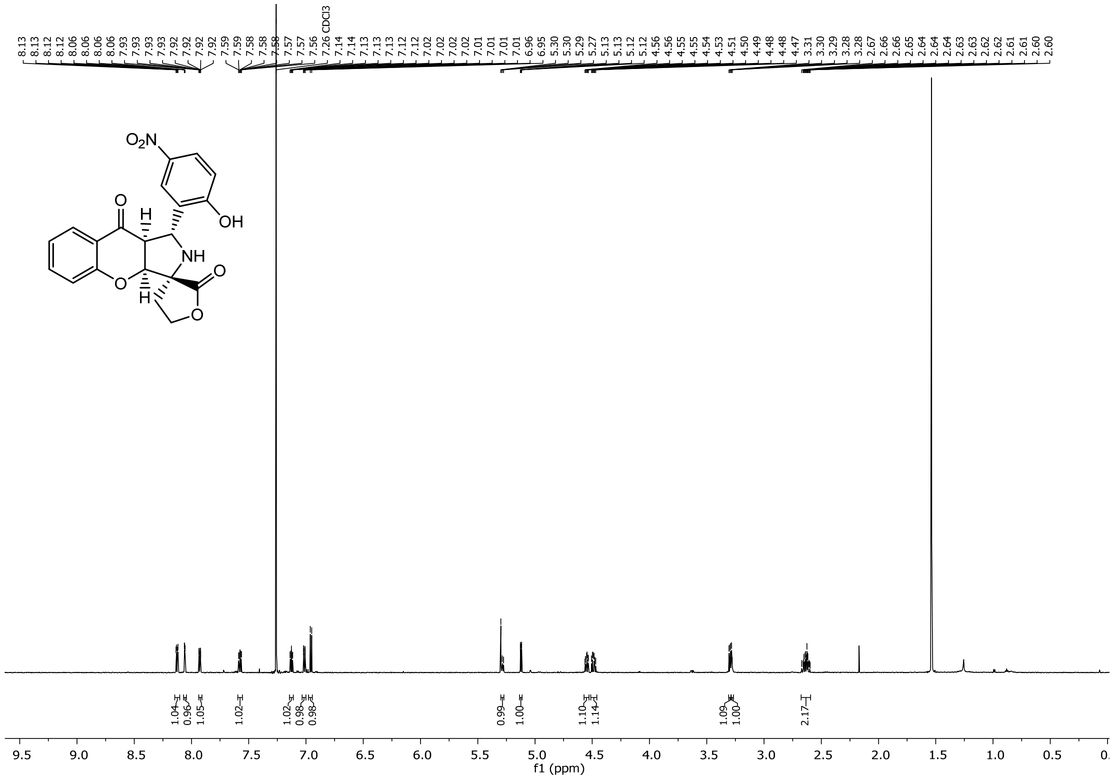

^{13}C NMR

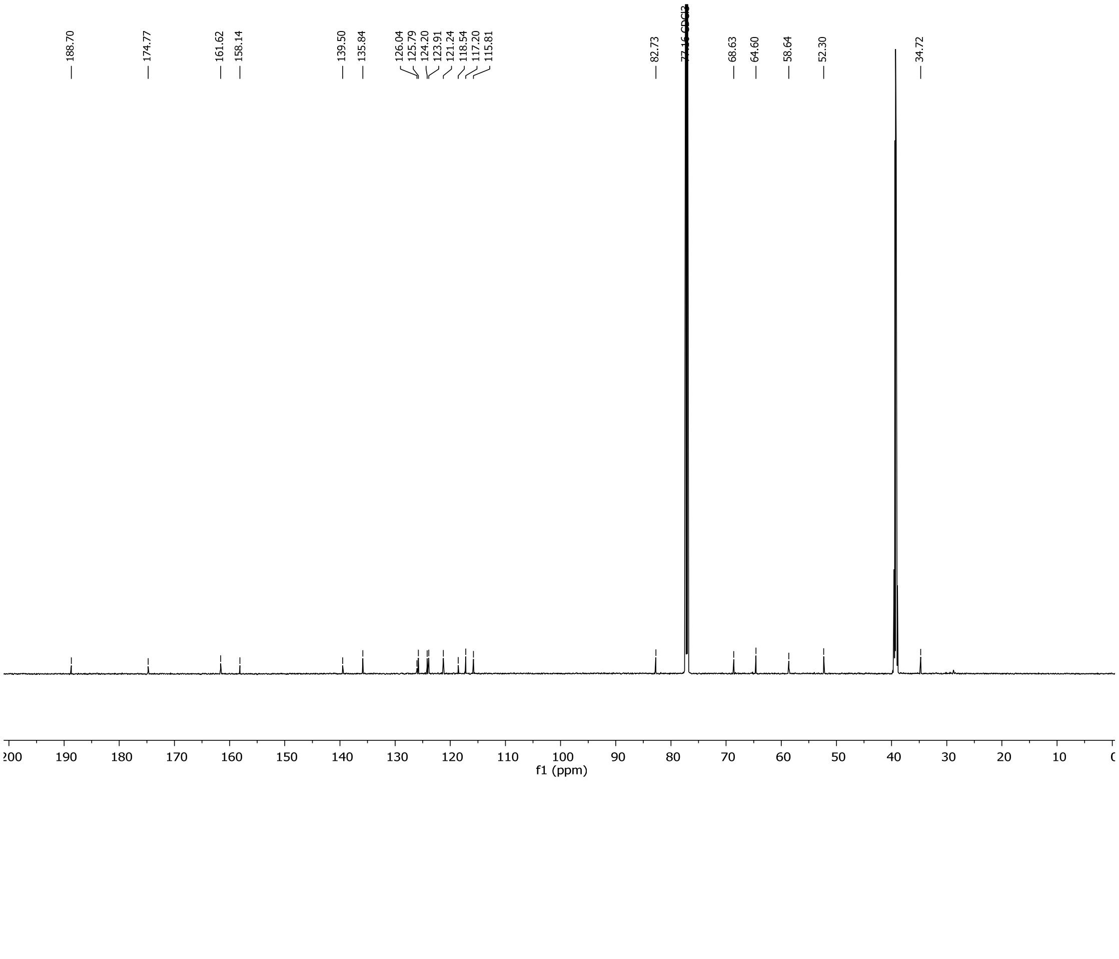

(1*R*,3*S*,3a*S*,9a*S*)-1-(2-Hydroxy-5-nitrophenyl)-3a,4',5',9a-tetrahydro-1*H*,2'*H*-spiro[chromeno[2,3-*c*]pyrrole-3,3'-furan]-2',9(2*H*)-dione 1x'

^{1}H NMR

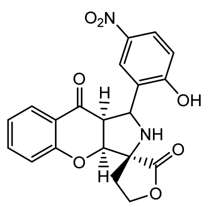

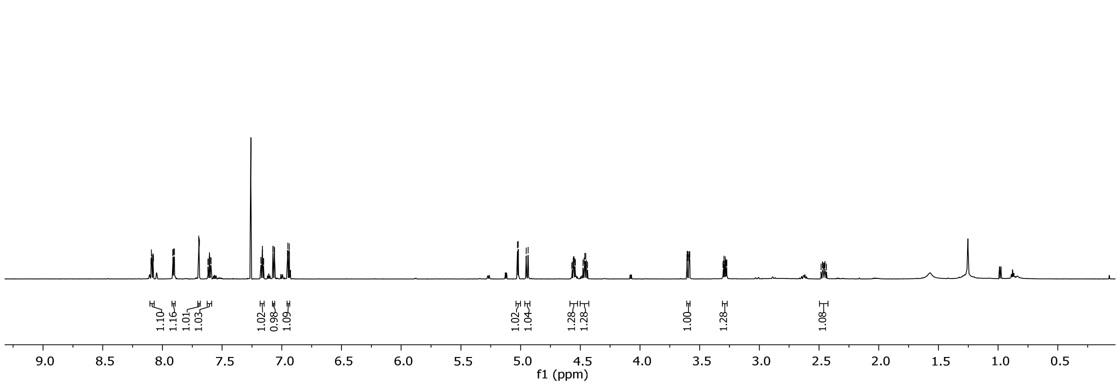

^{13}C NMR

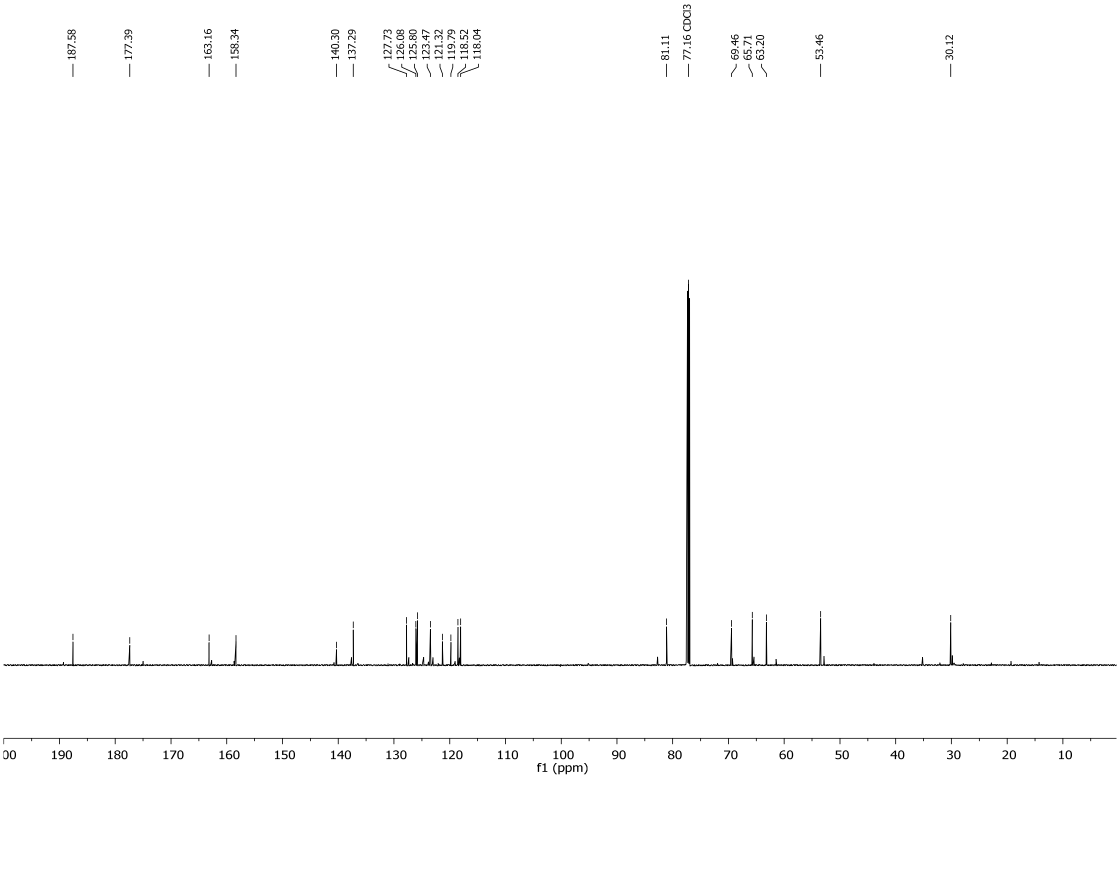

(8a*S*,14a*S*,14b*R*)-Diethyl 2-(*tert*-butyl)-14-oxo-8a,14,14a,14b-tetrahydrobenzo-[*e*]chromeno[3',2':3,4]pyrrolo[1,2-*c*][1,3]oxazine-8,8(6*H*)-dicarboxylate 9a

^{1}H NMR

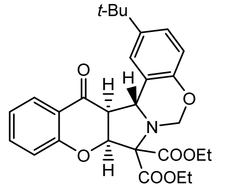

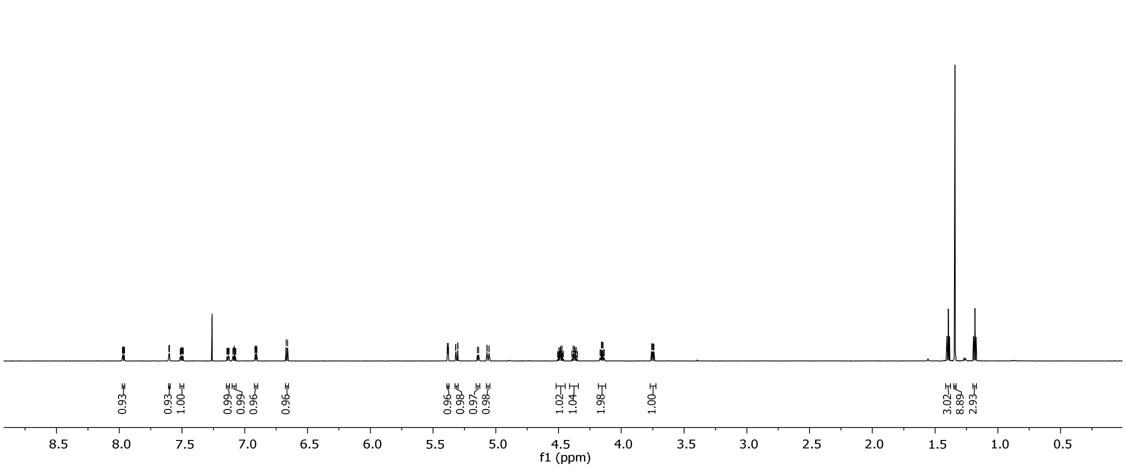

^{13}C NMR

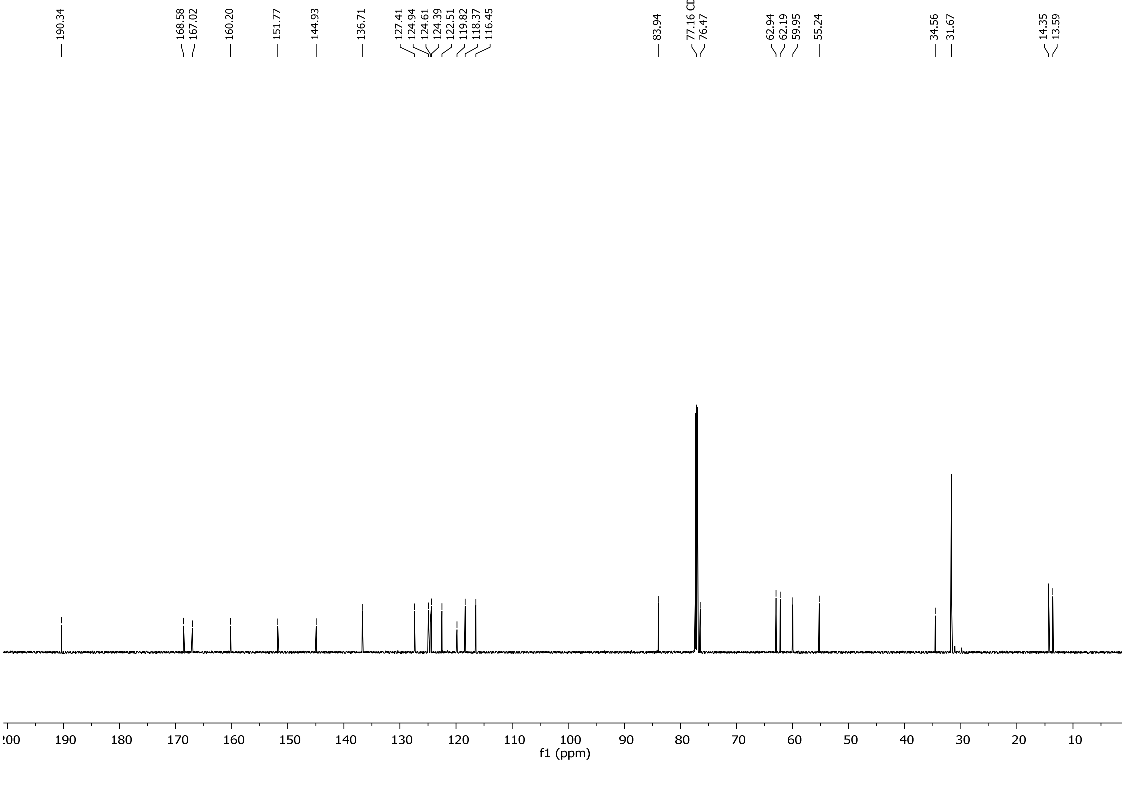

4. **HPLC data**

(1*R*,3a*S*,9a*S*)-Diethyl 1-(2-hydroxyphenyl)-9-oxo-1,2,9,9a-tetrahydrochromeno[2,3-*c*]pyrrole-3,3(3a*H*)-dicarboxylate 1a

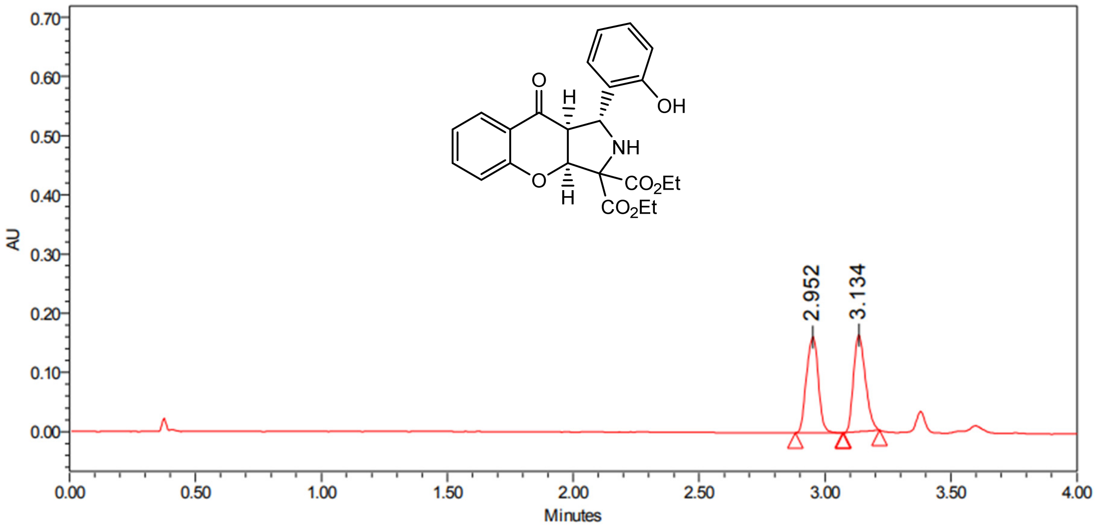

Peak Results

	RT	% Area
1	2.952	49.80
2	3.134	50.20

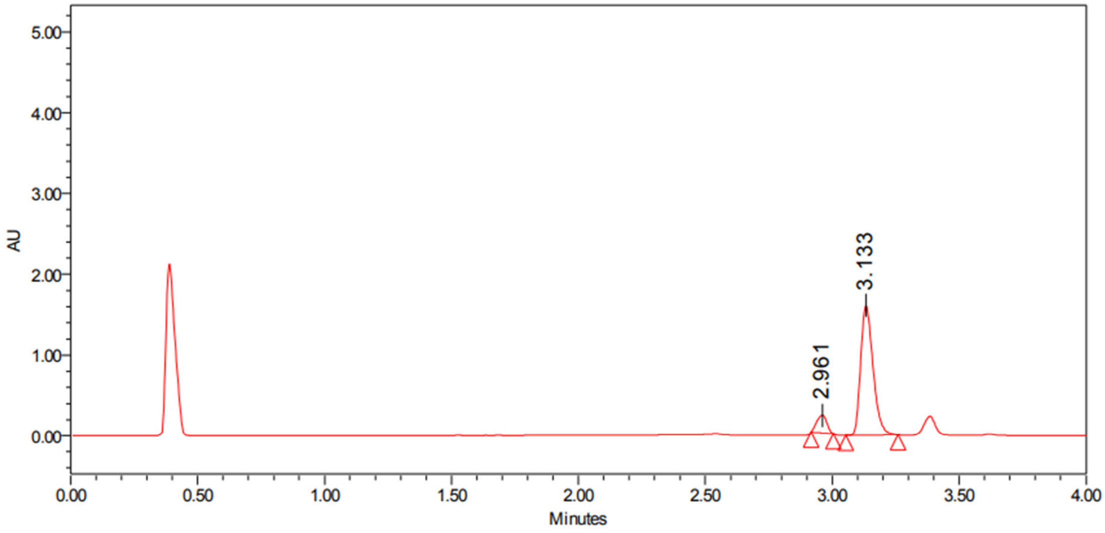

Peak Results

	RT	% Area
1	2.961	10.22
2	3.133	89.78

(1*R*,3a*S*,9a*S*)-Diethyl 1-(2-hydroxyphenyl)-7-methyl-9-oxo-1,2,9,9a-tetrahydrochromeno[2,3-*c*]pyrrole-3,3(3a*H*)-dicarboxylate 1b

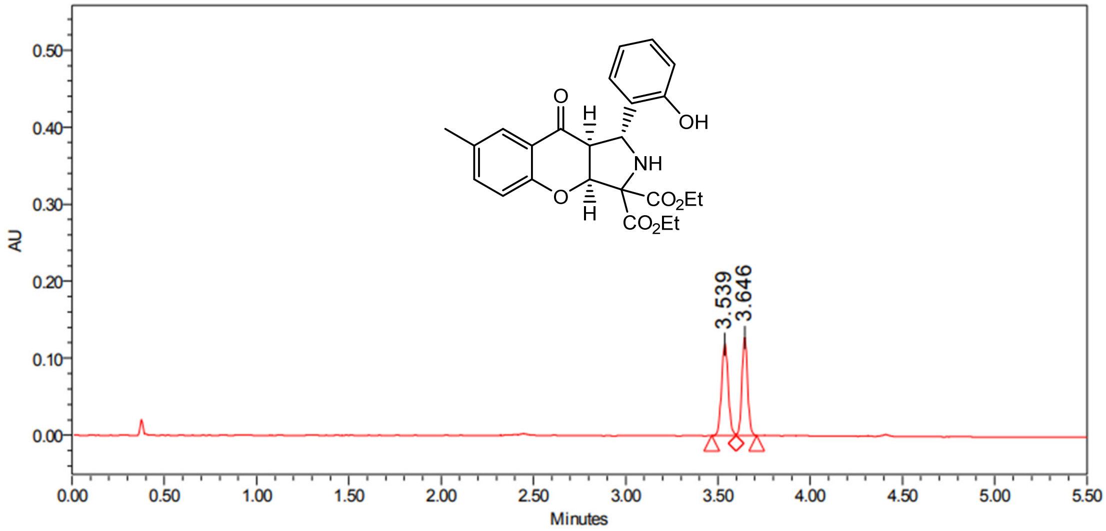

Peak Results

	RT	% Area
1	3.539	52.63
2	3.646	47.37

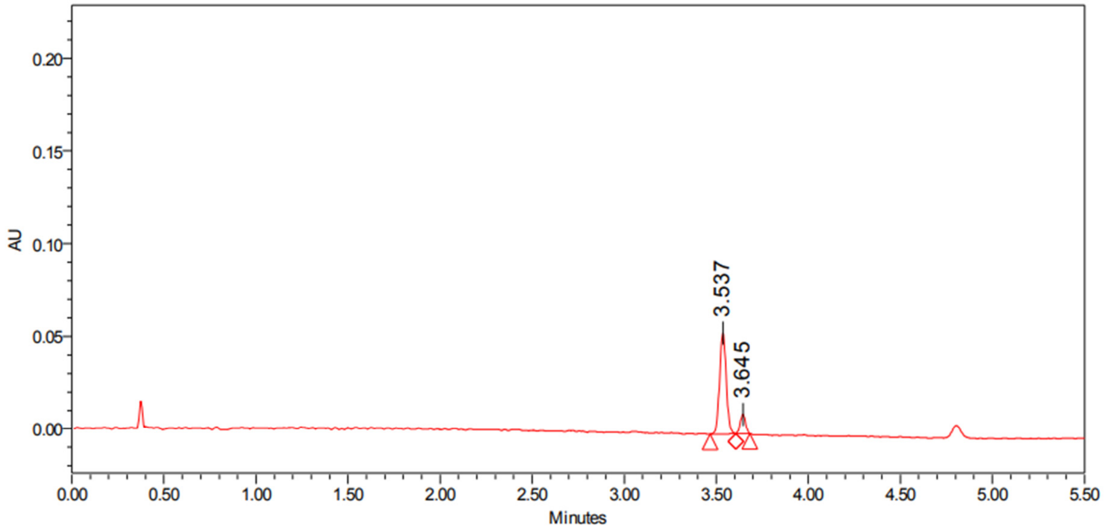

Peak Results

	RT	% Area
1	3.537	86.66
2	3.645	13.34

(1*R*,3a*S*,9a*S*)-Diethyl 1-(2-hydroxyphenyl)-6-methyl-9-oxo-1,2,9,9a-tetrahydrochromeno[2,3-*c*]pyrrole-3,3(3a*H*)-dicarboxylate 1c

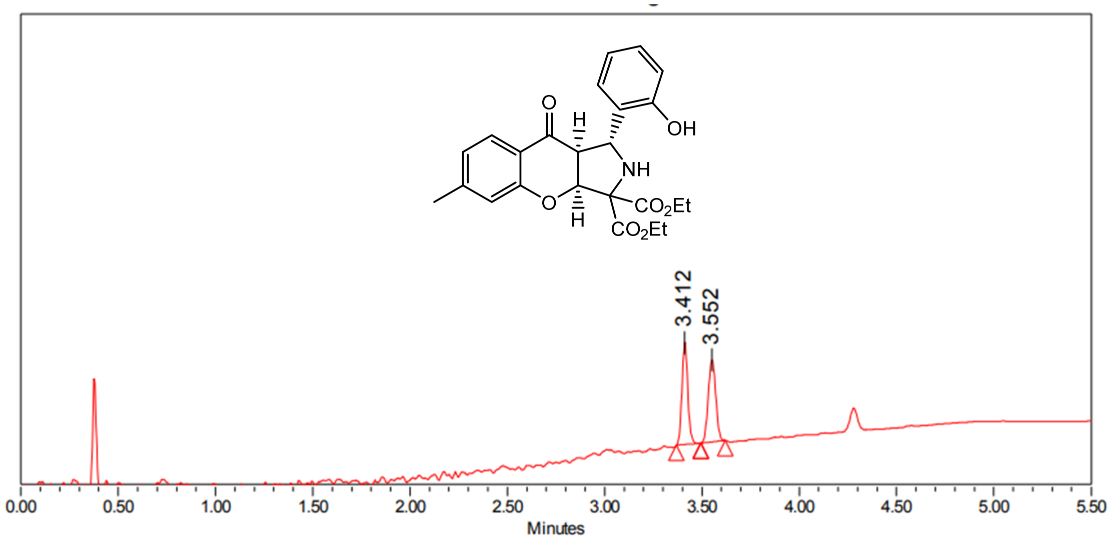

Peak Results

	RT	% Area
1	3.412	49.51
2	3.552	50.49

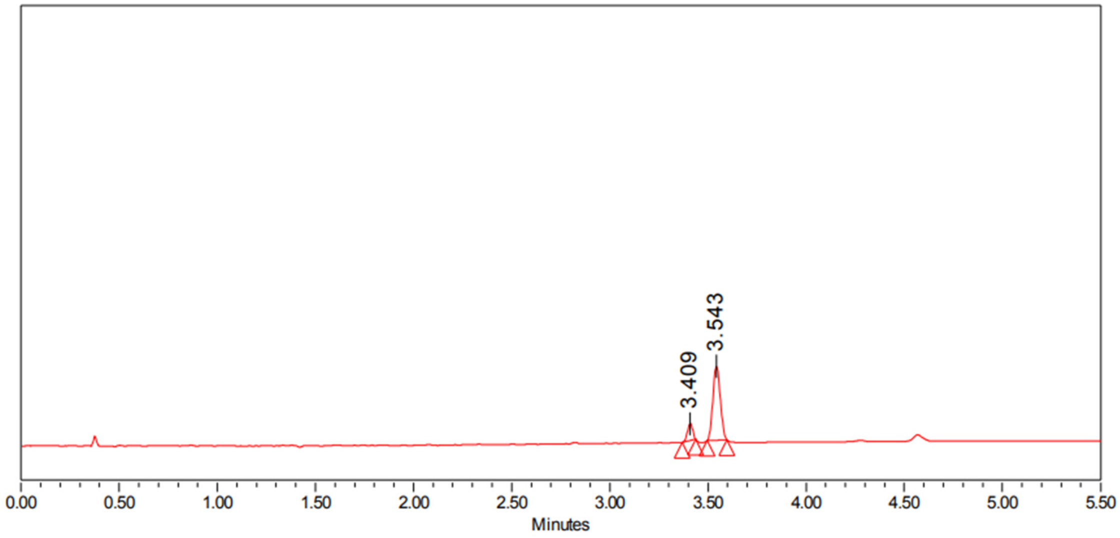

Peak Results

	RT	% Area
1	3.409	13.86
2	3.543	86.14

(1*R*,3a*S*,9a*S*)-Diethyl 7-fluoro-1-(2-hydroxyphenyl)-9-oxo-1,2,9,9a-tetrahydrochromeno[2,3-*c*]pyrrole-3,3(3a*H*)-dicarboxylate 1d

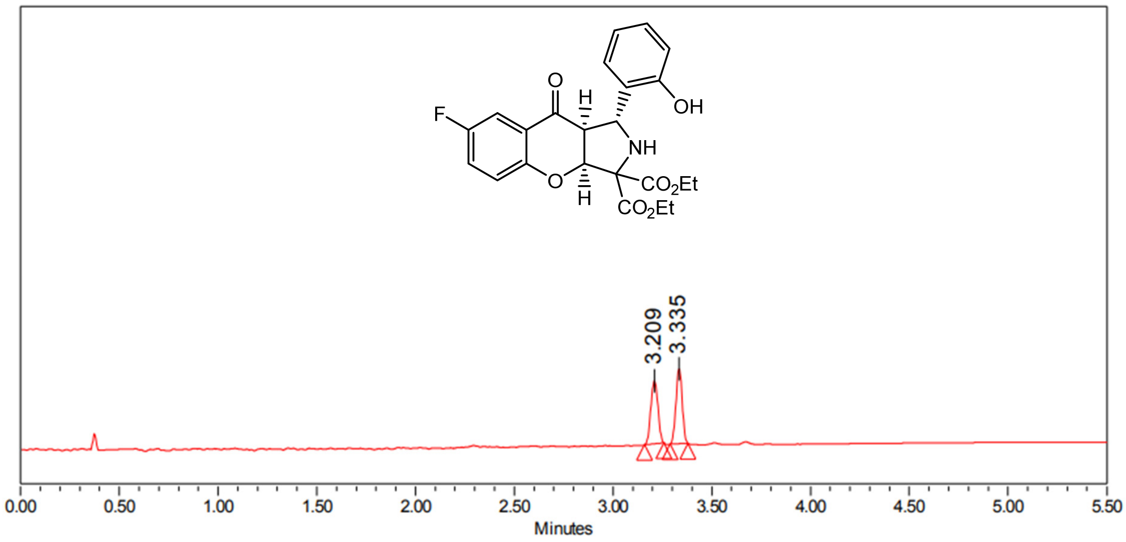

Peak Results

	RT	% Area
1	3.209	49.08
2	3.335	50.92

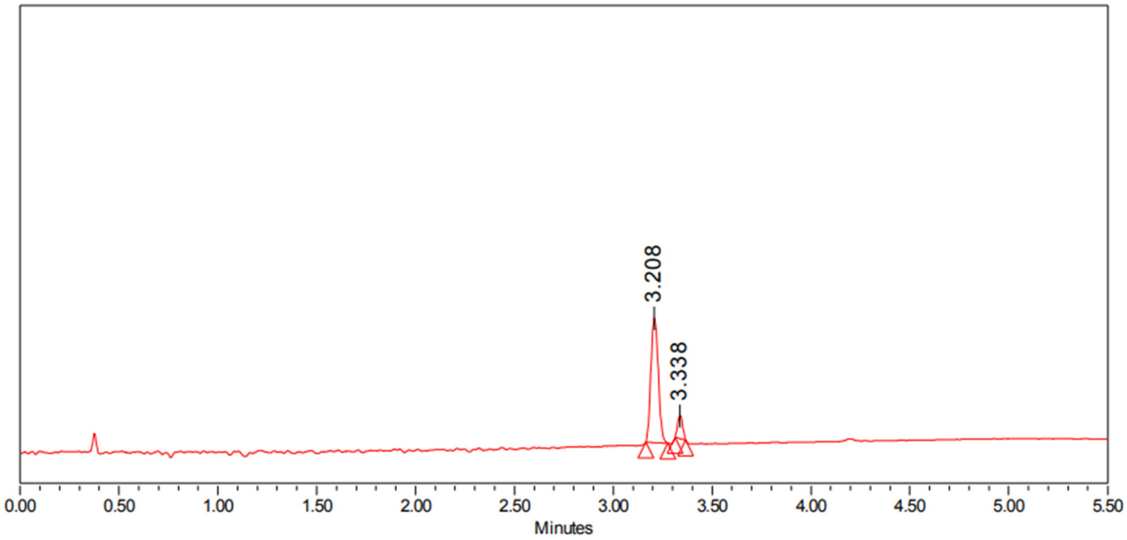

Peak Results

	RT	% Area
1	3.208	88.87
2	3.338	11.13

(1*R*,3a*S*,9a*S*)-Diethyl 7-bromo-1-(2-hydroxyphenyl)-9-oxo-1,2,9,9a-tetrahydrochromeno[2,3-*c*]pyrrole-3,3(3a*H*)-dicarboxylate 1e

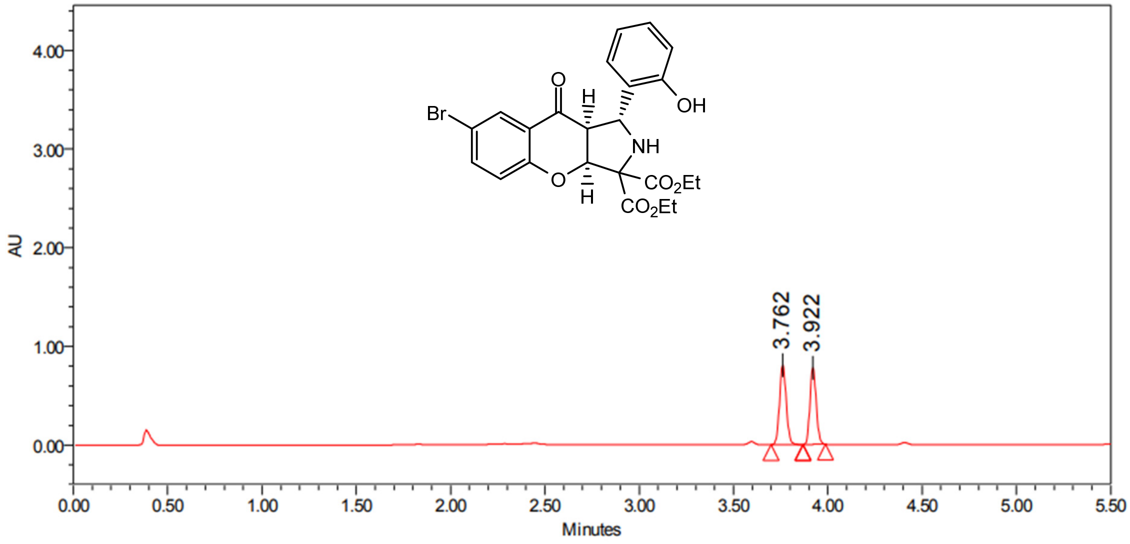

Peak Results

	RT	% Area
1	3.762	52.82
2	3.922	47.18

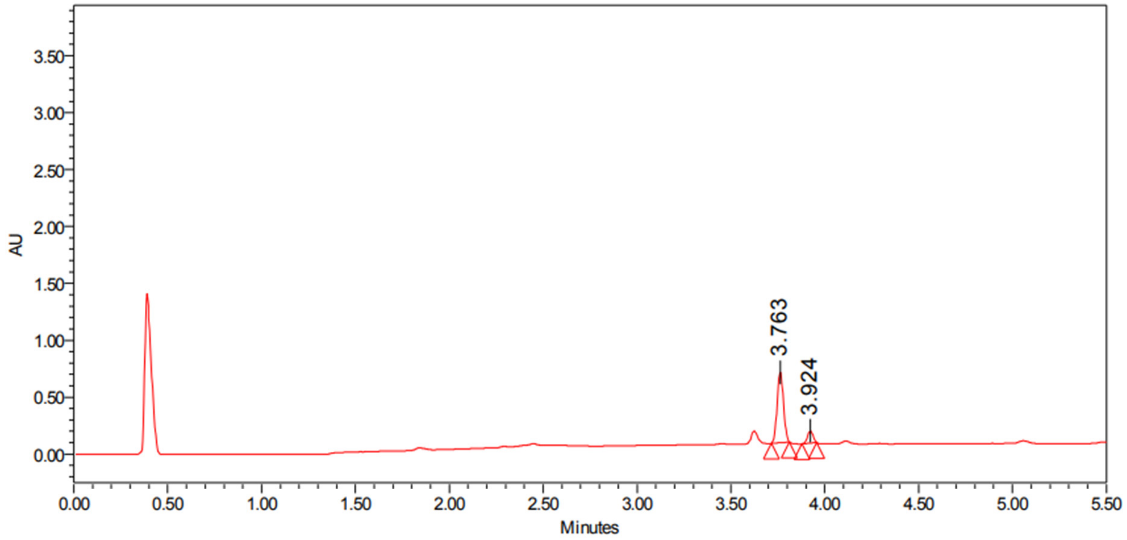

Peak Results

	RT	% Area
1	3.763	86.83
2	3.924	13.17

(1*R*,3a*S*,9a*S*)-Diethyl 7-chloro-1-(2-hydroxyphenyl)-9-oxo-1,2,9,9a-tetrahydrochromeno[2,3-*c*]pyrrole-3,3(3a*H*)-dicarboxylate 1f

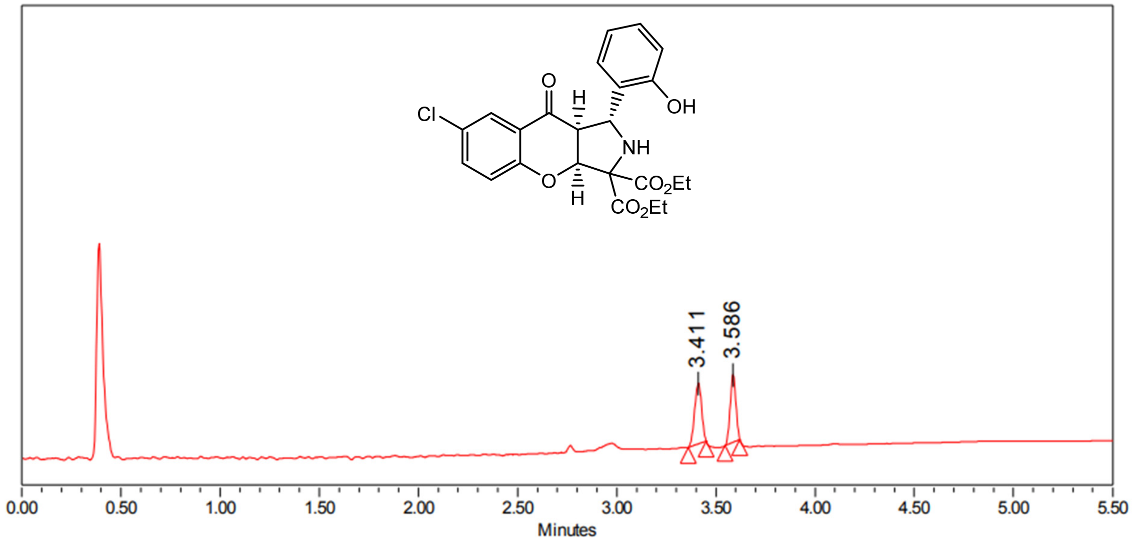

Peak Results

	RT	% Area
1	3.411	52.32
2	3.586	47.68

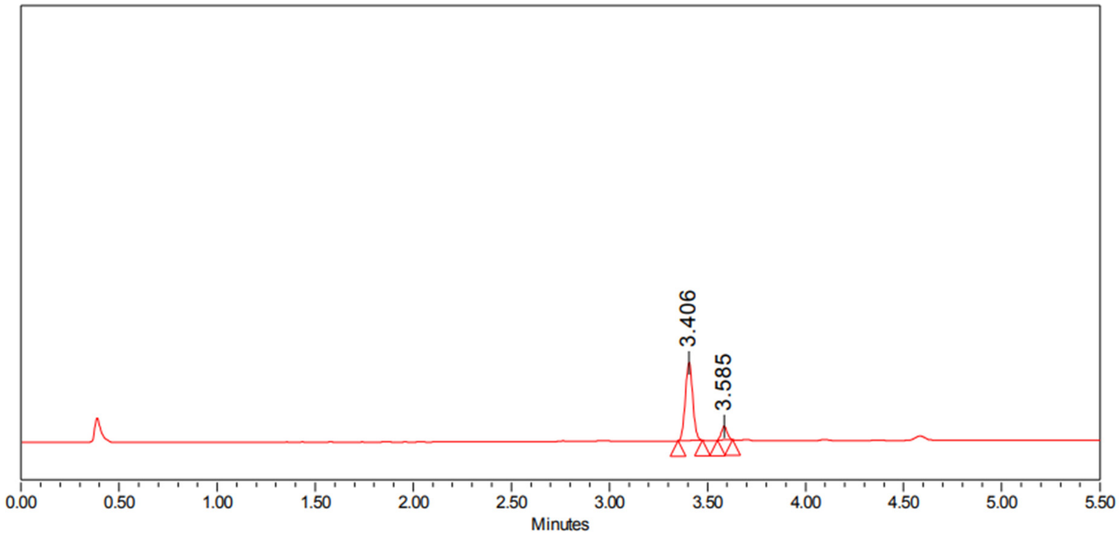

Peak Results

	RT	% Area
1	3.406	87.86
2	3.585	12.14

(1*R*,3a*S*,9a*S*)-Diethyl 7-chloro-1-(2-hydroxyphenyl)-6-methyl-9-oxo-1,2,9,9a-tetrahydrochromeno[2,3-*c*]pyrrole-3,3(3a*H*)-dicarboxylate 1g

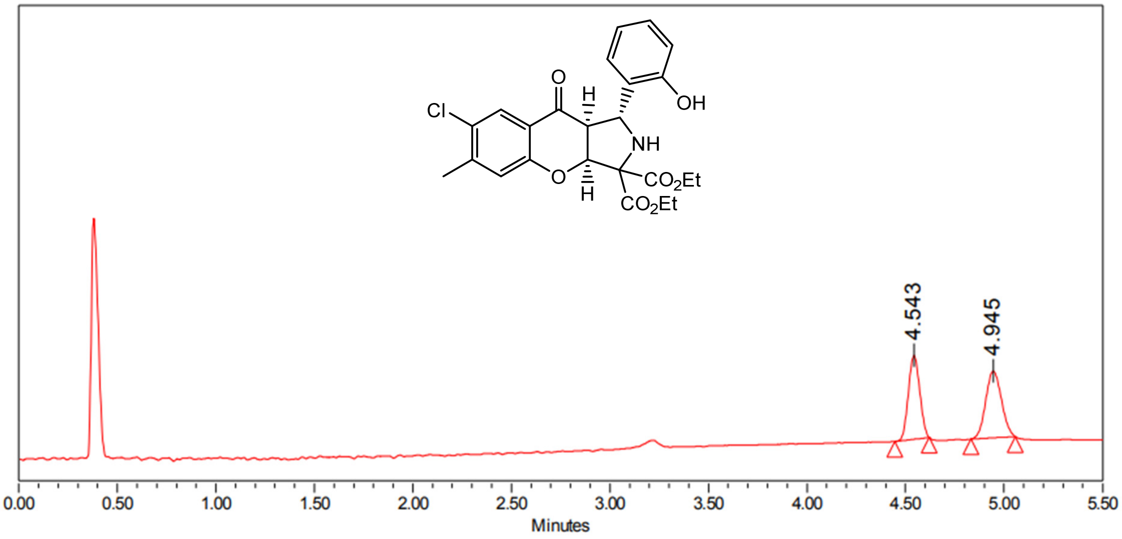

Peak Results

	RT	% Area
1	4.543	47.51
2	4.945	52.49

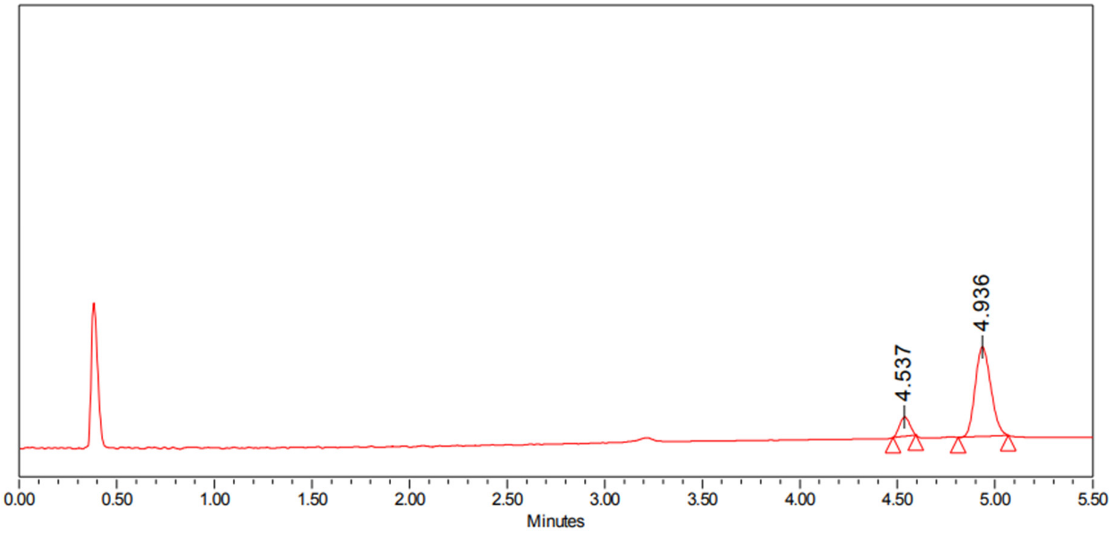

Peak Results

	RT	% Area
1	4.537	12.27
2	4.936	87.73

(7a*S*,10*R*,10a*S*)-Diethyl 10-(2-hydroxyphenyl)-11-oxo-9,10,10a,11-tetrahydrobenzo[5,6]chromeno[2,3-*c*]pyrrole-8,8(7a*H*)-dicarboxylate 1h

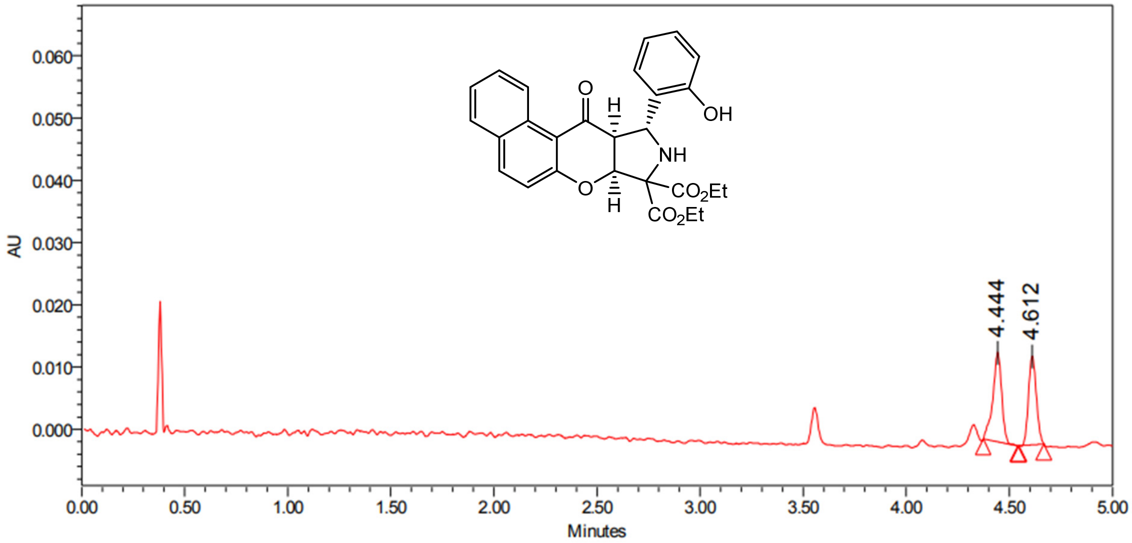

Peak Results

	RT	% Area
1	4.444	53.31
2	4.612	46.69

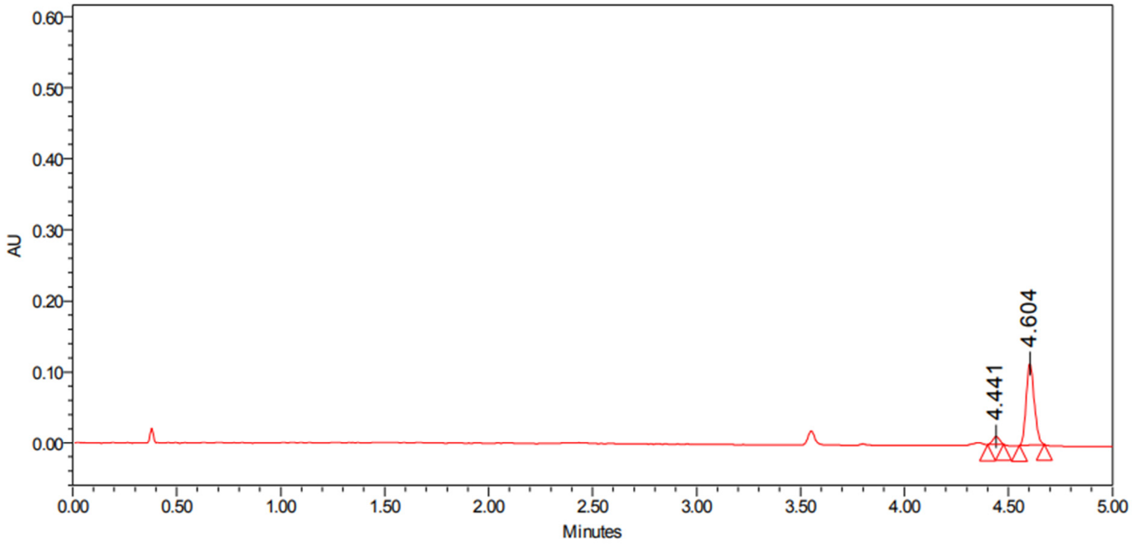

Peak Results

	RT	% Area
1	4.441	7.78
2	4.604	92.22

(1*R*,3a*S*,9a*S*)-Diethyl 1-(2-hydroxy-5-methylphenyl)-9-oxo-1,2,9,9a-tetrahydrochromeno[2,3-*c*]pyrrole-3,3(3a*H*)-dicarboxylate 1i

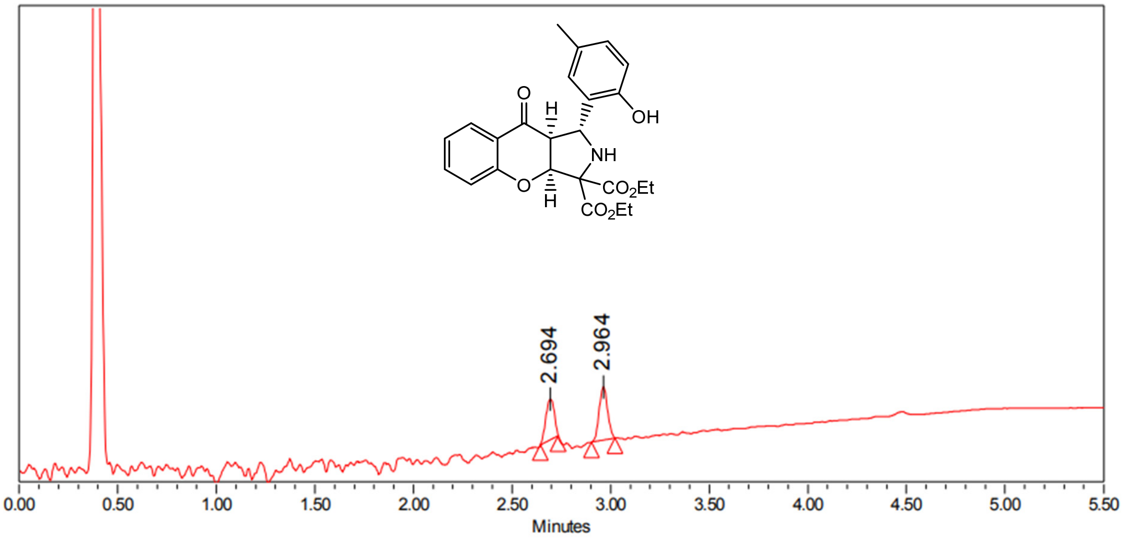

Peak Results

	RT	% Area
1	2.694	43.33
2	2.964	56.67

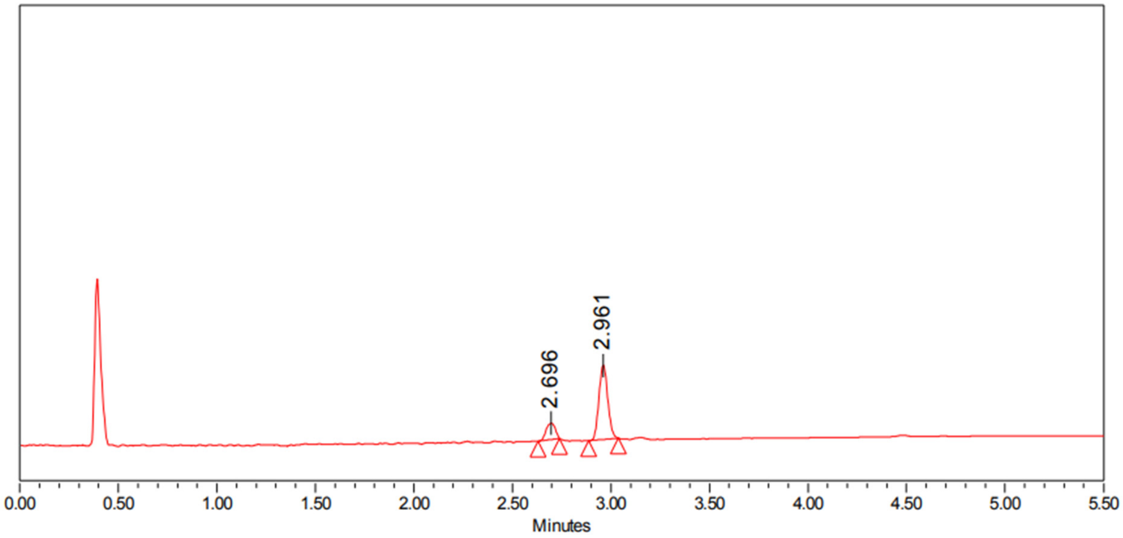

Peak Results

	RT	% Area
1	2.696	18.28
2	2.961	81.72

(1*R*,3a*S*,9a*S*)-Diethyl 1-(5-chloro-2-hydroxyphenyl)-9-oxo-1,2,9,9a-tetrahydrochromeno[2,3-*c*]pyrrole-3,3(3a*H*)-dicarboxylate 1j

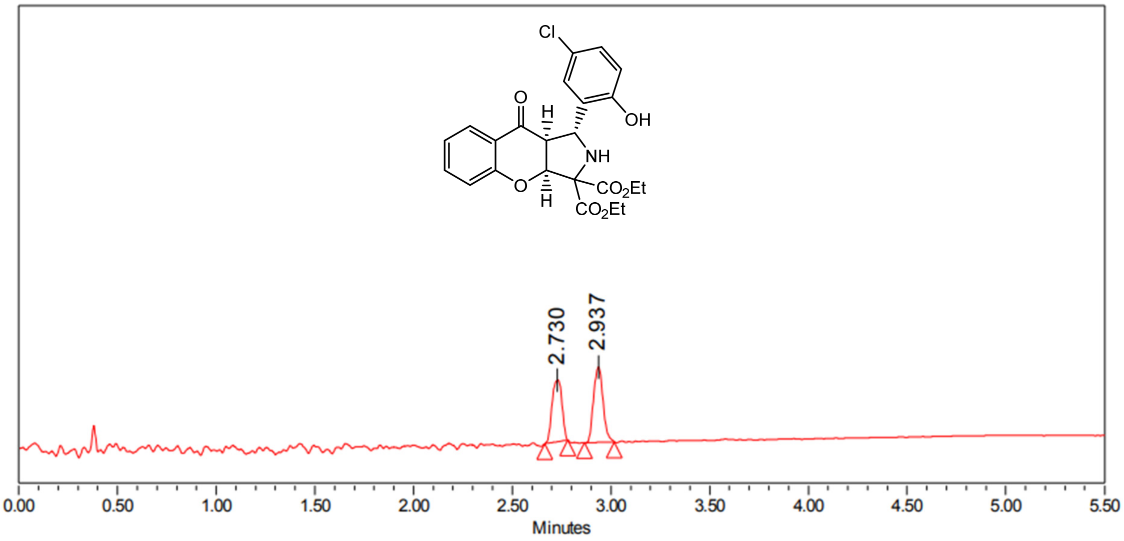

Peak Results

	RT	% Area
1	2.730	46.10
2	2.937	53.90

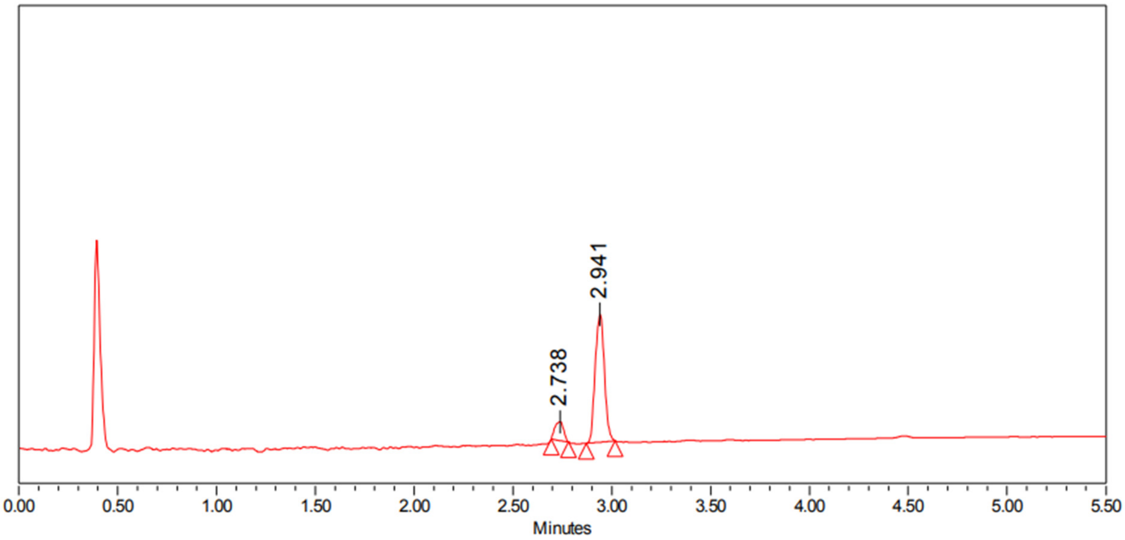

Peak Results

	RT	% Area
1	2.738	12.28
2	2.941	87.72

(1*R*,3a*S*,9a*S*)-Diethyl 1-(5-(*tert*-butyl)-2-hydroxyphenyl)-9-oxo-1,2,9,9a-tetrahydrochromeno[2,3-*c*]pyrrole-3,3(3a*H*)-dicarboxylate 1k

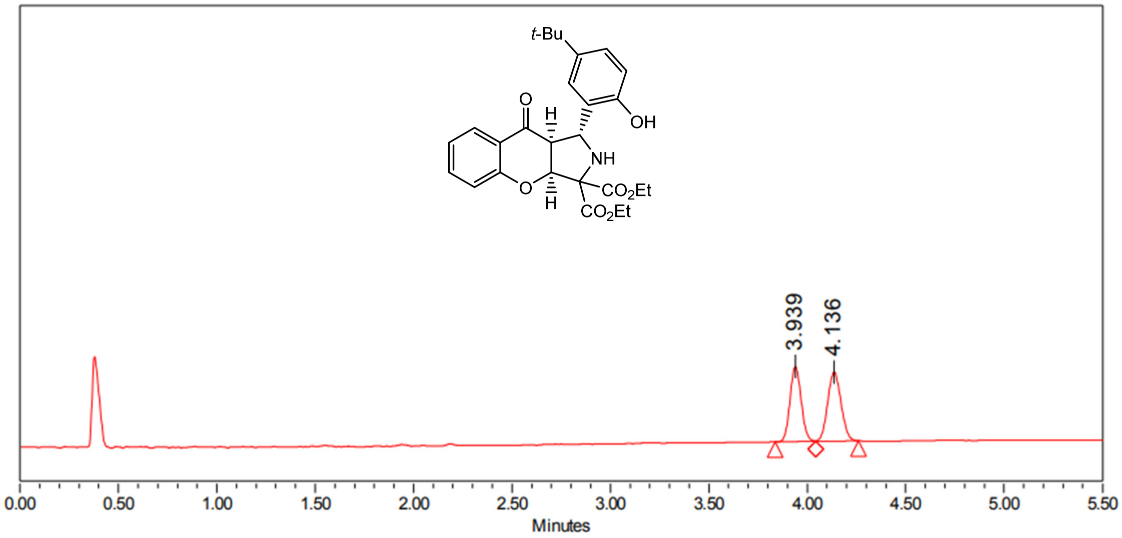

Peak Results

	RT	% Area
1	3.939	47.55
2	4.136	52.45

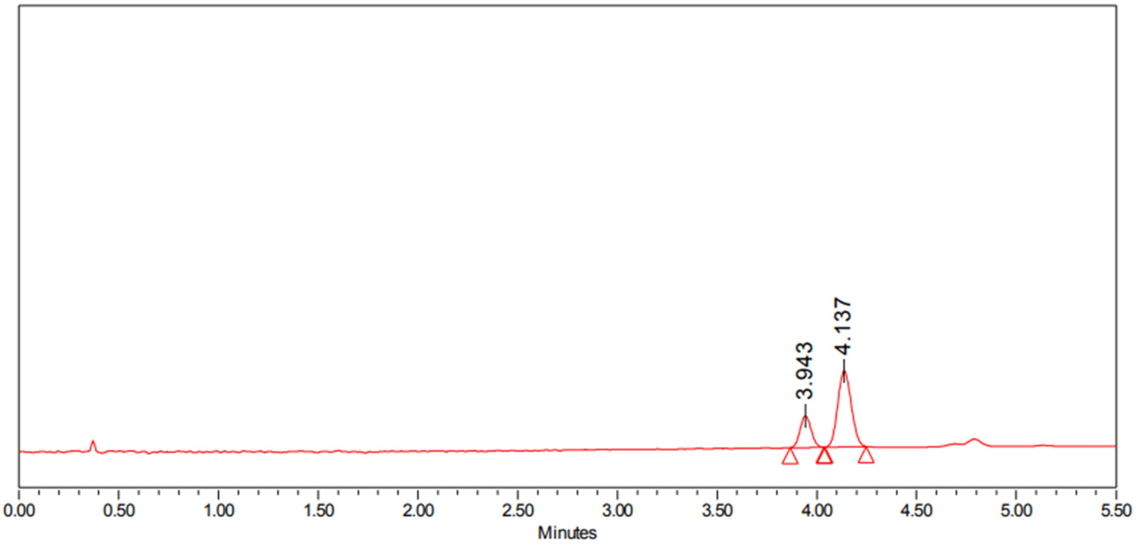

Peak Results

	RT	% Area
1	3.943	25.49
2	4.137	74.51

(1*R*,3a*S*,9a*S*)-Diethyl 1-(2-hydroxy-5-nitrophenyl)-9-oxo-1,2,9,9a-tetrahydrochromeno[2,3-*c*]pyrrole-3,3(3a*H*)-dicarboxylate 1l

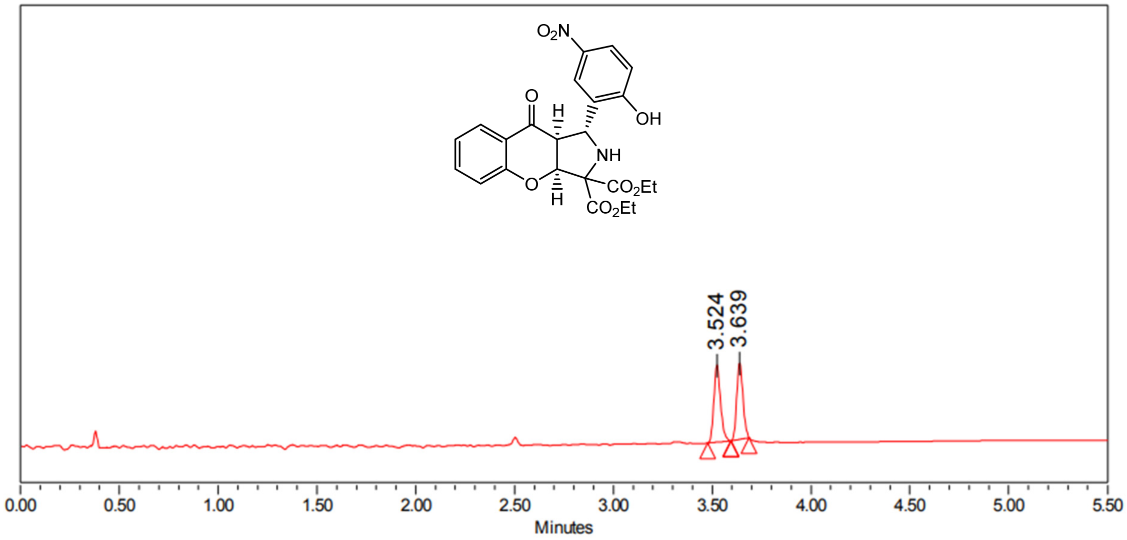

Peak Results

	RT	% Area
1	3.524	52.06
2	3.639	47.94

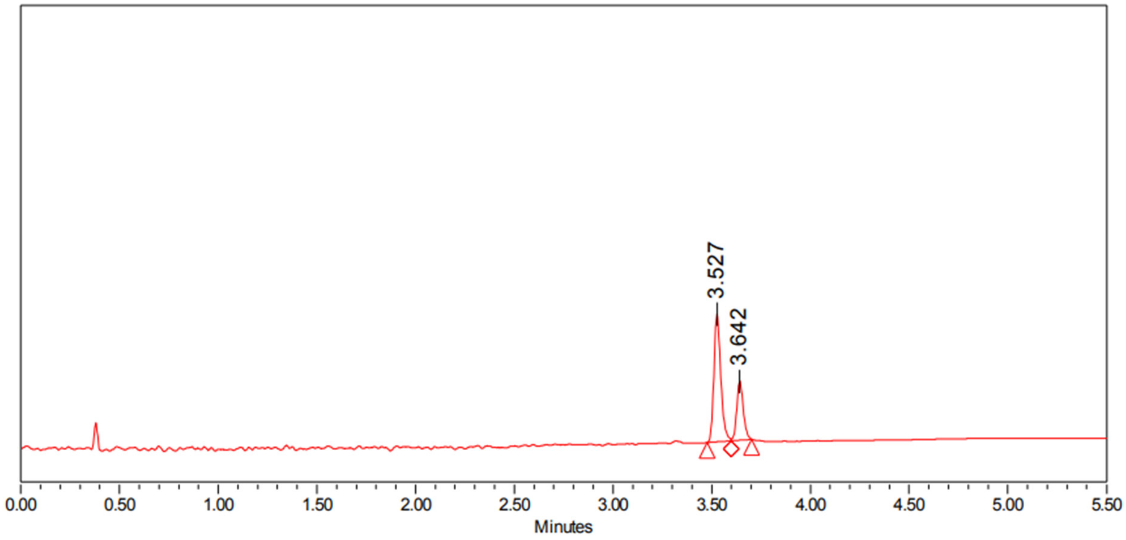

Peak Results

	RT	% Area
1	3.527	69.58
2	3.642	30.42

(1*R*,3*R*,3a*S*,9a*S*)-1-(2-Hydroxyphenyl)-3a,4',5',9a-tetrahydro-1*H*,2'*H*-spiro[chromeno[2,3-*c*]pyrrole-3,3'-furan]-2',9(2*H*)-dione 1m

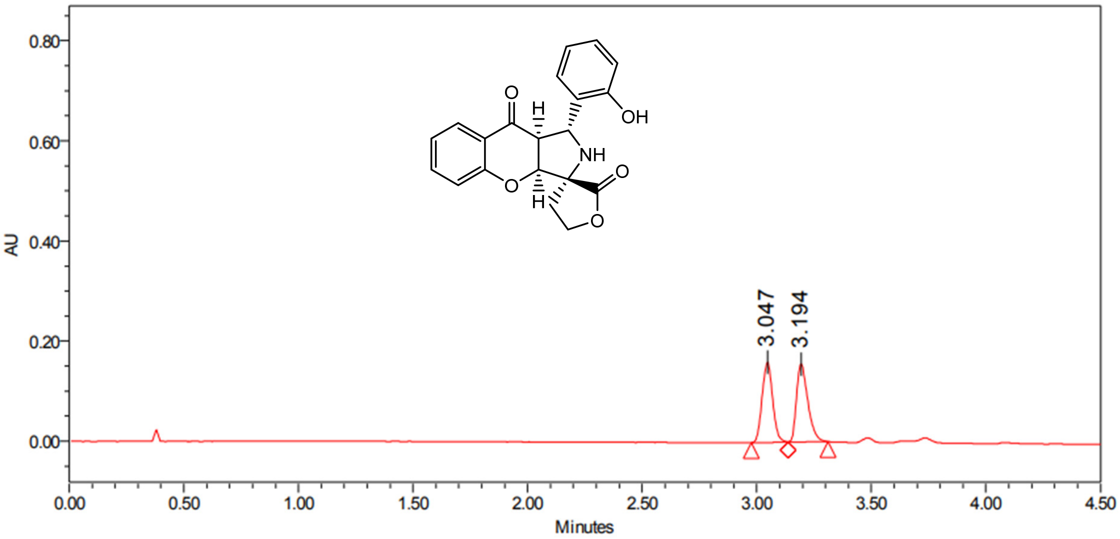

Peak Results

	RT	% Area
1	3.047	48.93
2	3.194	51.07

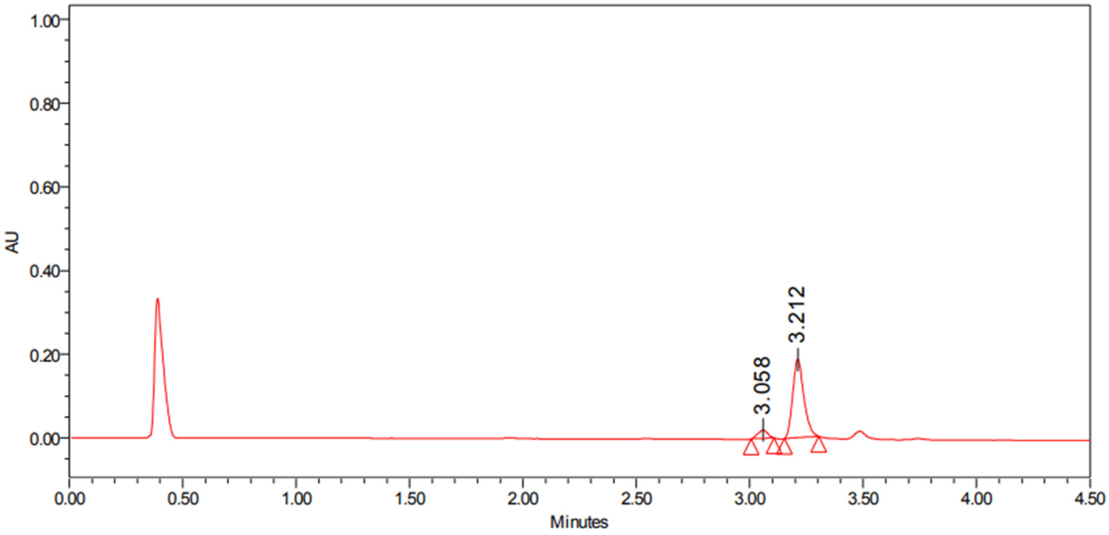

Peak Results

	RT	% Area
1	3.058	8.47
2	3.212	91.53

(1*R*,3*S*,3a*S*,9a*S*)-1-(2-Hydroxyphenyl)-3a,4',5',9a-tetrahydro-1*H*,2'*H*-spiro[chromeno[2,3-*c*]pyrrole-3,3'-furan]-2',9(2*H*)-dione 1m'

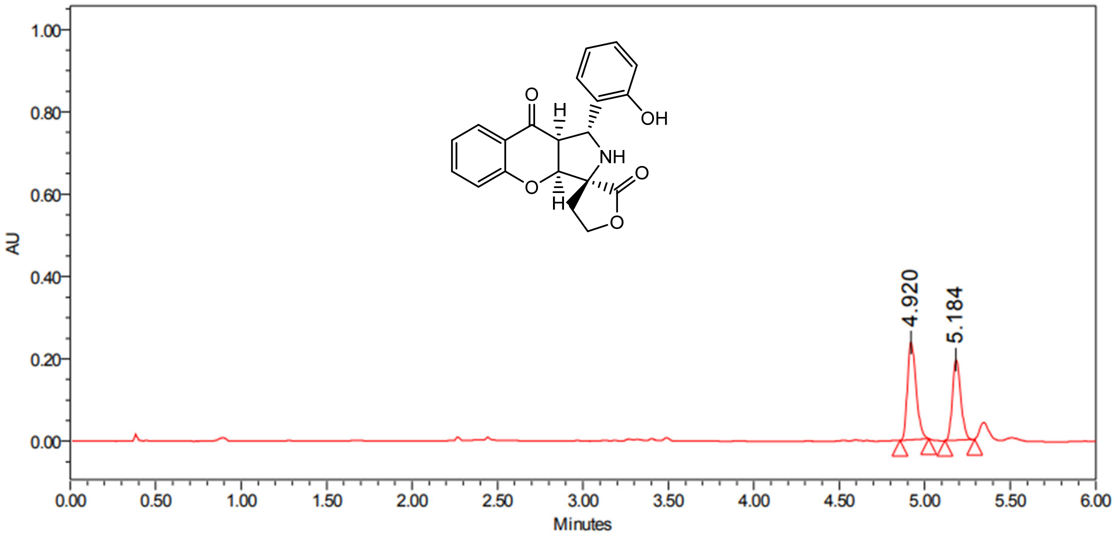

Peak Results

	RT	% Area
1	4.920	55.72
2	5.184	44.28

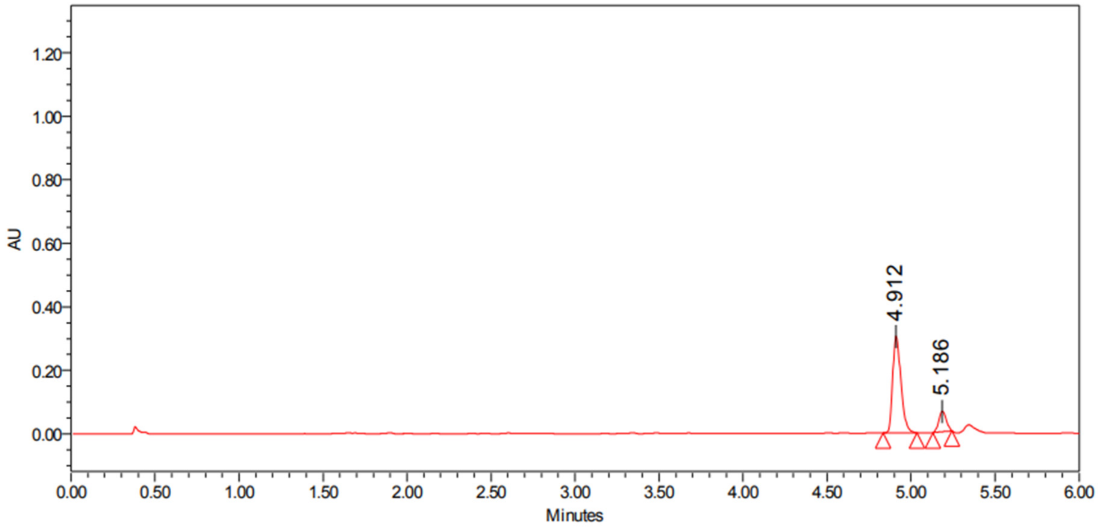

Peak Results

	RT	% Area
1	4.912	83.96
2	5.186	16.04

(1*R*,3*R*,3a*S*,9a*S*)-1-(2-Hydroxyphenyl)-7-methyl-3a,4',5',9a-tetrahydro-1*H*,2'*H*-spiro[chromeno[2,3-*c*]pyrrole-3,3'-furan]-2',9(2*H*)-dione 1n

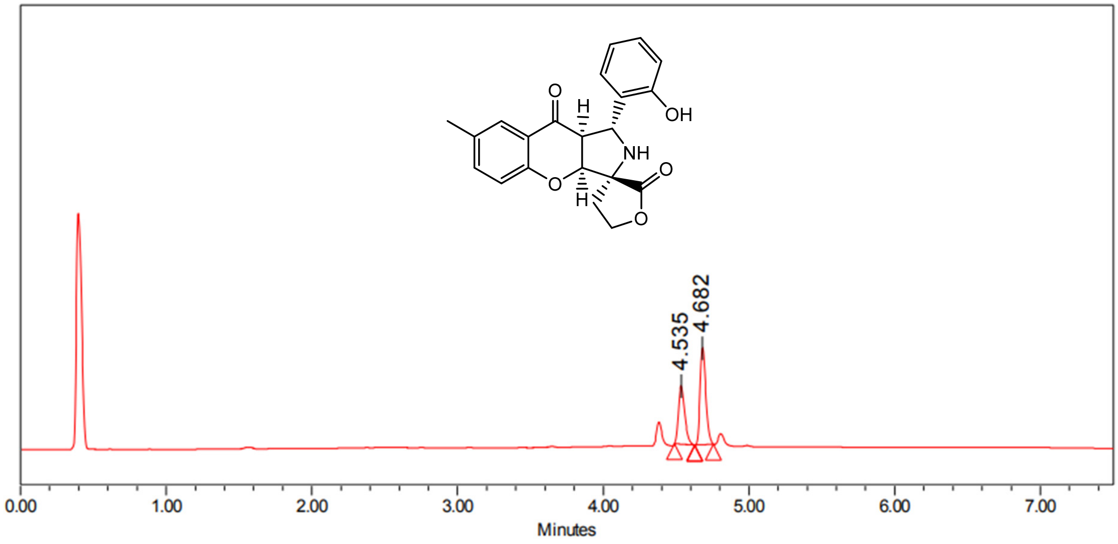

Peak Results

	RT	% Area
1	4.535	39.71
2	4.682	60.29

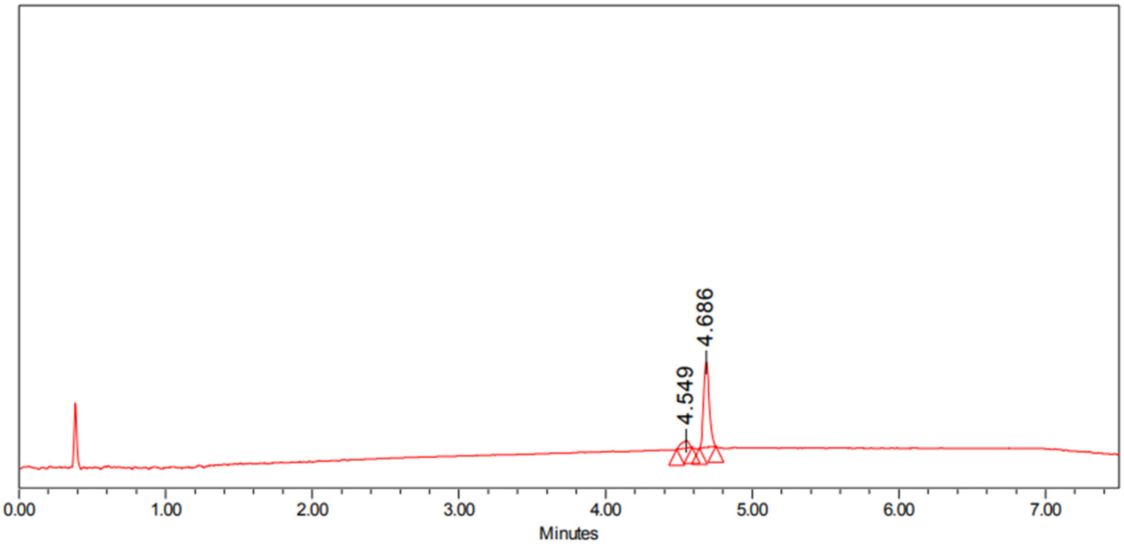

Peak Results

	RT	% Area
1	4.549	11.05
2	4.686	88.95

(1*R*,3*S*,3a*S*,9a*S*)-1-(2-Hydroxyphenyl)-7-methyl-3a,4',5',9a-tetrahydro-1*H*,2'*H*-spiro[chromeno[2,3-*c*]pyrrole-3,3'-furan]-2',9(2*H*)-dione 1n'

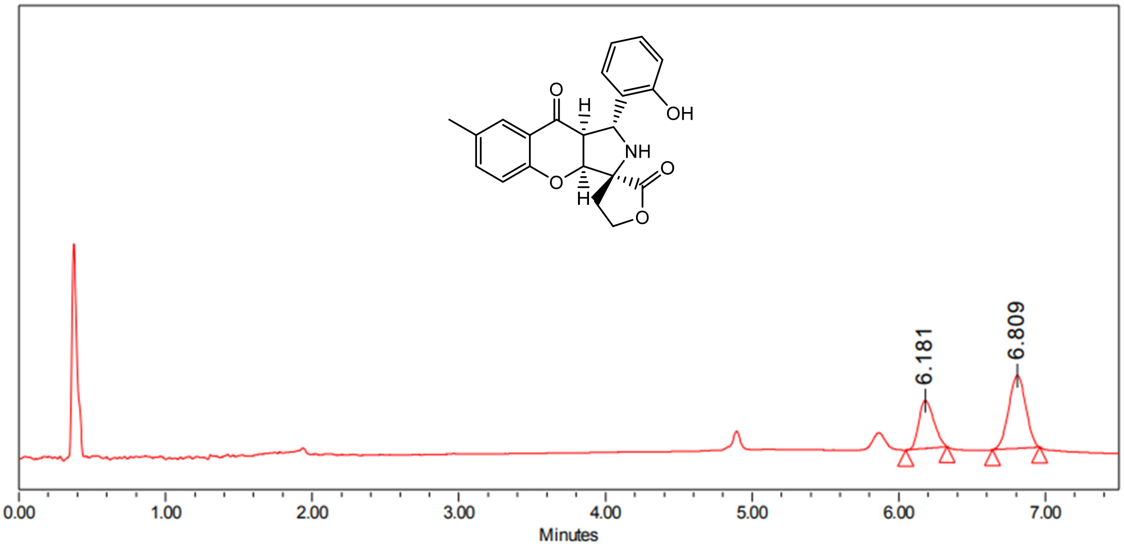

Peak Results

	RT	% Area
1	6.181	36.64
2	6.809	63.36

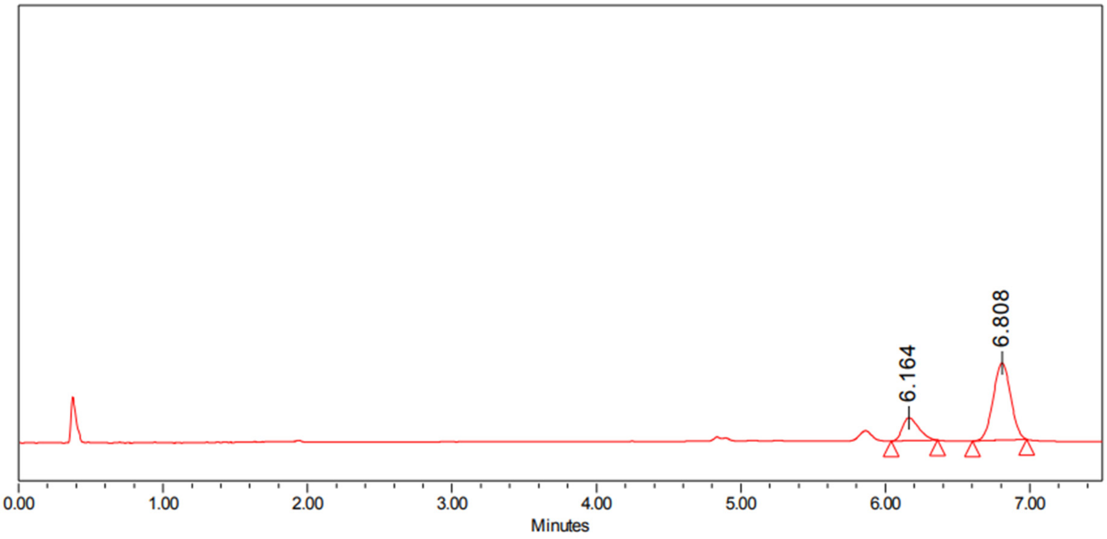

Peak Results

	RT	% Area
1	6.164	21.85
2	6.808	78.15

(1*R*,3*R*,3a*S*,9a*S*)-1-(2-Hydroxyphenyl)-6-methyl-3a,4',5',9a-tetrahydro-1*H*,2'*H*-spiro[chromeno[2,3-*c*]pyrrole-3,3'-furan]-2',9(2*H*)-dione 1o

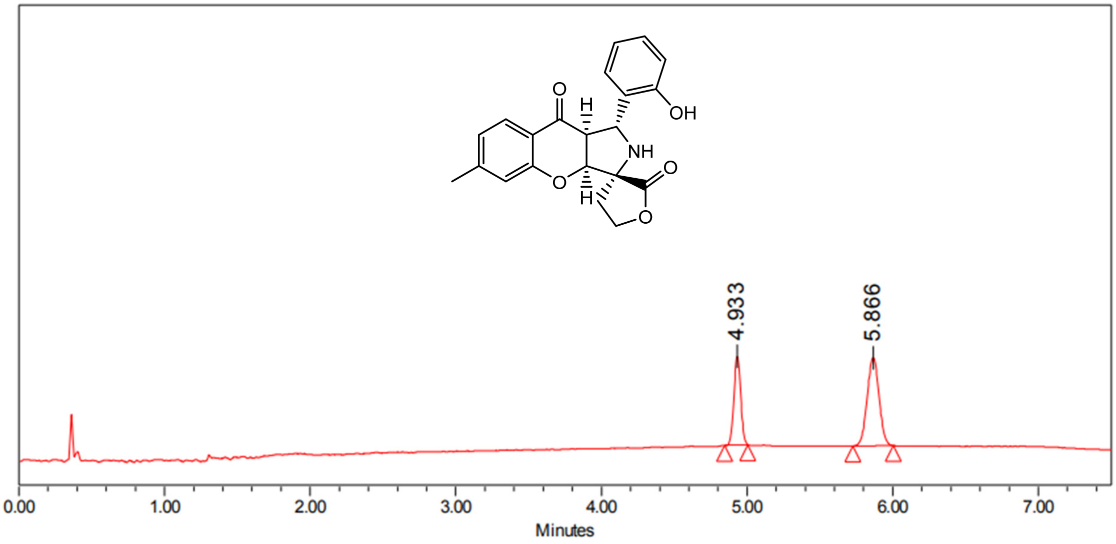

Peak Results

	RT	% Area
1	4.933	36.27
2	5.866	63.73

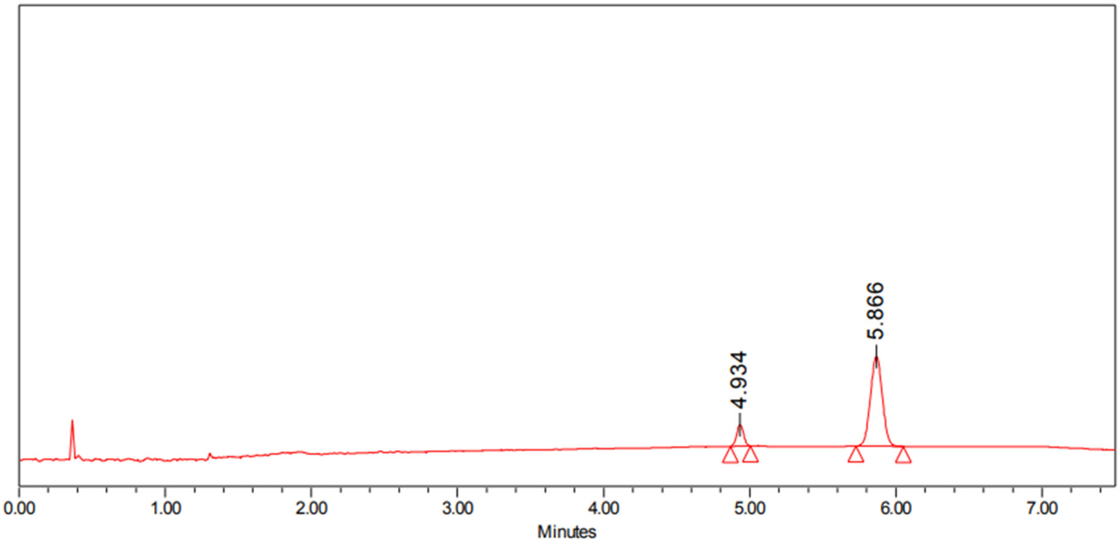

Peak Results

	RT	% Area
1	4.934	12.43
2	5.866	87.57

(1*R*,3*S*,3a*S*,9a*S*)-1-(2-Hydroxyphenyl)-6-methyl-3a,4',5',9a-tetrahydro-1*H*,2'*H*-spiro[chromeno[2,3-*c*]pyrrole-3,3'-furan]-2',9(2*H*)-dione 1o'

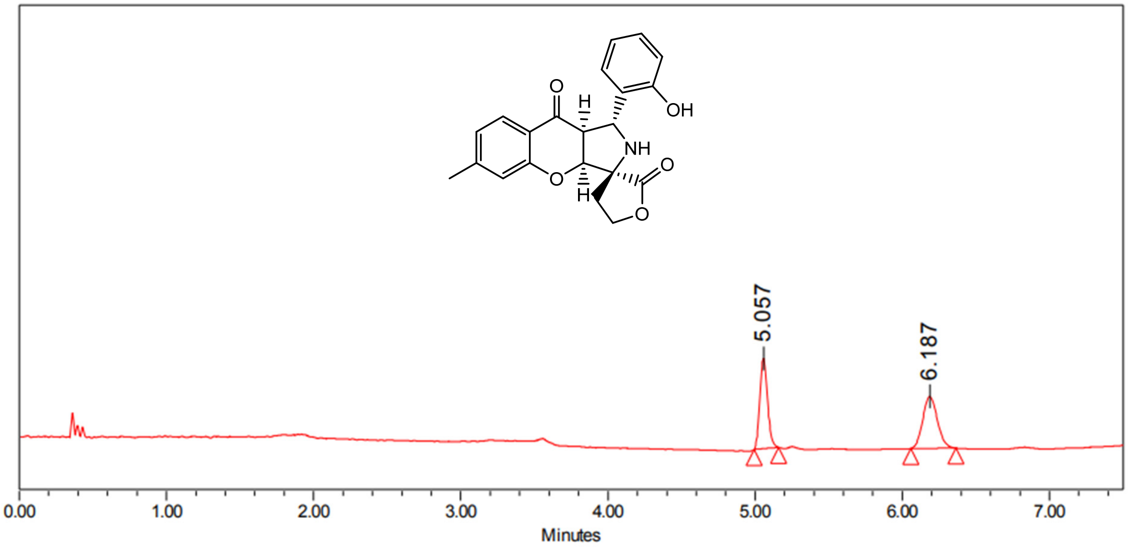

Peak Results

	RT	% Area
1	5.057	47.80
2	6.187	52.20

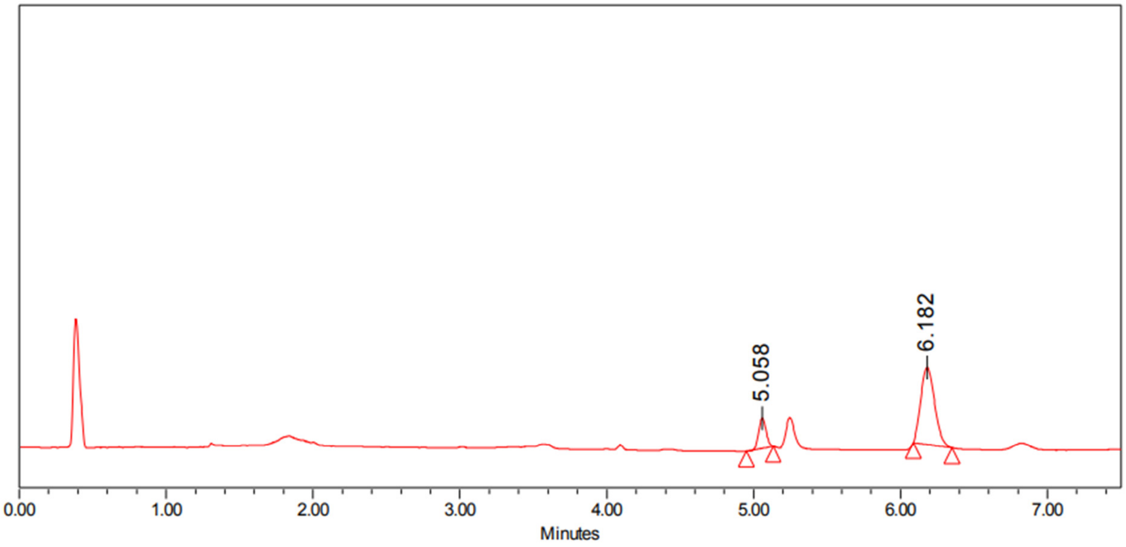

Peak Results

	RT	% Area
1	5.058	17.48
2	6.182	82.52

(1*R*,3*R*,3a*S*,9a*S*)-7-Chloro-1-(2-hydroxyphenyl)-3a,4',5',9a-tetrahydro-1*H*,2'*H*-spiro[chromeno[2,3-*c*]pyrrole-3,3'-furan]-2',9(2*H*)-dione 1p

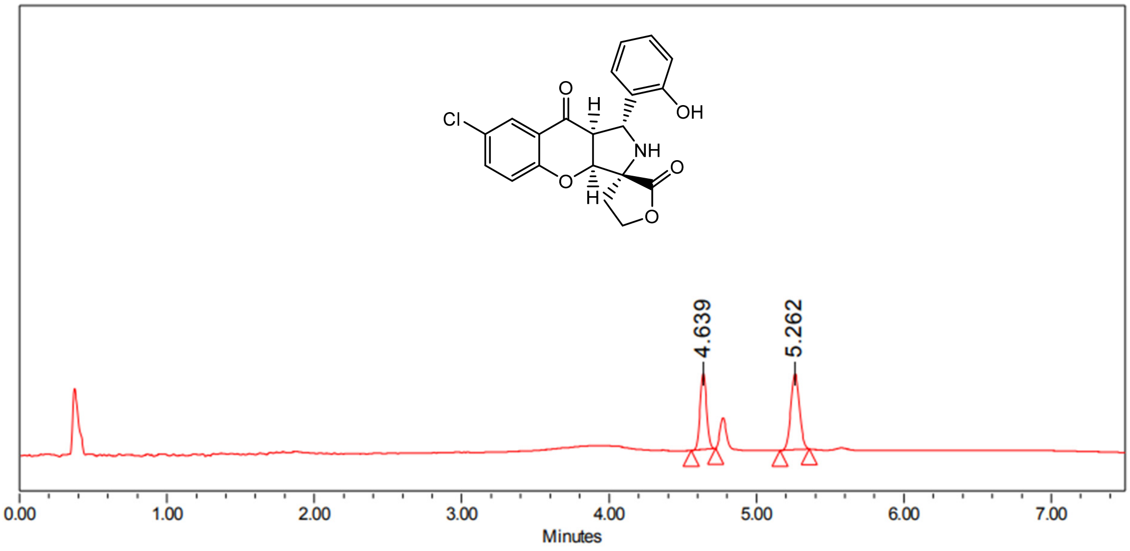

Peak Results

	RT	% Area
1	4.639	42.70
2	5.262	57.30

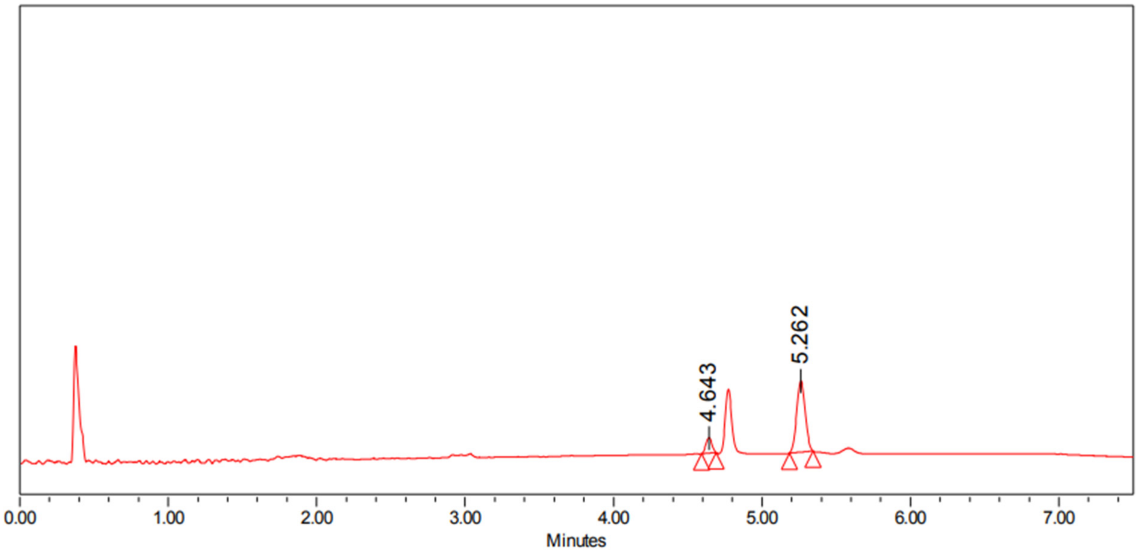

Peak Results

	RT	% Area
1	4.643	12.91
2	5.262	87.09

(1*R*,3*S*,3a*S*,9a*S*)-7-Chloro-1-(2-hydroxyphenyl)-3a,4',5',9a-tetrahydro-1*H*,2'*H*-spiro[chromeno[2,3-*c*]pyrrole-3,3'-furan]-2',9(2*H*)-dione 1p’

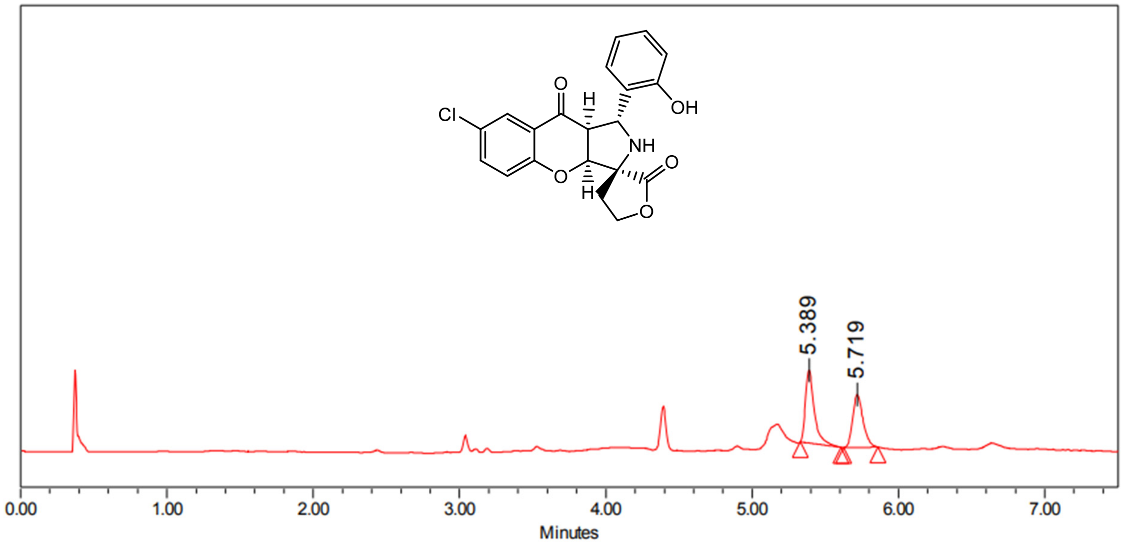

Peak Results

	RT	% Area
1	5.389	54.43
2	5.719	45.57

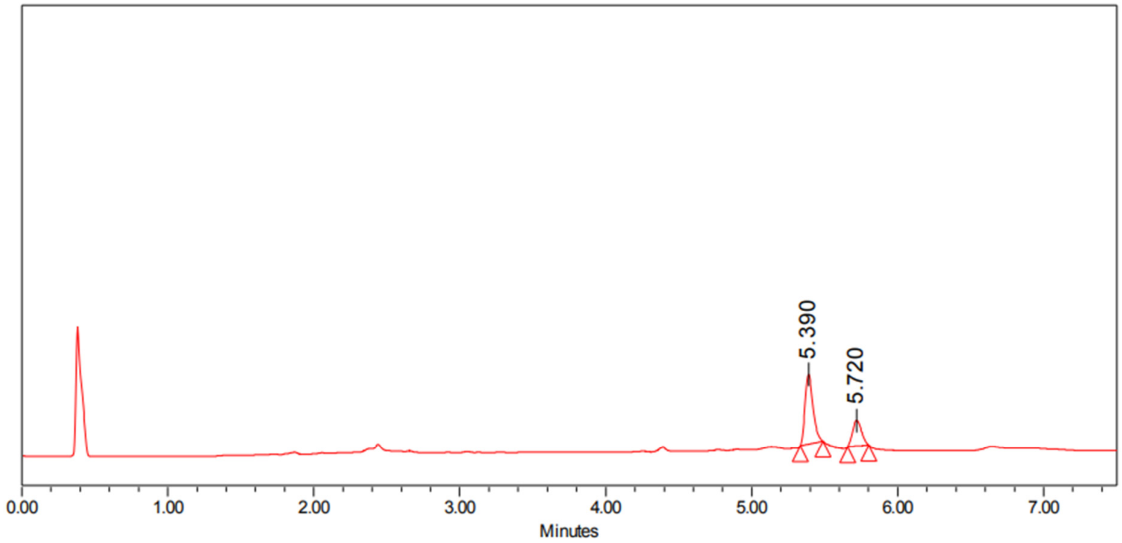

Peak Results

	RT	% Area
1	5.390	70.77
2	5.720	29.23

(1*R*,3*R*,3a*S*,9a*S*)-6-Fluoro-1-(2-hydroxyphenyl)-3a,4',5',9a-tetrahydro-1*H*,2'*H*-spiro[chromeno[2,3-*c*]pyrrole-3,3'-furan]-2',9(2*H*)-dione 1r

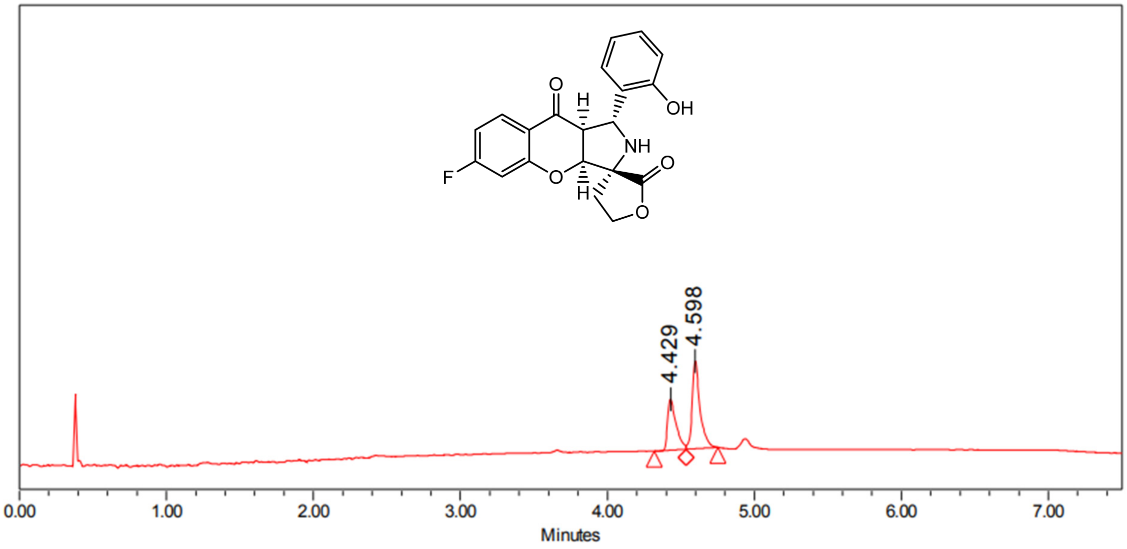

Peak Results

	RT	% Area
1	4.429	38.75
2	4.598	61.25

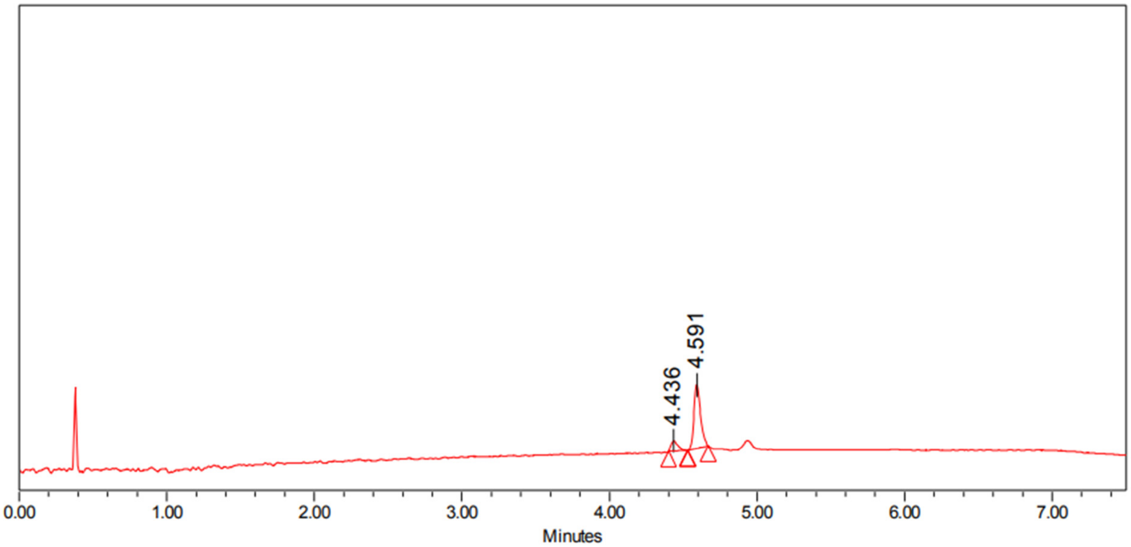

Peak Results

	RT	% Area
1	4.436	14.17
2	4.591	85.83

(1*R*,3*S*,3a*S*,9a*S*)-6-Fluoro-1-(2-hydroxyphenyl)-3a,4',5',9a-tetrahydro-1*H*,2'*H*-spiro[chromeno[2,3-*c*]pyrrole-3,3'-furan]-2',9(2*H*)-dione 1r'

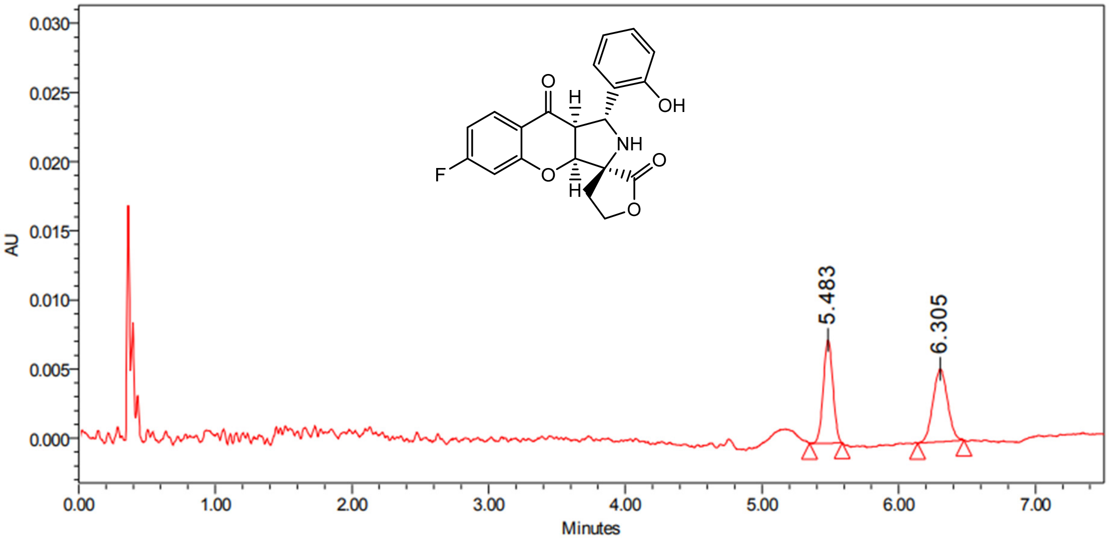

Peak Results

	RT	% Area
1	5.483	48.40
2	6.305	51.60

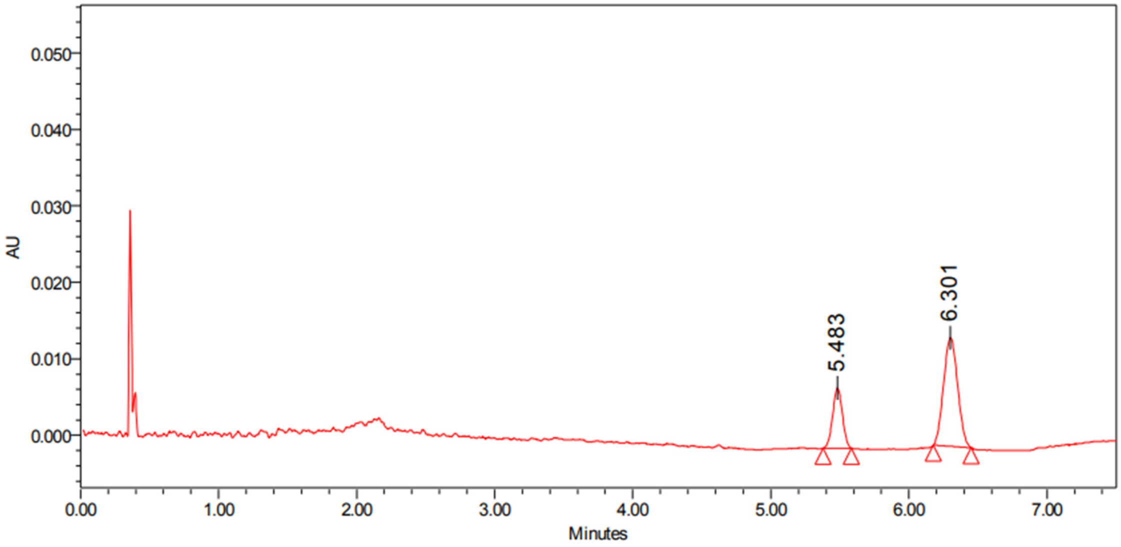

Peak Results

	RT	% Area
1	5.483	27.88
2	6.301	72.12

(1*R*,3*R*,3a*S*,9a*S*)-7-Chloro-1-(2-hydroxyphenyl)-6-methyl-3a,4',5',9a-tetrahydro-1*H*,2'*H*-spiro[chromeno[2,3-*c*]pyrrole-3,3'-furan]-2',9(2*H*)-dione 1s

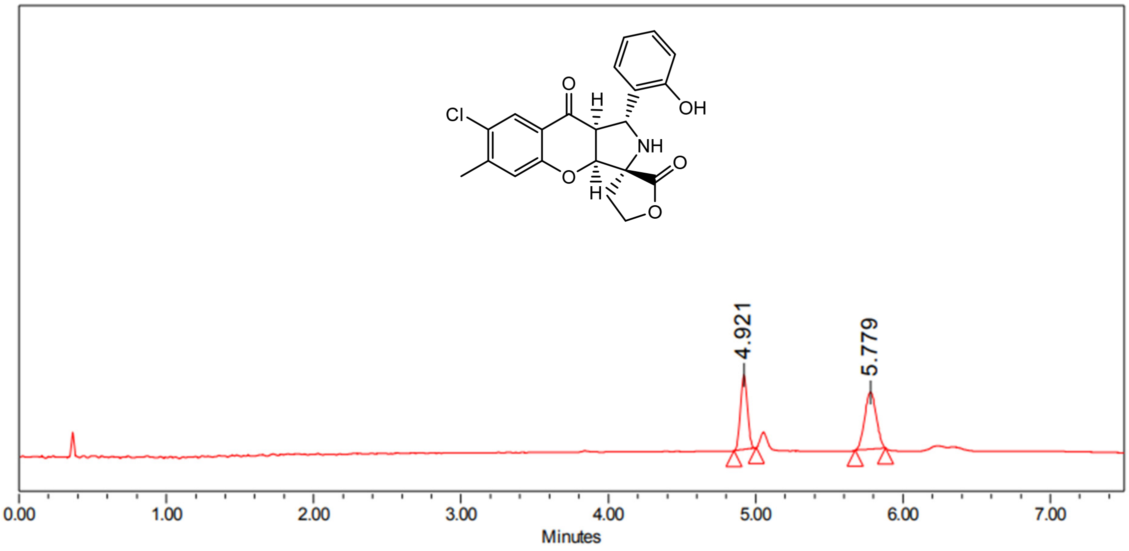

Peak Results

	RT	% Area
1	4.921	44.00
2	5.779	56.00

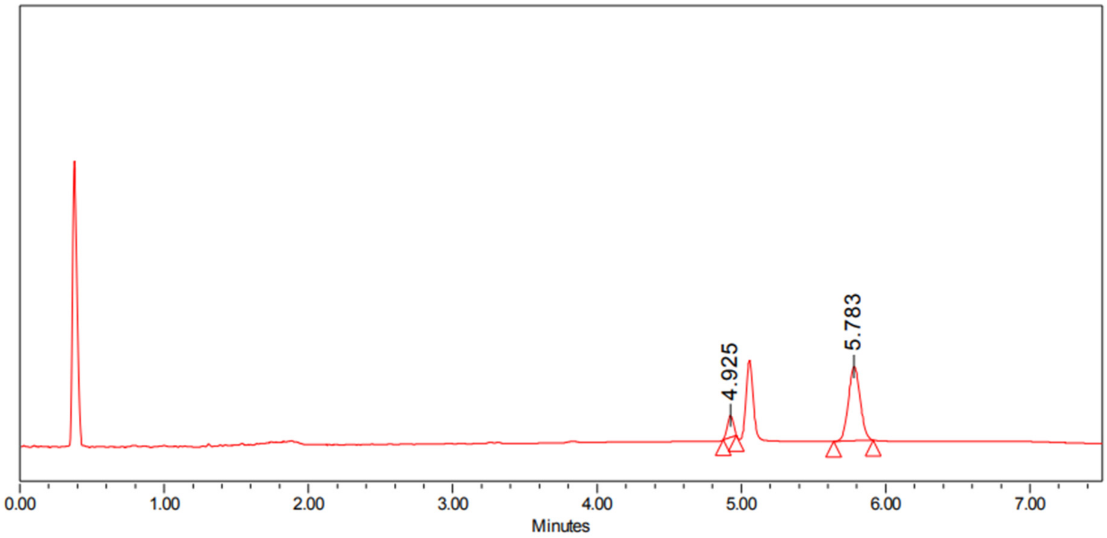

Peak Results

	RT	% Area
1	4.925	13.09
2	5.783	86.91

(1*R*,3*S*,3a*S*,9a*S*)-7-Chloro-1-(2-hydroxyphenyl)-6-methyl-3a,4',5',9a-tetrahydro-1*H*,2'*H*-spiro[chromeno[2,3-*c*]pyrrole-3,3'-furan]-2',9(2*H*)-dione 1s'

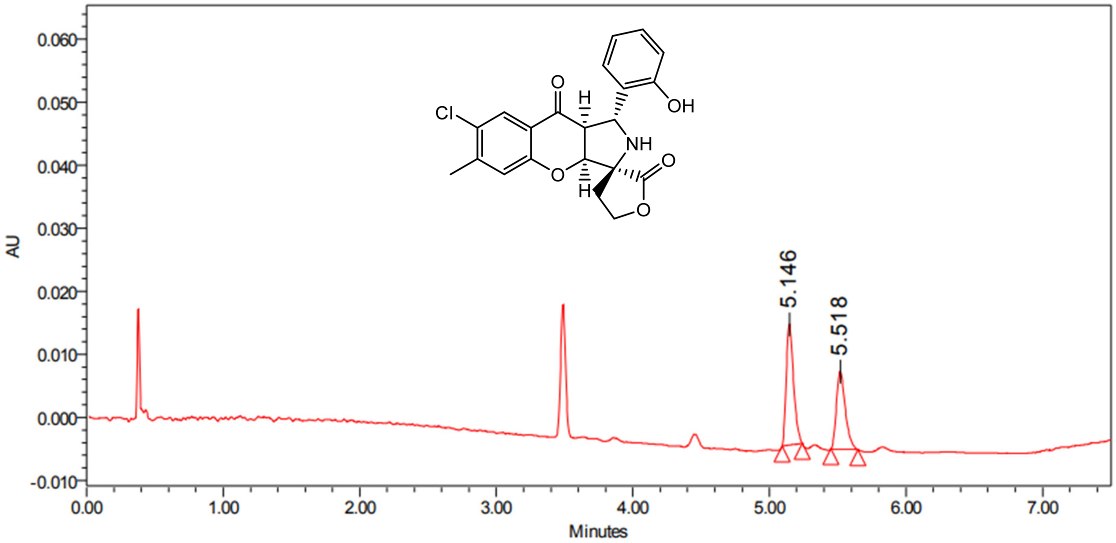

Peak Results

	RT	% Area
1	5.146	55.94
2	5.518	44.06

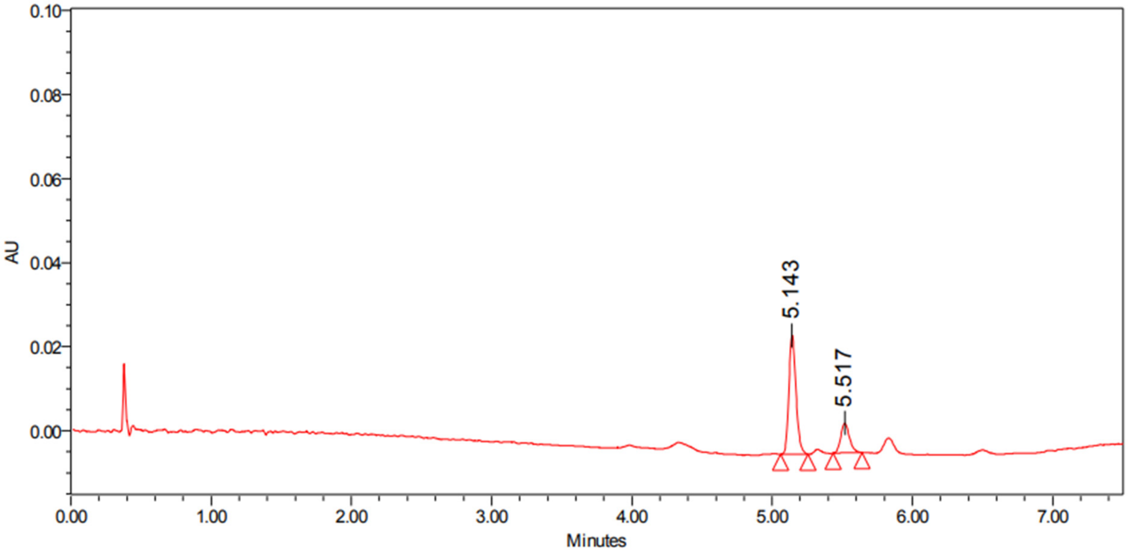

Peak Results

	RT	% Area
1	5.143	76.65
2	5.517	23.35

(1*R*,3*R*,3a*S*,9a*S*)-1-(2-Hydroxy-5-methylphenyl)-3a,4',5',9a-tetrahydro-1*H*,2'*H*-spiro[chromeno[2,3-*c*]pyrrole-3,3'-furan]-2',9(2*H*)-dione 1t

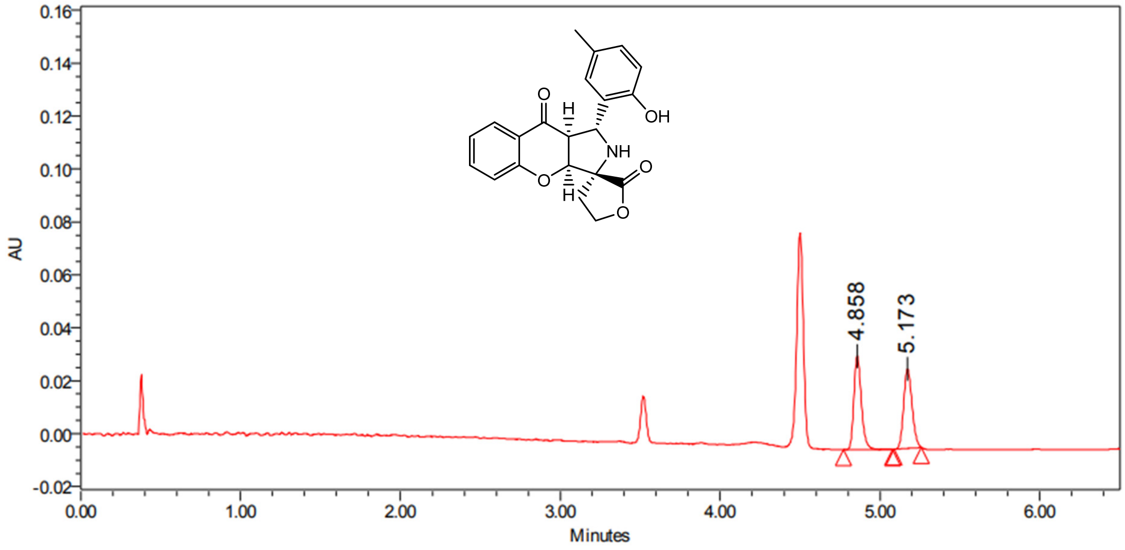

Peak Results

	RT	% Area
1	4.858	51.16
2	5.173	48.84

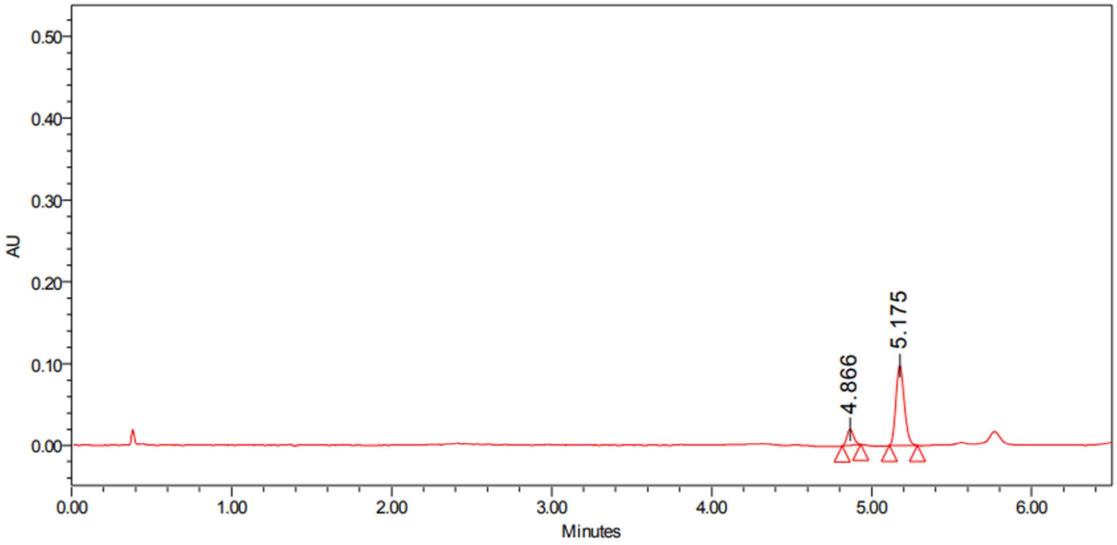

Peak Results

	RT	% Area
1	4.866	14.19
2	5.175	85.81

(1*R*,3*S*,3a*S*,9a*S*)-1-(5-Bromo-2-hydroxyphenyl)-3a,4',5',9a-tetrahydro-1*H*,2'*H*-spiro[chromeno[2,3-*c*]pyrrole-3,3'-furan]-2',9(2*H*)-dione 1u'

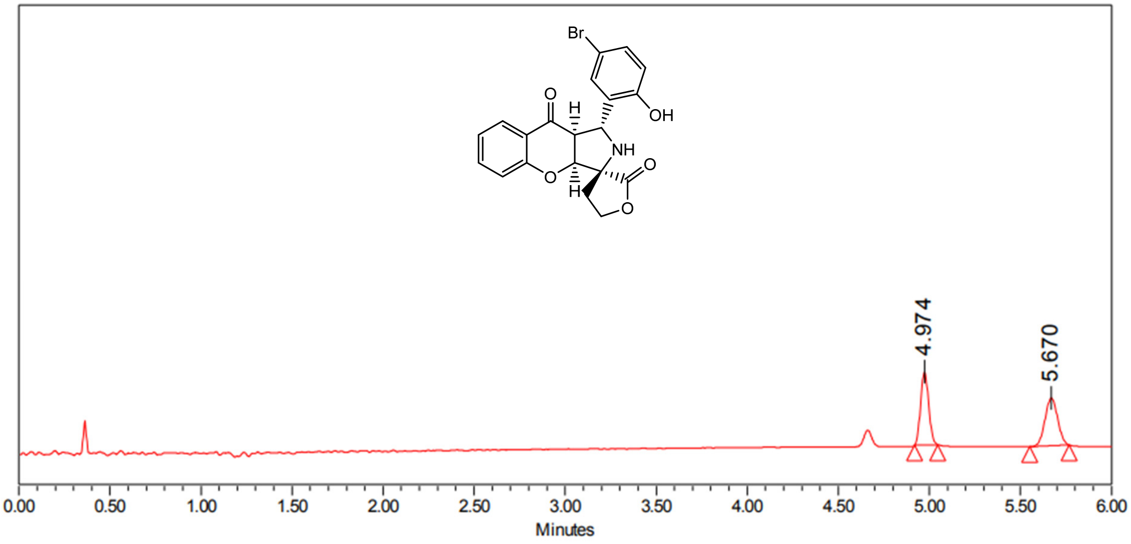

Peak Results

	RT	% Area
1	4.974	49.27
2	5.670	50.73

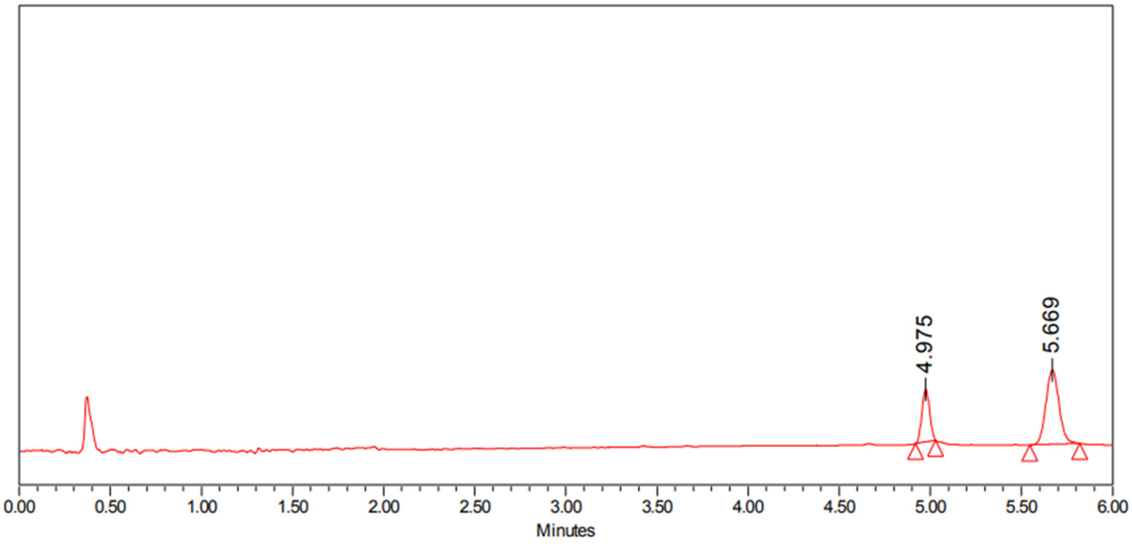

Peak Results

	RT	% Area
1	4.975	29.42
2	5.669	70.58

(1*R*,3*R*,3a*S*,9a*S*)-1-(5-Chloro-2-hydroxyphenyl)-3a,4',5',9a-tetrahydro-1*H*,2'*H*-spiro[chromeno[2,3-*c*]pyrrole-3,3'-furan]-2',9(2*H*)-dione 1w

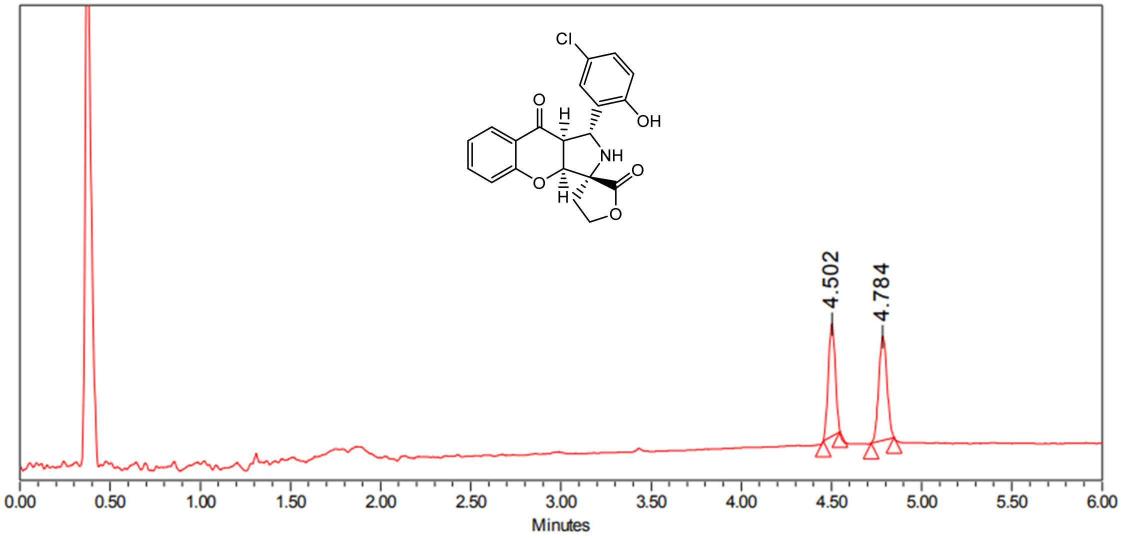

Peak Results

	RT	% Area
1	4.502	47.86
2	4.784	52.14

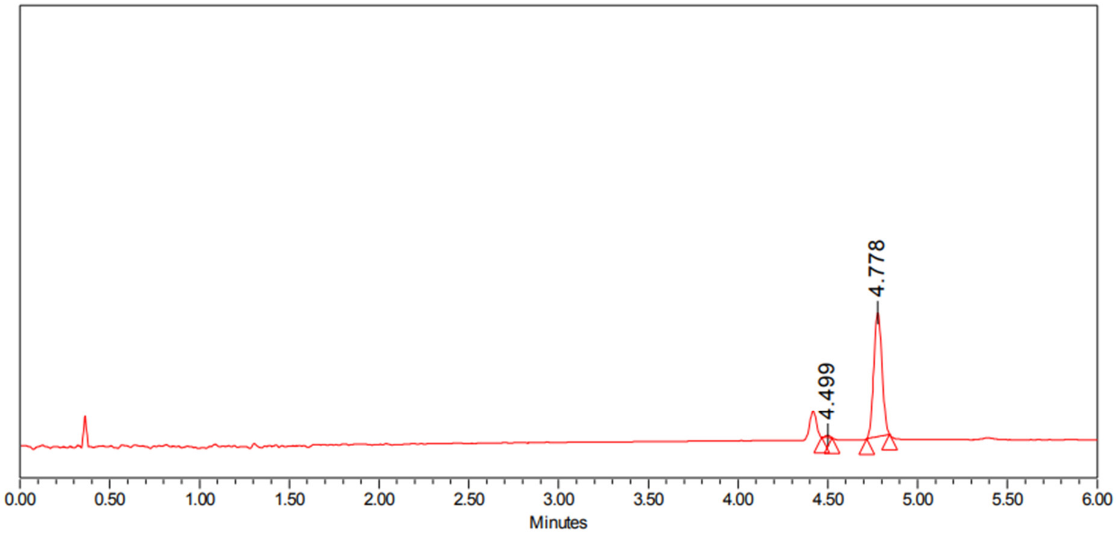

Peak Results

	RT	% Area
1	4.499	1.09
2	4.778	98.91

(1*R*,3*S*,3a*S*,9a*S*)-1-(5-Chloro-2-hydroxyphenyl)-3a,4',5',9a-tetrahydro-1*H*,2'*H*-spiro[chromeno[2,3-*c*]pyrrole-3,3'-furan]-2',9(2*H*)-dione 1w'

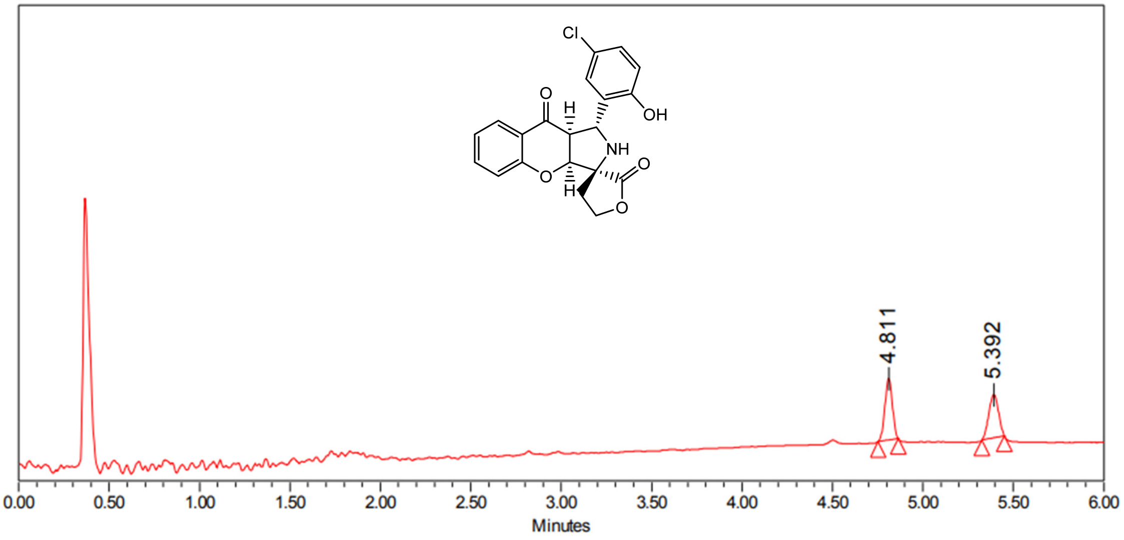

Peak Results

	RT	% Area
1	4.811	52.55
2	5.392	47.45

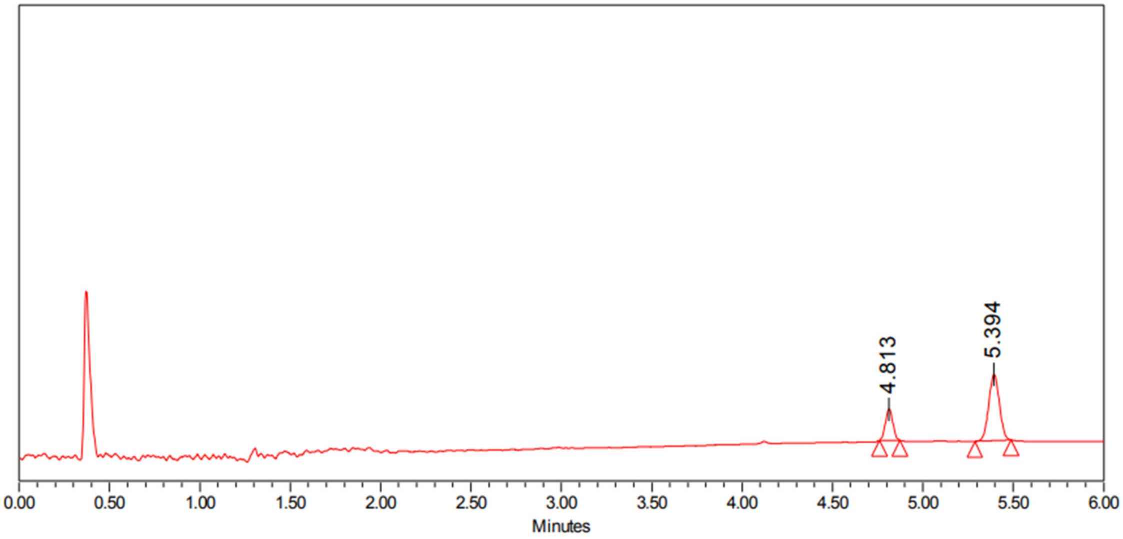

Peak Results

	RT	% Area
1	4.813	25.17
2	5.394	74.83

(1*R*,3*R*,3a*S*,9a*S*)-1-(2-Hydroxy-5-nitrophenyl)-3a,4',5',9a-tetrahydro-1*H*,2'*H*-spiro[chromeno[2,3-*c*]pyrrole-3,3'-furan]-2',9(2*H*)-dione 1x

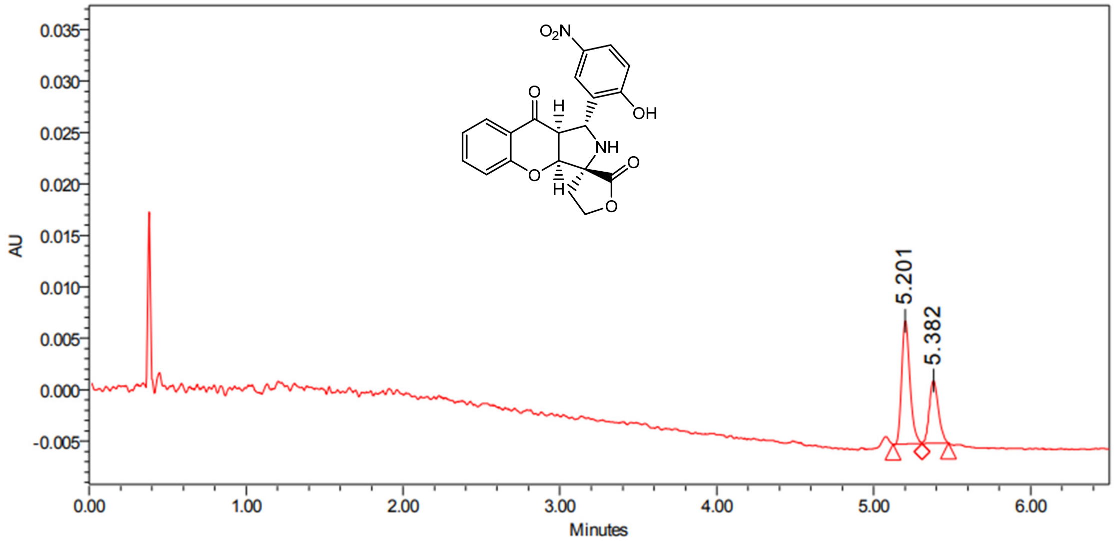

Peak Results

	RT	% Area
1	5.201	63.65
2	5.382	36.35

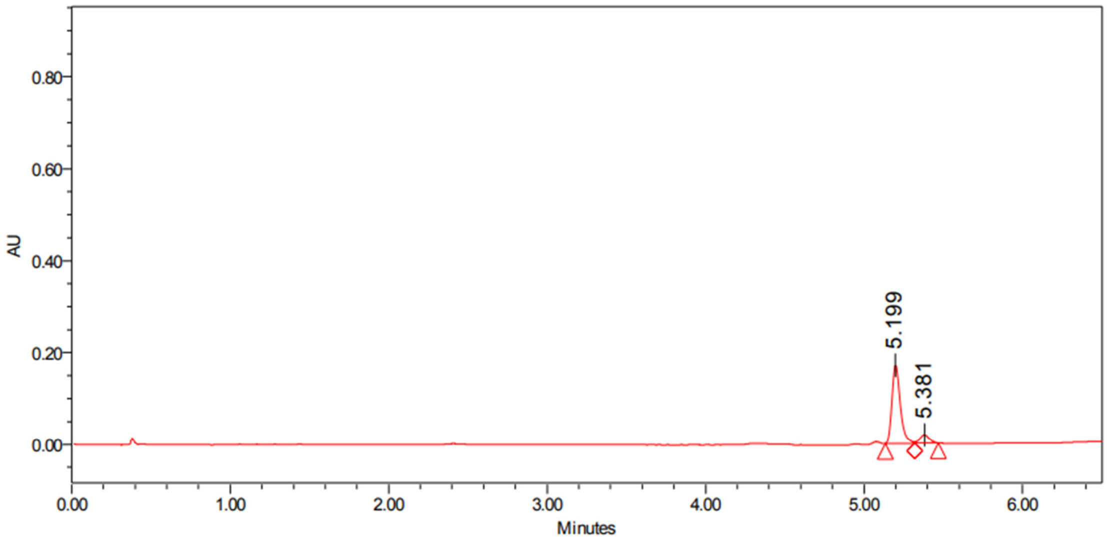

Peak Results

	RT	% Area
1	5.199	89.19
2	5.381	10.81

(1*R*,3*S*,3a*S*,9a*S*)-1-(2-Hydroxy-5-nitrophenyl)-3a,4',5',9a-tetrahydro-1*H*,2'*H*-spiro[chromeno[2,3-*c*]pyrrole-3,3'-furan]-2',9(2*H*)-dione 1x'

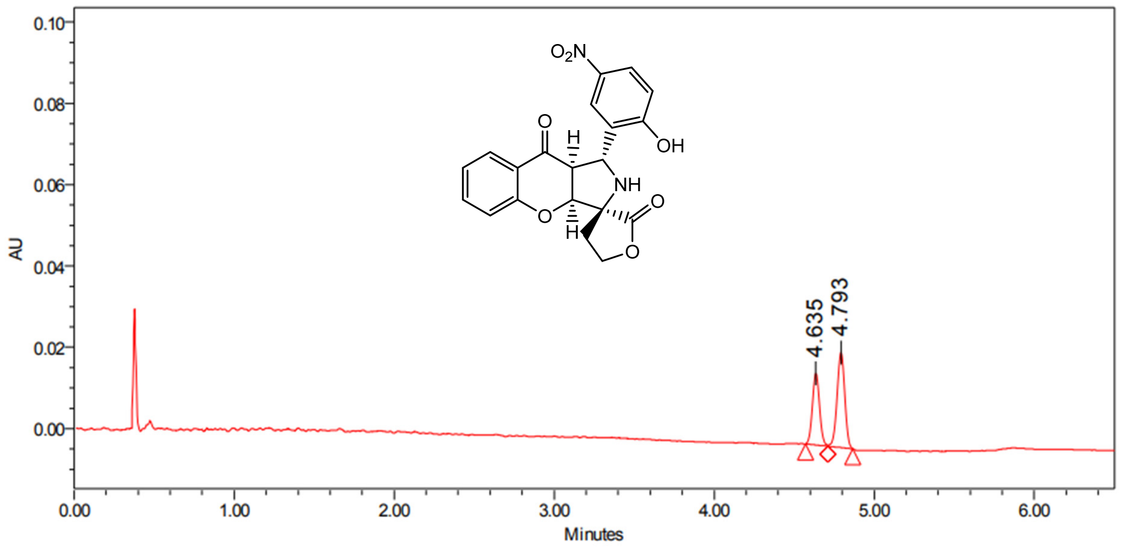

Peak Results

	RT	% Area
1	4.635	41.10
2	4.793	58.90

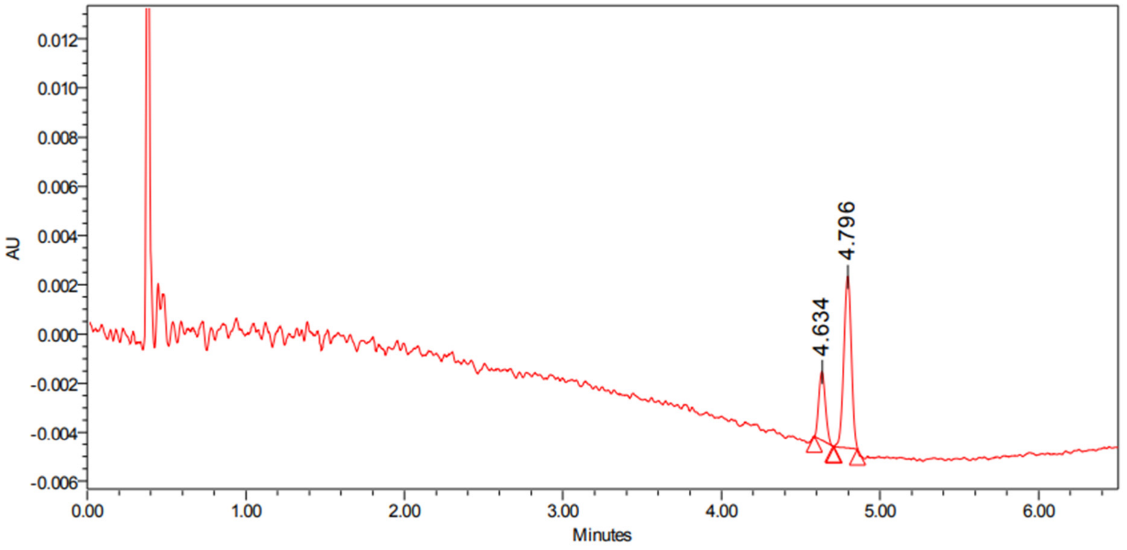

Peak Results

	RT	% Area
1	4.634	26.06
2	4.796	73.94

(8a*S*,14a*S*,14b*R*)-Diethyl 2-(*tert*-butyl)-14-oxo-8a,14,14a,14b-tetrahydrobenzo-[*e*]chromeno[3',2':3,4]pyrrolo[1,2-*c*][1,3]oxazine-8,8(6*H*)-dicarboxylate 9a

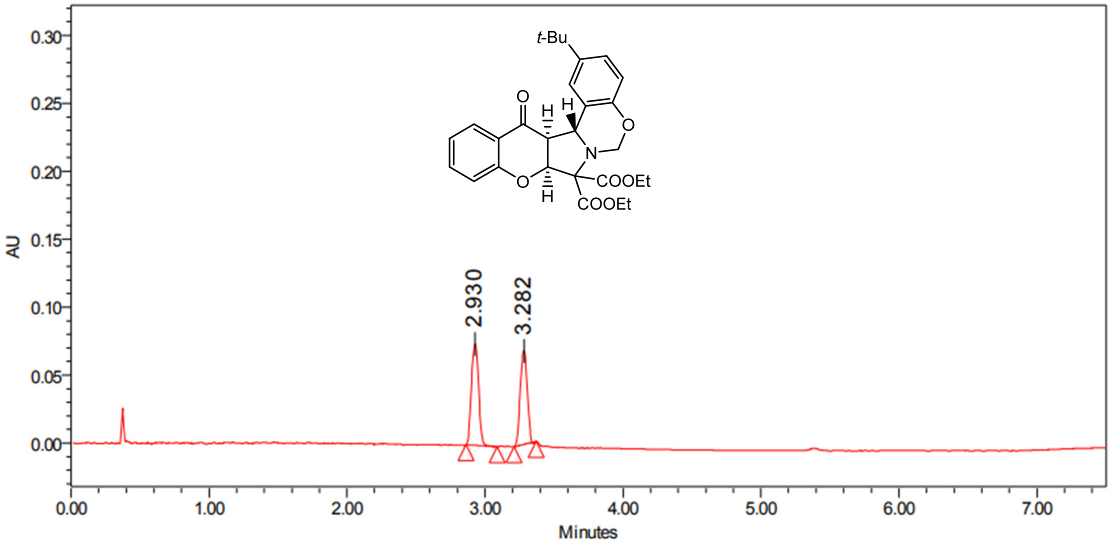

Peak Results

	RT	% Area
1	2.930	52.49
2	3.282	47.51

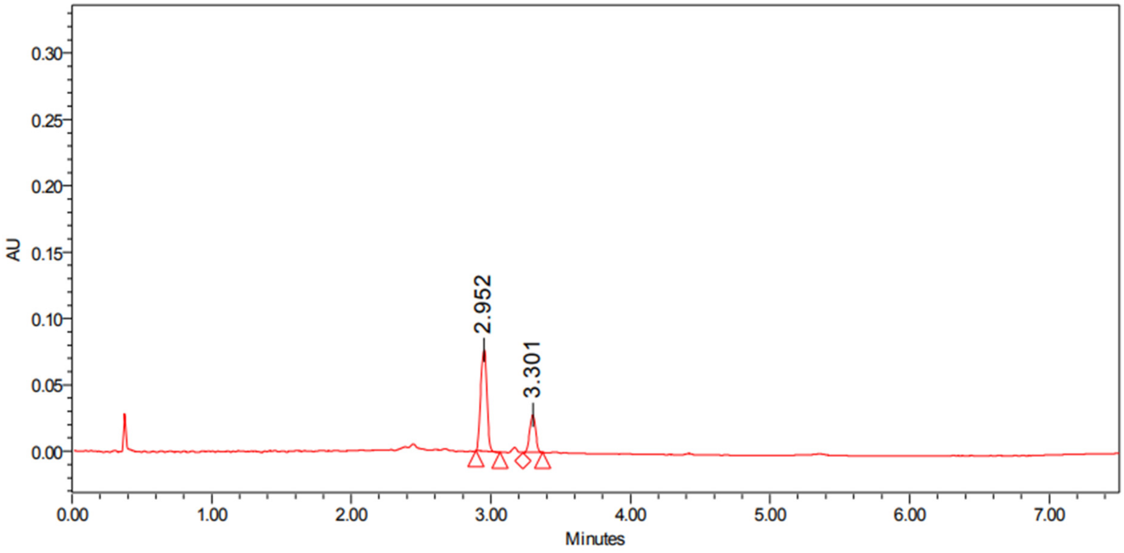

Peak Results

	RT	% Area
1	2.952	73.74
2	3.301	26.26
